# Quantification of gas-accessible microporosity in metal-organic framework glasses

**DOI:** 10.1038/s41467-022-35372-5

**Published:** 2022-12-14

**Authors:** Louis Frentzel-Beyme, Pascal Kolodzeiski, Jan-Benedikt Weiß, Andreas Schneemann, Sebastian Henke

**Affiliations:** 1 Anorganische Chemie, Fakultät für Chemie & Chemische Biologie, Technische Universität Dortmund, Otto-Hahn Straße 6, 44227 Dortmund, Germany; 2 Anorganische Chemie I, Technische Universität Dresden, Bergstrasse 66, 01069 Dresden, Germany

**Keywords:** Metal-organic frameworks, Porous materials, Solid-state chemistry

## Abstract

Metal-organic framework (MOF) glasses are a new class of glass materials with immense potential for applications ranging from gas separation to optics and solid electrolytes. Due to the inherent difficulty to determine the atomistic structure of amorphous glasses, the intrinsic structural porosity of MOF glasses is only poorly understood. Here, we investigate the porosity features (pore size and pore limiting diameter) of a series of prototypical MOF glass formers from the family of zeolitic imidazolate frameworks (ZIFs) and their corresponding glasses. CO_2_ sorption at 195 K allows quantifying the microporosity of these materials in their crystalline and glassy states, also providing excess to the micropore volume and the apparent density of the ZIF glasses. Additional hydrocarbon sorption data together with X-ray total scattering experiments prove that the porosity features of the ZIF glasses depend on the types of organic linkers. This allows formulating design principles for a targeted tuning of the intrinsic microporosity of MOF glasses. These principles are counterintuitive and contrary to those established for crystalline MOFs but show similarities to strategies previously developed for porous polymers.

## Abstract (web summary)

Glasses derived from meltable metal-organic frameworks (MOFs) offer great potential for gas separation and solid state ionics. Here, the authors quantify the porosity of a series of MOF glasses by cryogenic carbon dioxide gas sorption and propose design principles for rational tuning the glasses‘ pore size and pore volume.

## Introduction

Metal-organic frameworks (MOFs) are permanently porous crystalline materials with well-defined ordered structures, which can be precisely designed by a number of advanced synthetic concepts^1–4^. In the past two decades, MOFs have grown to a class of modular materials with widely tuneable properties for applications ranging from gas separation and storage to sensing, drug delivery and catalysis^5–9^. MOFs have also been proposed for applications in fields less common for porous materials, such as solid electrolytes and (opto)electronics^10,11^. In recent years defective, disordered and amorphous MOFs have gained more and more attention since these materials provide access to new and unusual properties beyond the state of the art^12–20^. Especially solid-to-liquid transitions of MOFs are exciting, as they offer processing and shaping of the framework materials in their liquid state (i.e. above their melting temperature, *T*_m_) and vitrification to a MOF glass after cooling below their glass transition temperature (*T*_g_)^21–25^. MOF glasses propose unique opportunities for solid-state ion conduction^20,26,27^ and gas separation membranes^28–30^ because of improved performance as a result of their monolithic structure and the absence of mass transport limiting grain boundaries^31^. However, compared to their structurally well-defined crystalline parent materials, it is very difficult to predict and design the functionally relevant porosity features (pore volume and pore size) of the MOF glasses. This is due to their highly disordered structure lacking any long-range order, thus precluding atomistic structure determination^32^. Knowledge of the atomistic structure of porous framework materials, however, is the foundation of materials design along the principles of reticular chemistry^33^.

Zeolitic imidazolate frameworks (ZIFs) are the best-investigated family of meltable and glass-forming MOFs. ZIFs are composed of tetrahedrally coordinated metal ions (typically Zn^2+^ or Co^2+^), which are interconnected by imidazolate linkers to form crystalline frameworks exhibiting strong structural similarities to inorganic zeolites^34,35^. Even though more than 250 crystalline ZIFs featuring >50 different network topologies have been reported thus far^36^, just very few of these have been demonstrated to melt and form glasses via melt-quenching. The reported ZIF glass formers include frameworks with **cag** (ZIF-4, ZIF-62, TIF-4, ZIF-UC-1 to ZIF-UC-5), **zni** (ZIF-zni) and **gis** (denoted Zn(im)_2_ (GIS)) topologies^23,37–39^ (a graphical representation of the network topologies is given in Supplementary Fig. 1). Meltable ZIFs of these topologies have further been shown to act as a flux for the melting of other ZIF structure types that are non-meltable on their own (i.e. ZIF-76, **lta** topology; ZIF-8, **sod** topology)^40–42^. Given that the crystalline ZIF precursors are typically microporous solids with porosity features interesting for gas storage and separation^43–47^, their melt-quenched glasses are deemed to exhibit similar potential for gas adsorption and separation processes^28,29^. Nevertheless, gas-accessible microporosity in MOF glasses has only been poorly investigated and was just demonstrated for a few ZIF glasses^30,37,38,48^. Quantification of the total gas-accessible pore volume of the ZIF glasses as well as a comprehensive comparison of the porosity of the glasses in relation to their crystalline precursors has not yet been reported. Understanding the porosity features of ZIF glasses, however, is of utmost importance for their application in many fields, e.g. gas separation membranes^28^ and solid electrolytes^20^.

**Fig. 1:**
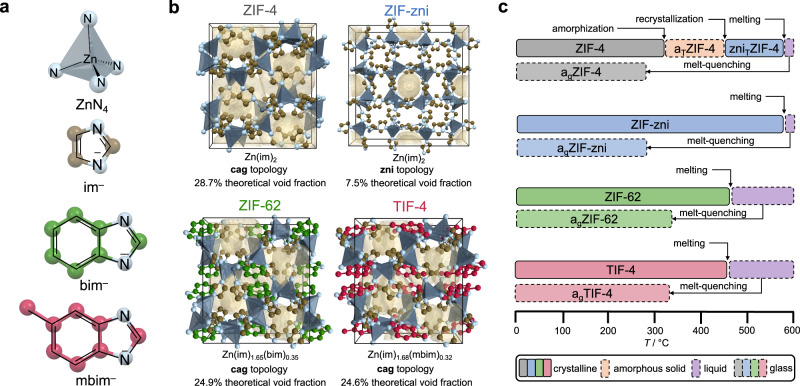
Structural representations and high-temperature phase behaviour of the investigated ZIF glass formers. **a** Building units of the investigated ZIF glass formers. **b** Crystal structures of the ZIF glass formers. ZIF-4 (CCDC code IMIDZB11), ZIF-62 (CCDC code SIWJAM) and TIF-4 (CCDC code QOSYAZ) are viewed along the crystallographic *b*-axis. ZIF-zni (CCDC code IMIDZB) is viewed along the crystallographic *c*-axis. Hydrogen atoms are omitted for clarity. The theoretical void fraction was calculated with a probe radius of 1.6 Å (see Supplementary Methods 9.4 for further details) and is shown in pale yellow. **c** Schematic representation of the high-temperature phase behaviour of the ZIF glass formers.

The prototypical MOF glass former is ZIF-4. During thermal treatment crystalline ZIF-4 first collapses to the amorphous phase a_T_ZIF-4 (a_T_ denotes thermally amorphized) at around 315 °C, followed by recrystallization to a denser polymorph of **zni** topology at ~460 °C (this phase is denoted by zni_T_ZIF-4 here, i.e. thermally recrystallized ZIF-4 with **zni** topology) and finally melting at about 580 °C (Fig. 1)^21^. Quenching the ZIF-4 melt to room temperature generates a glass named a_g_ZIF-4 (a_g_ denotes amorphous glass)^23,49^. Positron annihilation lifetime spectroscopy (PALS) revealed that a_g_ZIF-4 possesses residual microporosity^50^; a finding supported by high-level molecular dynamics simulations^51,52^. Thereon, PALS has been employed to investigate the porosity of other MOF glasses^28,40,42,53^. The method, however, is unable to prove that the detected pores in the ZIF glasses are in fact accessible to gas molecules, i.e. that the glasses possess an open framework^50^. Moreover, it is very difficult to quantify porosity (i.e. to determine the specific pore volume of the solid) with PALS^54^.

Isothermal N_2_ physisorption at 77 K or Ar physisorption at 87 K is widely applied for the quantification of porosity and surface area of crystalline as well as amorphous porous materials^55,56^. For a variety of technologically relevant amorphous porous materials (e.g. amorphous silica and amorphous carbons) cryogenic N_2_ and Ar sorption were successfully applied to get valuable insights into the porosity and thus the structure of these materials^56^. While Ar sorption of ZIF glasses has not been studied yet, previous work demonstrated that N_2_ is not adsorbed at 77 K in any ZIF glass investigated so far (i.e. a_g_ZIF-4, a_g_ZIF-62 and a_g_ZIF-UC-2 to a_g_ZIF-UC-5)^21,30,38^; a phenomenon ascribed to diffusion limitations of N_2_ gas into the narrow pores of the ZIF glasses at this low temperature^38,57^. Microporosity for some mixed-linker ZIF glasses was nevertheless demonstrated with CO_2_ physisorption measurements at 273 or 298 K with a maximum CO_2_ pressure of about 95 kPa (Supplementary Table 6). Even though such measurements provide experimental proof for gas-accessible microporosity in these ZIF glasses, they do not allow quantification of the major parameter of porosity, namely the pore volume. This is because the sorption data are collected too far away from saturation (note, a CO_2_ gas pressure of 100 kPa corresponds to a relative pressure (*p*/*p*_0_) of only about 0.03 at 273 K^57^)^58^.

In this work, we demonstrate that CO_2_ gas sorption measurements at 195 K together with various hydrocarbon sorption measurements provide deep insights into the intrinsic porosity (particularly the pore volume) of ZIF glasses. Under the established assumption that CO_2_ is present in its supercooled liquid state when confined in micropores at 195 K^59,60^, a *p*/*p*_0_ of 0.52 is reached at an absolute pressure of 100 kPa. At such a relative pressure the micropores of the porous ZIF glasses are completely filled with adsorbate, so that their specific micropore volume can be quantified from the amount adsorbed. This allows us to set the porosity of the ZIF glasses in relation to the porosity of their crystalline ZIF precursors. In addition to the canonical glass formers ZIF-4 and ZIF-zni (composition Zn(im)_2_ for both; im^–^ = imidazolate), the mixed-linker glass-forming frameworks ZIF-62 (composition Zn(im)_1.65_(bim)_0.35_; bim^–^ = benzimidazolate) and TIF-4 (composition Zn(im)_1.68_(mbim)_0.32_; mbim^–^ = 5-methylbenzimidazolate) are also studied (Fig. 1). While ZIF-4 is particularly interesting due to the series of reconstructive crystalline-to-amorphous-to-crystalline phase transitions (ZIF-4 to a_T_ZIF-4 to zni_T_ZIF-4) before melting at ~580 °C (Fig. 1c), ZIF-62 and TIF-4 are relevant because of their lower melting temperatures and more complex structure incorporating a secondary bulky imidazolate linker besides the simple im^–^ linker. The derived specific pore volumes of the ZIF glasses are directly connected to the material’s apparent density (*ρ*_app_), i.e. the density including the intrinsic microporosity, *ρ*_app_ = *m*/(*V*_frame_ + *V*_pore_); with *m* = mass, *V*_frame_ = volume of the framework, *V*_pore_ = pore volume)^61^. *ρ*_app_ is an important physical parameter, so far poorly investigated for ZIF glasses. Previous studies utilizing He pycnometry^23,38,42^ could only determine the skeletal density (*ρ*_skl_) of the glasses (that is the density excluding the intrinsic microporosity, *ρ*_skl_ = *m*/*V*_frame_). Similarly, density measurements based on Archimedes’ principle with potentially pore-filling fluids (i.e. ethanol) are also expected to yield skeletal densities^62,63^. Combining the low-temperature CO_2_ sorption data with additional hydrocarbon sorption experiments and structural insights derived from X-ray total scattering experiments allows deducing important correlations of the chemical composition of the ZIF glass former (i.e. single linker or mixed-linker materials) with the pore volume and the pore size of the derived MOF glass. Unexpectedly and most importantly, the relationship between the steric bulk of the linkers and the porosity features of the glasses is counterintuitive and found to be inverse to what is established for crystalline MOFs. The work provides a guideline for the targeted design of MOF glass porosity by a selection of the frameworks’ building blocks.

## Results

### Materials preparation and characterization

ZIF-4 (Zn(im)_2_, **cag** topology), ZIF-zni (Zn(im)_2_, **zni** topology), ZIF-62 (Zn(im)_1.65_(bim)_0.35_, **cag** topology) and TIF-4 (Zn(im)_1.68_(mbim)_0.32_, **cag** topology) were synthesized solvothermally reproducing or adapting established procedures^30^. Solvent molecules were removed from the materials at 200 °C under a dynamic vacuum yielding the activated (solvent-free) compounds. The phase purity of the crystalline ZIFs was verified by structureless profile fits (Pawley method^64^) of X-ray powder diffraction (XRPD) patterns using reference data from the literature (Supplementary Figs. 2–6)^45,65–67^. The complete removal of solvents from the pores is demonstrated by ^1^H nuclear magnetic resonance (NMR) and Fourier-transform infra-red (FTIR) spectroscopy data (see Supplementary Methods 3, 4 for further details). ^1^H NMR spectroscopy was further used to determine the linker ratio of the ZIF-62 and TIF-4 samples, leading to the chemical compositions given above (Supplementary Figs. 21, 23).

**Fig. 2:**
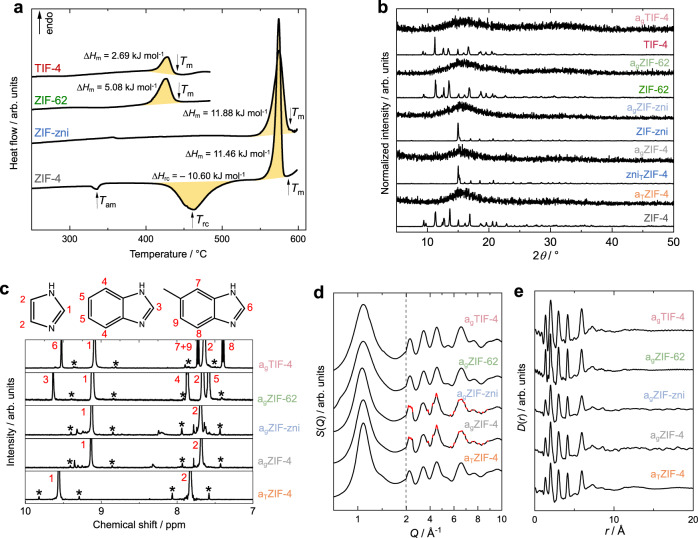
Thermal and structural characterization of the ZIF glass formers and their corresponding glasses. **a** DSC data of the ZIF glass formers. **b** XRPD patterns of all investigated materials. The patterns are vertically offset for clarity. **c** Stacked plot of ^1^H NMR spectra of digested samples of the investigated amorphous materials (recorded in a solvent mixture of DMSO-*d*_6_ and DCl/D_2_O at room temperature). A zoom in the aromatic region is shown. The ^13^C satellite peaks of protons 1 and 2 are marked with asterisks (*). Weak signals not marked with asterisks belong to unidentified decomposition products. **d** X-ray total scattering data in the form *S*(*Q*) of the amorphous phases. For a_g_ZIF-4 and a_g_ZIF-zni sharp scattering features ascribed to ZnO impurities are highlighted in red. **e** XPDFs in form *D*(*r*) obtained from the *S*(*Q*) data shown in **d**. Data for a_g_ZIF-62 in panels **d** and **e** are taken from ref. 30.

Based on the four crystalline reference materials, the corresponding thermal products (a_T_ZIF-4, zni_T_ZIF-4, a_g_ZIF-4, a_g_ZIF-zni, a_g_ZIF-62 and a_g_TIF-4, Fig. 1c) were obtained via thermal treatment under inert atmosphere in a thermogravimetric analysis/differential scanning calorimetry (TGA/DSC) apparatus. DSC data served as a guide for selecting the right temperature protocol to obtain the glasses, as well as the intermediate compounds a_T_ZIF-4 and zni_T_ZIF-4 in the case of ZIF-4 (Fig. 2a, Supplementary Table 2). Heating and cooling rates have been +10 and –10 °C min^−1^, respectively. The temperature profiles and corresponding TGA/DSC data yielding the corresponding products are summarized in the Supplementary Information (Supplementary Table 4, Supplementary Figs. 27–32). We note, that for the preparation of a_g_ZIF-4 and a_g_ZIF-zni an isothermal segment (10 min) at a maximum temperature of 578 °C was required to obtain amorphous glasses without crystalline residues of ZIF-zni (Supplementary Fig. 12).

All products were characterized with XRPD and X-ray total scattering, ^1^H NMR and FTIR spectroscopy, as well as DSC, and the obtained data are in agreement with the literature^21,23,30,39,53,67,68^. XRPD data of the amorphous materials show only diffuse scattering and no sharp Bragg peaks (Fig. 2b, Supplementary Figs. 7–11). A profile fit to the XRPD pattern of zni_T_ZIF-4 testifies the recrystallization of a_T_ZIF-4 to the Zn(im)_2_ phase with **zni** topology (Supplementary Fig. 4). The glasses a_g_ZIF-4, a_g_ZIF-zni, a_g_ZIF-62 and a_g_TIF-4 further display particle coalescence characteristic for melt-quenched glasses (Supplementary Figs. 33–36). It is noteworthy that ^1^H NMR data of digested ZIF samples demonstrate the full integrity of the organic linkers after thermal treatment, except for a_g_ZIF-4 and a_g_ZIF-zni (Fig. 2c, Supplementary Figs. 16–18, 20, 22, 24). For the latter glasses, weak signals for impurities are visible in their ^1^H NMR spectra. These impurities are ascribed to the presence of some decomposition products which are a consequence of the higher maximum processing temperature (578 °C) of these two glass materials compared to a_g_ZIF-62 and a_g_TIF-4 (maximum processing temperature is 475 °C for those glasses). Partial decomposition of a_g_ZIF-4 and a_g_ZIF-zni is further indicated by weak scattering signals ascribed to crystalline ZnO impurities in their X-ray total scattering functions *S*(*Q*) (Fig. 2d, Suppplementary Figs. 41, 43), mass losses of about 6% during glass preparation in the TGA/DSC experiment (Supplementary Figs. 29, 30), as well as the dark colour of these glasses (Supplementary Figs. 33, 34). The other three amorphous materials (a_T_ZIF-4, a_g_ZIF-62 and a_g_TIF-4) show neither signs of decomposition of the organic linkers nor the formation of crystalline by-products.

X-ray pair distribution functions (XPDFs) in the form of *D*(*r*) derived via Fourier-transformation of the total scattering functions *S*(*Q*)^69^ of all crystalline ZIFs and their thermal products demonstrate that the short-range structure of the crystalline phases (that is Zn^2+^ ions surrounded by four imidazolate-type linkers) is preserved in all amorphous phases (Fig. 2d, e, see Supplementary Methods 8 and Supplementary Figs. 41, 42). The last sharp peak in the XPDFs of the amorphous materials is visible at about 5.9 Å and corresponds to the distance of two neighbouring Zn^2+^ ions in the networks. The XPDFs of a_T_ZIF-4, a_g_ZIF-62 and a_g_TIF-4 show some weaker correlations for *r* > 5.9 Å, which are indicative of some medium-range order (MRO). At *r* > 15 Å, *D*(*r*) of these materials converges towards zero. In contrast, the XPDFs of a_g_ZIF-4 and a_g_ZIF-zni show additional weak but significant pair correlations extending well beyond *r* = 20 Å (Supplementary Fig. 42). These correlations are ascribed to the crystalline ZnO impurities present in these ZIF glasses (Supplementary Fig. 43).

With the aim to get more insights into the structure of the amorphous ZIF derivatives, we take a closer look at the first sharp diffraction peak (FSDP) of their scattering functions *S*(*Q*) (Fig. 2d). The FSDP contains valuable information about the MRO of amorphous network solids^70–72^. For the ZIF glasses, MRO means some degrees of order extending beyond the first Zn–Zn neighbour distance. The position of the FSDP (*Q*_FSDP_) has been associated with a real space correlation length between the strongest scattering centres (i.e. Zn^2+^ cations here), which are surrounded by interstitial voids^73,74^. As such, *Q*_FSDP_ could be regarded as a reciprocal space signature for the glass networks’ porosity and density. The full width at half maximum of the FSDP (∆*Q*_FSDP_) is inversely proportional to the real space coherence length over which the MRO (i.e. the correlation) exists^73,74^. We fitted the FSDP of the five amorphous materials under study here to a pseudo-Voigt function to derive *Q*_FSDP_ and ∆*Q*_FSDP_ (see Supplementary Methods 8.1). *Q*_FSDP_ is identical for all solids found at about 1.11 Å^–1^, suggesting the densities of a_T_ZIF-4 and the four glasses are similar. Remarkably, ∆*Q*_FSDP_ is significantly larger for a_g_ZIF-62 and a_g_TIF-4 (∆*Q*_FSDP_ = 0.35 Å^–1^) than for the amorphous ZIF-4/ZIF-zni derivatives (∆*Q*_FSDP_ = 0.24–0.29 Å^–1^). This translates to a shorter coherence length for the MRO in the mixed-linker ZIF glasses. Hence, a_g_ZIF-62 and a_g_TIF-4 are more disordered than the single-linker materials, which can be explained by their more complex chemical composition involving two different imidazolate-type linkers with different steric bulk.

### N_2_ and Ar physisorption studies

N_2_ sorption isotherms at 77 K were collected for all crystalline and amorphous materials (Supplementary Figs. 45, 46). Only crystalline ZIF-4 (the most porous ZIF under study here) adsorbs large amounts of N_2_, while all other compounds (including crystalline ZIF-62 and TIF-4) show negligible adsorption of N_2_ at 77 K, preventing the determination of the material’s pore volume. These observations are in accordance with available N_2_ sorption data of ZIF glasses and point towards strong N_2_ diffusion limitations at 77 K due to the narrow micropores of these ZIFs^14,21,30^. Additional N_2_ sorption isotherms recorded at 195 K suggest that N_2_ is able to access some of the narrow micropores of the ZIF glasses, however, the recorded uptakes are again rather low (0.25–0.45 mmol g^–1^ at 95 kPa) and large hystereses are apparent on the desorption branch, indicating that the diffusion of N_2_ in and out of the pores of the ZIF glasses is still hindered at this temperature (Supplementary Fig. 47). Similarly, Ar sorption isotherms recorded at 87 K covering a *p*/*p*_0_ range from 0 to 1, prove that the narrow micropores of the ZIF glasses are largely inaccessible to Ar at this temperature (Supplementary Fig. 48).

### CO_2_ physisorption studies and pore volume determination

**Fig. 3:**
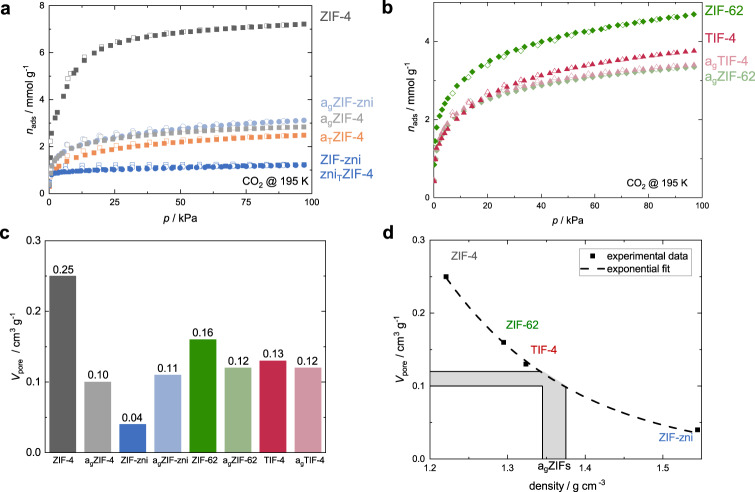
CO_2_ gas sorption analysis. **a** CO_2_ sorption isotherms collected at 195 K of ZIF-4 and ZIF-zni and their corresponding thermal products. ZIF-4 derivatives are shown as squares. ZIF-zni derivatives are shown as circles. **b** CO_2_ isotherms collected at 195 K of ZIF-62 and TIF-4 and their corresponding glasses. ZIF-62 derivatives are shown as rhombs. TIF-4 derivatives are shown as triangles. In panels **a** and **b** adsorption and desorption, branches are shown as closed and open symbols, respectively. **c** Bar plot of the micropore volumes (*V*_pore_) for all crystalline ZIFs and their corresponding glasses derived from the CO_2_ isotherms at 195 K. **d** Plot of density against *V*_pore_ of all crystalline ZIFs with the exponential fit. The ZIF glass bulk densities can be estimated based on their experimentally obtained *V*_pore_ (see light grey area).

In order to get around the gas diffusion limitations, the porosity of all materials was probed by CO_2_ physisorption at 195 K. The smaller kinetic diameter of the CO_2_ molecule (3.30 Å^75^) compared to N_2_ (3.64 Å^75^) and Ar (3.40 Å^75^) together with the higher temperature (195 vs. 77 K) facilitates the diffusion of the gas into narrow micropores (size < 5 Å)^76,77^. Compared to the CO_2_ sorption experiments performed at 273 or 298 K, running the experiment at 195 K allows complete filling of the micropores at 100 kPa^78^. In a non- or macroporous system, CO_2_ solidifies at the given conditions. In microporous materials (i.e. pores with a size ≤ 20 Å), CO_2_ is considered to be in its supercooled liquid state so that the micropores are effectively filled with fluid CO_2_ and not with solid CO_2_^59,60^. The saturation pressure (*p*_0_) for the gas/supercooled-liquid equilibrium is significantly higher (*p*_0_ = 191 kPa at 195 K) than *p*_0_ for the gas/solid equilibrium (*p*_0_ = 100 kPa at 195 K)^79^. Hence, the maximum *p*/*p*_0_ achievable in a CO_2_ sorption experiment of microporous materials at 195 K is ~0.5. Nevertheless, at this relative pressure, all pores up to a width of 50 Å are completely filled^77,78^. Consequently, the maximum gas capacities (${n}_{{{{{{\rm{ads}}}}}}}^{{{\max }}}$) determined at 95 kPa (*p*/*p*_0_ ≈ 0.5) can be used to determine the specific micropore volume (*V*_pore_) of the investigated materials by taking into account the density of supercooled liquid CO_2_ at 195 K (Table 1, Fig. 3, see Supplementary Methods 9.2 for further details).

**Table 1:** Summary of maximum gas capacities (${{n}}_{{{\rm {ads}}}}^{{ {{\max }}}}$), specific micropore volumes (*V*_pore_) and BET areas (*S*_BET_) obtained from the CO_2_ gas physisorption isotherms collected at 195 K together with the calculated densities (${{{{{\boldsymbol{\rho }}}}}}$) for all investigated materials

Compound	${n}_{{{{{{\rm{ads}}}}}}}^{{{\max }}}\,[{\rm {mmol}}\,{\rm {g}}^{-1}]$	$V_{{{\rm{pore}}}}[{{{\rm{cm}}}}^{3}\,{{{\rm{g}}}}^{-1}]$	$S_{{{\rm{BET}}}}[{{{\rm{m}}}}^{2}\,{{{\rm{g}}}}^{-1}]$	$\rho\,[{\rm {g}}\,{\rm {cm}}^{-3}]$
ZIF-4	7.22	0.25	490	1.22^a^
a_T_ZIF-4	2.49	0.09	145	1.39^b^
zni_T_ZIF-4	1.23	0.04	92	1.54^a^
a_g_ZIF-4	2.84	0.10	187	1.38^b^
ZIF-zni	1.21	0.04	94	1.54^a^
a_g_ZIF-zni	3.12	0.11	189	1.36^b^
ZIF-62	4.70	0.16	264	1.29^a^
a_g_ZIF-62	3.34	0.12	200	1.35^b^
TIF-4	3.76	0.13	185	1.32^a^
a_g_TIF-4	3.40	0.12	204	1.35^b^

^a^Crystallographic density.

^b^Apparent density (*ρ*_app_) calculated from CO_2_ sorption data.

Brunauer–Emmett–Teller (BET) specific surface areas^80^ have also been determined from the low-temperature CO_2_ sorption isotherms (Table 1, see Supplementary Methods 9.2 for further details). Even though utilization of the BET model is very common in research on porous materials, we note that BET areas determined from CO_2_ sorption data recorded at 195 K are typically much lower than values determined via N_2_ sorption at 77 K^58^. In general, the BET model is not applicable to the microporous materials studied here^57^. For CO_2_ sorption data recorded at 195 K, the application of the BET model is even more problematic due to the uncertainty of the effective cross-sectional area of the CO_2_ molecule. Thus, we provide the BET areas of the ZIF materials only for reference.

We first concentrate on ZIF-4, ZIF-zni and their corresponding amorphous and crystalline high-temperature phases. Remarkably, all these phases adsorb CO_2_ as evident from the Typ I (or Langmuir) shaped isotherms (Fig. 3a). As expected, the crystalline ZIF-4 exhibits the highest *V*_pore_ of 0.25 cm^3^ g^–1^ (Table 1). Going along the other ZIF-4 phases consecutively formed via thermal treatment, we first see a drastic decrease in *V*_pore_ to 0.09 cm^3^ g^–1^ for a_T_ZIF-4 (–64% compared to ZIF-4), demonstrating a collapse and densification of the framework but the preservation of about 36% of the pore space of the crystalline phase. *V*_pore_ drops further for the recrystallized zni_T_ZIF-4 (*V*_pore_ = 0.04 cm^3^ g^–1^) to only about 16% of the porosity of crystalline ZIF-4. Noteworthy, the solvothermally synthesized ZIF-zni features the same pore volume (*V*_pore_ = 0.04 cm^3^ g^–1^) as zni_T_ZIF-4, establishing that a_T_ZIF-4 completely recrystallizes to zni_T_ZIF-4 during thermal treatment. This is in line with the very similar melting enthalpies (∆*H*_melt_) of zni_T_ZIF-4 (11.46 kJ mol^−1^) and ZIF-zni (11.88 kJ mol^−1^) determined via DSC (Fig. 2a and Supplementary Table 3). Since ZIF-zni, which is the densest and most stable crystalline Zn(im)_2_ phase (at least at temperatures >360 °C^81^), is already microporous, it is not surprising that a_g_ZIF-4 and a_g_ZIF-zni also adsorb CO_2_. Both glasses show very similar CO_2_ sorption isotherms with specific pore volumes of 0.10 and 0.11 cm^3^ g^–1^, respectively.

The micropore volume of the isoreticular crystalline glass precursors ZIF-4, ZIF-62 and TIF-4 decrease from 0.25 cm^3^ g^–1^ (ZIF-4) to 0.16 cm^3^ g^–1^ (ZIF-62) to 0.13 cm^3^ g^–1^ (TIF-4). This correlates with the implementation of the secondary bulky imidazolate linkers (bim^–^ and mbim^–^) in ZIF-62 and TIF-4, reducing the void space of the crystalline framework (see void fractions calculated based on the crystal structures shown in Fig. 1b). We note that the concentration of the bim^–^ and mbim^–^ linkers in the mixed linker MOFs is rather similar (ZIF-62: Zn(im)_1.65_(bim)_0.35_; TIF-4: Zn(im)_1.68_(mbim)_0.32_), while the steric bulk of mbim^–^ (containing an additional methyl group) is higher compared to bim^–^. This further decreases the free void space in crystalline TIF-4 and explains the higher *V*_pore_ of ZIF-62 over TIF-4 (Fig. 3c).

Remarkably, the pore volumes of the mixed-linker glasses a_g_ZIF-62 (*V*_pore_ = 0.12 cm^3^ g^–1^) and a_g_TIF-4 (*V*_pore_ = 0.12 cm^3^ g^–1^) are slightly higher than for a_g_ZIF-4 and a_g_ZIF-zni. This is counterintuitive by comparing the pore volumes of the respective crystalline parent materials. The highest reduction in *V*_pore_ (~60%) from the crystalline to the glass material is found for ZIF-4, while only a reduction of 25% and 8% is found for the glasses of ZIF-62 and TIF-4. This observation suggests that a_g_ZIF-62 and a_g_TIF-4 have a pore structure that is more similar to that of their crystalline precursors, while a_g_ZIF-4 differs strongly from crystalline ZIF-4.

It must be noted that the presence of small amounts of decomposition products (ZnO and decomposed organic linkers) in a_g_ZIF-4 and a_g_ZIF-zni could potentially reduce the specific gas capacities and the corresponding *V*_pore_. Nevertheless, the thermally amorphized phase a_T_ZIF-4, which does not contain any decomposition products, features an even lower pore volume (*V*_pore_ = 0.09 cm^3^ g^–1^) than a_g_ZIF-4 and a_g_ZIF-zni, suggesting that their partial decomposition only has a minor influence on *V*_pore_. Moreover, the finding that *V*_pore_ of a_T_ZIF-4, a_g_ZIF-4 and a_g_ZIF-zni are similar is in line with our analysis of the FSDP of the scattering function, as well as the previous observation that a_T_ZIF-4 and a_g_ZIF-4 are located at the same place on the potential energy landscape of Zn(im)_2_^21^.

For comparison, we collected additional CO_2_ sorption isotherms of all materials at 273 K (Supplementary Figs. 49, 50). Here, we see again the highest capacity for ZIF-4 (2.64 mmol g^–1^) followed by ZIF-62 (1.87 mmol g^–1^) and TIF-4 (1.43 mmol g^–1^) whereas the amorphous materials (a_T_ZIF-4, a_g_ZIF-4, a_g_ZIF-zni, a_g_ZIF-62, a_g_TIF-4), as well as the materials adopting the zni topology (zniTZIF-4, ZIF-zni), show lower uptakes in the range from 0.81 to 1.21 mmol g^–1^ (Supplementary Table 6). As expected, the drastic differences in *V*_pore_ of the various ZIF materials are not observable in the CO_2_ sorption data collected at 273 K, since data are only collected up to about 0.03*p*/*p*_0_. Previous works used CO_2_ sorption data of ZIF glasses recorded at 273 K to calculate pore size distributions (PSDs) of the materials with the nonlocal density functional theory (NLDFT) method^82^ applying a model for carbon materials with slit pores. In our analysis, we found that the PSDs for the crystalline ZIFs derived by this model are in strong disagreement with the theoretical PSDs calculated based on the corresponding crystal structures (Supplementary Methods 9.3). The disagreement is explained by a mismatch of pore geometry and effective adsorption potential of the ZIFs with the carbon-based NLDFT model. Since the NLDFT model cannot reproduce the theoretical PSDs of the crystalline ZIFs, we conclude that this model is inadequate to examine the PSDs of the ZIF glasses as well. We hope that future statistical models for CO_2_ gas sorption will be able to safely reproduce the porous features of crystalline ZIFs so that these models could also be used to shed light on the pore size distribution of ZIF glasses^83^.

### Density determination

The *V*_pore_ quantified from the CO_2_ sorption data recorded at 195 K allows the determination of *ρ*_app_ of the ZIF glasses. As stated above, *ρ*_app_ of porous ZIF glasses is unknown so far, since He pycnometry (the established method for density determination of small volume powder samples) only allows determining *ρ*_skl_ (He penetrates also into the micropores of the materials)^84^. Nevertheless, *ρ*_app_ is one of the key material properties not only for technological applications but also as a boundary condition for computational modelling of such non-equilibrium amorphous materials^51,85^.

Here, *ρ*_app_ of the ZIF glasses is determined according to the following procedure. At first, we calculated the theoretical void fraction (tVF) for crystalline ZIF-4 and ZIF-zni based on their crystal structures (tVF_ZIF-4_ = 28.7%, tVF_ZIF-zni_ = 7.5%; Fig. 1b) and compared these values to the experimental void fraction (eVF = *V*_pore_·*ρ*_cryst_) calculated from *V*_pore_ and the crystallographic densities (*ρ*_cryst_; see Supplementary Methods 9.4 for further details). For both crystalline ZIFs, we found a good agreement between theory and experiment (eVF_ZIF-4_ = 30.6%, eVF_ZIF-zni_ = 6.2%). We note that a similar comparison of the tVFs to the eVFs for crystalline ZIF-62 and TIF-4 is challenging due to the disorder of the secondary bulky linkers in their crystal structures. Subsequently, the *V*_pore_ of the crystalline ZIF-4, ZIF-zni, ZIF-62 and TIF-4 was plotted against their *ρ*_cryst_ (Fig. 3d). The data could be very well fitted with an exponential function (*R*^2^ = 0.998, see Supplementary Methods 9.4), which then is used to calculate *ρ*_app_ of the glasses from their experimental pore volumes (Table 1). In correspondence to their quite similar *V*_pore_, we find comparable apparent densities for all glasses in the range from 1.35 to 1.38 cm^3^ g^–1^. Importantly, these densities are up to 20% lower than the skeletal densities (*ρ*_skl_) previously determined for ZIF glasses by He pycnometry^23^. Additionally, our data suggest that the density values previously obtained via Archimedes’ principle are also too high^62,63^, since ethanol was utilized as the soaking solvent which might also penetrate (at least partially) into the glasses’ micropores. Based on the pore volumes and densities derived by our method, we further estimate a void fraction between 14% and 16% for the ZIF glasses (see Supplementary Methods 9.2).

### Hydrocarbon physisorption studies

**Fig. 4:**
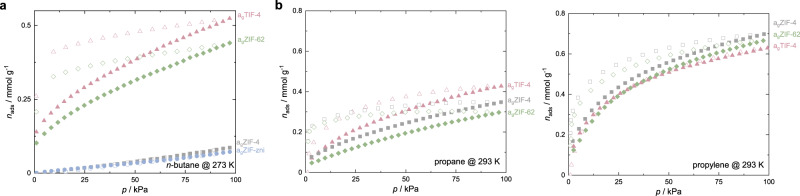
Hydrocarbon sorption studies. **a** *n*-butane sorption isotherms of the ZIF glasses collected at 273 K. **b** Propane and propylene sorption isotherms collected at 293 K. In all panels, adsorption and desorption branches are shown as closed and open symbols, respectively.

Since reliable information on the PSDs of the ZIF glasses could not be obtained from the CO_2_ sorption isotherms, we intended to get deeper insights into their pore sizes via hydrocarbon sorption (*n*-butane at 273 K, propane and propylene at 293 K, Fig. 4). In accordance with our previous study on hydrocarbon sorption in a_g_ZIF-62^30^, we found comparable adsorption of *n*-butane for a_g_ZIF-62 and a_g_TIF-4. The isotherms are of Type I shape (Langmuir), typical for adsorption in microporous solids, and feature a strong hysteresis, signifying diffusion limitations of *n*-butane in the narrow micropores of the ZIF glasses. Calculation of the void volume occupied by *n*-butane close to saturation (*p* ≈ 95 kPa, *p*/*p*_0_ ≈ 0.95) and comparison with the void volume determined via CO_2_ sorption signifies that *n*-butane is only able to access about 35–42% of the void space available for CO_2_ in a_g_ZIF-62 and a_g_TIF-4 (Supplementary Table 9).

Surprisingly, a_g_ZIF-4 and a_g_ZIF-zni do not adsorb any *n*-butane in their pores but only on the external surface, resulting in isotherms with very little uptake. The contrasting sorption properties of a_g_ZIF-4 and a_g_ZIF-zni compared to a_g_ZIF-62 and a_g_TIF-4 indicate important differences in the pore structure of these glasses. The larger kinetic diameter of *n*-butane (4.3 Å)^75^ compared to CO_2_ (3.3 Å)^75^ signifies that the pore limiting diameter of a_g_ZIF-4 and a_g_ZIF-zni is smaller than the one of the glasses featuring a secondary bulky imidazolate linker (a_g_ZIF-62 and a_g_TIF-4). It appears that this difference is not originating from the higher processing temperatures and the slight decomposition of a_g_ZIF-4 and a_g_ZIF-zni, since a_T_ZIF-4 (thermally amorphized at 379 °C, no decomposition) also only adsorb *n*-butane on its external surface and not in the micropores (Supplementary Fig. 53). Thus, the presence of a secondary bulky imidazolate linker appears to be a key parameter for the pore size and pore limiting diameter of the ZIF glasses. Our data indicate that the amorphous frameworks of the composition Zn(im)_2_ (i.e. a_T_ZIF-4, a_g_ZIF-4 and a_g_ZIF-zni) exhibit narrower pore openings than the framework glasses containing a secondary bulky imidazolate linker (i.e. a_g_ZIF-62 and a_g_TIF-4). Interestingly, small amounts of *n*-butane (0.14 mmol g^–1^) are adsorbed in a_g_ZIF-4 at 293 K and 95 kPa, suggesting that the higher kinetic energy of the gas molecules at 293 K allows some penetration in the pores of the glass, even though the *n*-butane uptake of a_g_ZIF-4 is still significantly lower than the *n*-butane uptake of the mixed-linker ZIF glasses (Supplementary Fig. 56, Supplementary Table 7).

In contrast, propane (kinetic diameter 4.3 Å) and propylene (kinetic diameter 4.5 Å) sorption isotherms at 293 K feature a Langmuir-shape (typical for adsorption in micropores) and show comparable uptakes for all the investigated ZIF glasses (Fig. 4b, c, Supplementary Table 7). In accordance with our recent findings, we see a much higher adsorption affinity for propylene over propane^30^ (Supplementary Figs. 57–59). This trend is already present in the crystalline parent materials ZIF-62 and TIF-4 (Supplementary Figs. 58, 59). Except for propylene sorption in a_g_TIF-4, significant hystereses again point towards gas diffusion limitations, further confirming the narrow micropores of the ZIF glasses. Additional propane and propylene sorption isotherms recorded at 313 K show that the uptake of both gases at a pressure of 95 kPa is reduced compared to 293 K, while a strongly hysteretic sorption behaviour is still apparent (Supplementary Figs. 60, 61, Supplementary Table 7). Hence, diffusion of these gases in and out of the glass frameworks is still kinetically hindered at 313 K.

## Discussion

We investigated the porosity of a compositional series of ZIF glasses in detail, determined their micropore volumes, and compared them to their crystalline framework precursors. This series includes ZIF materials containing only the small im^–^ linker (ZIF-4 and ZIF-zni) and others, featuring a secondary bulky imidazolate linker (bim^–^ or mbim^–^) next to im^–^ (ZIF-62 and TIF-4). All glasses are found to exhibit CO_2_-accessible microporosity and specific pore volumes in the range from 0.10 to 0.12 cm^3^ g^–1^. This also includes a_g_ZIF-4 and a_g_ZIF-zni whose open pore space was so far understood as being inaccessible for gases^21,50^. We thus conclude that microporosity is an intrinsic feature of the class of ZIF glasses, which is a huge benefit compared to conventional inorganic glasses, whose microporosity typically must be generated via elaborate post-treatment methods (e.g. leaching procedures)^86^.

The specific pore volumes of the ZIF glasses provided a means to calculate the apparent densities of these glasses. The apparent densities are much lower than the previously reported skeletal densities of ZIF glasses and lie in between the density of the most porous glass former ZIF-4 and its most dense (but still microporous) polymorph ZIF-zni. The fact that the density of a_g_ZIF-62 (*ρ*_app_ = 1.35 cm^3^ g^–1^) is higher than the density of crystalline ZIF-62 (*ρ*_cryst_ = 1.29 cm^3^ g^–1^) is in accordance with the negative Clapeyron-slope behaviour reported for ZIF-62 (i.e. melting point lowering with increasing pressure)^87^. Our data suggest a similar behaviour for TIF-4 (*ρ*_cryst_(TIF-4) = 1.32 g cm^–3^, *ρ*_app_(a_g_TIF-4) = 1.35 g cm^–3^). In turn, the higher density of ZIF-zni (*ρ*_cryst_(ZIF-zni) = 1.54 g cm^–3^) compared to the corresponding glass (*ρ*_app_(a_g_ZIF-zni) = 1.36 g cm^–3^) suggest a positive slope of the melting curve and thus conventional melting behaviour (i.e. an increasing melting point with increasing pressure). Moreover, ZIF-4 transforms completely to its denser polymorph with **zni** topology (zni_T_ZIF-4) before melting occurs and thus is also expected to show a positive Clapeyron slope behaviour.

We generally find slightly lower specific pore volumes and slightly higher densities for a_g_ZIF-4 and a_g_ZIF-zni compared to the ZIF glasses containing a secondary bulky imidazolate-type linker. This proves the importance of the bulky linker for a less dense packing of the molecular building units in the glass state^15,38,42^, and is also in line with systematic differences of the glasses’ ∆*Q*_FSDP_, suggesting lower MRO in the glasses containing two different linkers. The important structural differences of the glasses with and without the secondary bulky linkers are further corroborated by hydrocarbon sorption experiments, showing that a_g_ZIF-62 and a_g_TIF-4 adsorb *n*-butane at 273 K, while a_g_ZIF-4 and a_g_ZIF-zni do not. Apparently, the presence of the secondary bulky imidazolate-type linkers in a_g_ZIF-62 and a_g_TIF-4 ensures that the glass network is more accessible to larger guests. This is in stark contrast with the porosity features of the corresponding crystalline ZIF phases, where more bulky linkers result in a significant reduction of pore volume and increased steric hindrance for the diffusion of larger molecules.

The present study provides important insights into the porosity of MOF glasses and thus contributes to the understanding of the structure of such glasses. Our findings suggest that conventional porosity design principles, as established for crystalline MOFs and crystalline framework materials in general^1,2,33^, cannot be applied for their glassy state. Rather new design concepts for tuning and adjusting the porosity and sorption selectivity of MOF glasses must be developed. We envision that principles which have already been established for other amorphous porous materials may be adaptable for MOF glasses. An example is porous polymers, such as polymers with intrinsic microporosity (PIMs), where molecular building blocks, which facilitate inefficient packing of the polymer chains, result in enhanced porosity^88–91^. Following this route, we propose that via implementation of larger and asymmetric linkers the porosity of MOF glasses is tuneable far beyond the state of the art, opening the door for the development of much more porous MOF glasses with tailored porosity.

## Methods

### Materials synthesis

The synthesis routes for ZIF-4 (chemical composition (Zn(im)_2_) and ZIF-62 (chemical composition Zn(im)_1.65_(bim)_0.35_) were reproduced as stated in our previous publication^30^. Zn(NO_3_)_2_·6H_2_O (4.0 mmol) and either only imidazole (13.2 mmol; for ZIF-4) or imidazole and benzimidazole (11.55 mmol imidazole, 1.66 mmol benzimidazole; for ZIF-62) were dissolved in 90 mL *N*,*N*-dimethylformamide (DMF). The obtained solution was divided into ten 9 mL portions, which were transferred into ten 12 mL borosilicate vials. The sealed reaction vials were transferred to an oven preheated to 100 °C and kept there for 7 days. After cooling to room temperature, the reaction volumes were recombined, and the formed crystals were filtered off and washed three times with about 20 mL DMF, followed by drying at 200 °C under a dynamic vacuum (*p* ~ 10^–4^ kPa) overnight. ZIF-zni was synthesized after a modified solvothermal synthesis route based on ZIF-4 by replacing the original solvent DMF with ethanol. TIF-4 (chemical composition: (Zn(im)_1.68_(mbim)_0.32_) was synthesized after the same procedure as ZIF-62 by replacing the secondary bulky linker benzimidazole by 5-methylbenzimidazole in the synthesis. Based on the four crystalline materials, their thermal products were obtained via thermal treatment in a TGA/DSC apparatus (see Supplementary Methods 5.2).

### X-ray powder diffraction (XRPD)

XRPD patterns were recorded at room temperature on a Siemens D5005 diffractometer in Bragg–Brentano geometry. Data were collected with CuK_α_ radiation in the range from 5° to 50° 2*θ* with a step size of 0.02°. Finely ground samples (crystalline or glassy) were deposited on a glass holder or a single crystal zero background sample holder made of silicon (cut along the (610) plane). For phase identification, structureless profile fits (Pawley method^64^) were performed with the TOPAS academic v6 software^92^.

### Fourier transform infra-red (FTIR) spectroscopy

Fourier transform infra-red (FTIR) spectroscopy was carried out on a Perkin Elmer SpectrumTwo FTIR spectrometer ($\widetilde{\nu }$ = 400–4000 cm^–1^) in reflection mode using a diamond ATR (attenuated total reflectance) unit. Powdered samples were placed on the diamond ATR unit and carefully compressed with a stamp for the measurement.

### Scanning electron microscopy (SEM)

Scanning electron microscopy (SEM) imaging was performed with a Hitachi S-4500 instrument. For measurements, samples were placed on a conductive adhesive pad. Imaging was done with 1 kV accelerating voltage on a secondary electron detector. All investigated samples were ground and taken from the sorption tubes after the conduction of physisorption measurements before imaging.

### Nuclear magnetic resonance (NMR) spectroscopy

Solution ^1^H NMR spectroscopy was performed on digested crystalline and glassy ZIF samples with Bruker DPX-300, DPX 500 or Agilent DD2 500 spectrometers. The solid samples were digested before the measurement using DMSO-*d*_6_ (0.5 mL) and DCl/D_2_O (35 wt%, one drop, <0.1 mL) as solvents. The data were processed with the MestReNova (v14.2.0) software. Data were referenced to the residual proton signal of DMSO and chemical shifts are given relative to tetramethylsilane.

### Thermal analysis

Differential scanning calorimetry measurements (DSC) were performed on a DSC 25 from TA Instruments under a constant nitrogen flow (50 mL min^–1^). Before the measurement, the samples were ground and placed in a hermitically sealed aluminium crucible and a hole was pinched into the lid of the sealed crucible. Simultaneous thermogravimetric analysis/differential scanning calorimetry (TGA/DSC) measurements were conducted on an STA 504 instrument or SDT 650 from TA Instruments under a constant argon flow (4 L h^–1^) for STA 504 or a nitrogen flow (100 mL min^–1^) for SDT 650 on powdered samples. Data were processed and evaluated using the TRIOS (v5.1.0.46403) software from TA instruments. The melting temperatures (*T*_m_) are determined as the peak offset, the glass transition temperatures (*T*_g_) as the peak onset, whereas all other derived temperatures are defined as the peak temperature. The enthalpies are determined from the integral of the corresponding signal.

### Isothermal gas physisorption

Low-pressure experiments up to 100 kPa were performed with a Quantachrome iQ MP porosimeter. Sample quantities of about 100 mg (for glasses) and at least 50 mg (for all others) were used for the experiments. Prior to the first measurement, the ground samples were degassed under a dynamic vacuum (*p* ≈ 10^–5^ kPa) at 200 °C for 2 h. Gas sorption isotherms were measured with N_2_ (77 K, liquid nitrogen), Ar (87 K, 3P Instruments CryoTune with liquid nitrogen), CO_2_ (195 K, dry ice/*iso*-propanol and 273 K, thermostatic bath) and *n*-butane (273 and 293 K, thermostatic bath), propane and propylene (293 and 313 K, thermostatic bath). Between measurements, samples were degassed under a dynamic vacuum (*p* ≈ 10^–5^ kPa) at ambient temperature for ~3 h. After adsorption measurements with *n*-butane, the samples were again heated to 200 °C for 30 min under a dynamic vacuum (*p* ≈ 10^–5^ kPa). High-pressure CO_2_ adsorption measurements up to 4130 kPa were conducted using a Microtrac Bel Belsorp VC instrument. The sample holder was filled with about 300 mg of pre-evacuated sample (200 °C in dynamic vacuum for 2 h) and installed in the isothermal box of the instrument, which was kept at 298 K. The sample was evacuated prior to the measurement.

### X-ray total scattering

X-ray total scattering data for ZIF-4, a_T_ZIF-4, zni_T_ZIF-4, a_g_ZIF-4, ZIF-zni, a_g_ZIF-zni and a_g_ZIF-62 were collected at beamline I15-1 of Diamond Light Source (DLS, UK) using a monochromatic X-ray beam (*λ* = 0.161669 Å, 76.7 keV). Samples were finely ground before loading into 1.5 mm (outer diameter) borosilicate capillaries. X-ray total scattering data for TIF-4 and a_g_TIF-4 were collected at beamline P02.1 at Deutsches Elektronen-Synchrotron (DESY, Germany) using a monochromatic X-ray beam (*λ* = 0.20734 Å, 60 keV). The samples were placed in a 1 mm (outer diameter) quartz glass capillary. For all datasets, background subtraction was performed with scattering data collected from an empty capillary. Background subtraction, multiple, container and Compton scattering, as well as absorption were done with the GudrunX programme^93^. The normalized reciprocal space data (*S*(*Q*), see Supplementary Fig. 41 for corresponding *Q*_max_ values) were Fourier transformed to yield the X-ray pair distribution functions (XPDFs) in the form of *D*(*r*)^69,94^.

## Supplementary information

Supplementary Information

## Data Availability

The authors declare that all data supporting the findings of this study are available within the article and its supplementary information files. The corresponding raw data are available on request from the corresponding author S.H.
