# Supplementary material for: Quantification of gas-accessible microporosity in metal-organic framework glasses
Source: Nat Commun. 2022 Dec 14;13:7750. doi: 10.1038/s41467-022-35372-5 (PMC9751146; doi:10.1038/s41467-022-35372-5)
Supplement: Supplementary file 1 — Supplementary Information [file 41467_2022_35372_MOESM1_ESM.pdf]

Supplementary Information

Quantification of gas-accessible microporosity in metal-organic framework glasses

Louis Frentzel-Beyme[a], Pascal Kolodzeiski[a], Jan-Benedikt Weiß[a], Andreas Schneemann[b], Sebastian Henke[a*]

[a]Anorganische Chemie, Fakultät für Chemie & Chemische Biologie, Technische Universität Dortmund, Otto-Hahn Straße 6, 44227 Dortmund, Germany

[b]Anorganische Chemie I, Technische Universität Dresden, Bergstrasse 66, 01069 Dresden

Email: sebastian.henke@tu-dortmund.de

Contents

Supplementary Notes 1 - Graphical representations of network topologies 2

Supplementary Methods 2 - X-ray powder diffraction data 3

Supplementary Methods 3 - Fourier-transform infrared (FTIR) spectroscopy data 9

Supplementary Methods 4 - ^{1}H NMR spectroscopy data 10

Supplementary Methods 5 - Thermal analysis 16

Supplementary Methods 5.1 - Differential scanning calorimetry (DSC) 16

Supplementary Methods 5.2 - Simultaneous thermogravimetric analysis / differential scanning calorimetry (TGA/DSC) 18

Supplementary Methods 6 - Photography 22

Supplementary Methods 7 - Scanning electron microscopy 23

Supplementary Methods 8 - X-ray total scattering data 24

Supplementary Methods 8.1 - Fitting of the first sharp diffraction peak (FSDP) 26

Supplementary Methods 9 - Isothermal gas physisorption data 27

Supplementary Methods 9.1 - Summary of adsorption capacities of MOF glasses 36

Supplementary Methods 9.2 - Surface area and pore volume analysis 38

Supplementary Methods 9.3 - Pore size distribution analysis 42

Supplementary Methods 9.4 - Density approximation 43

Supplementary References 44

Supplementary Notes 1 - Graphical representations of network topologies

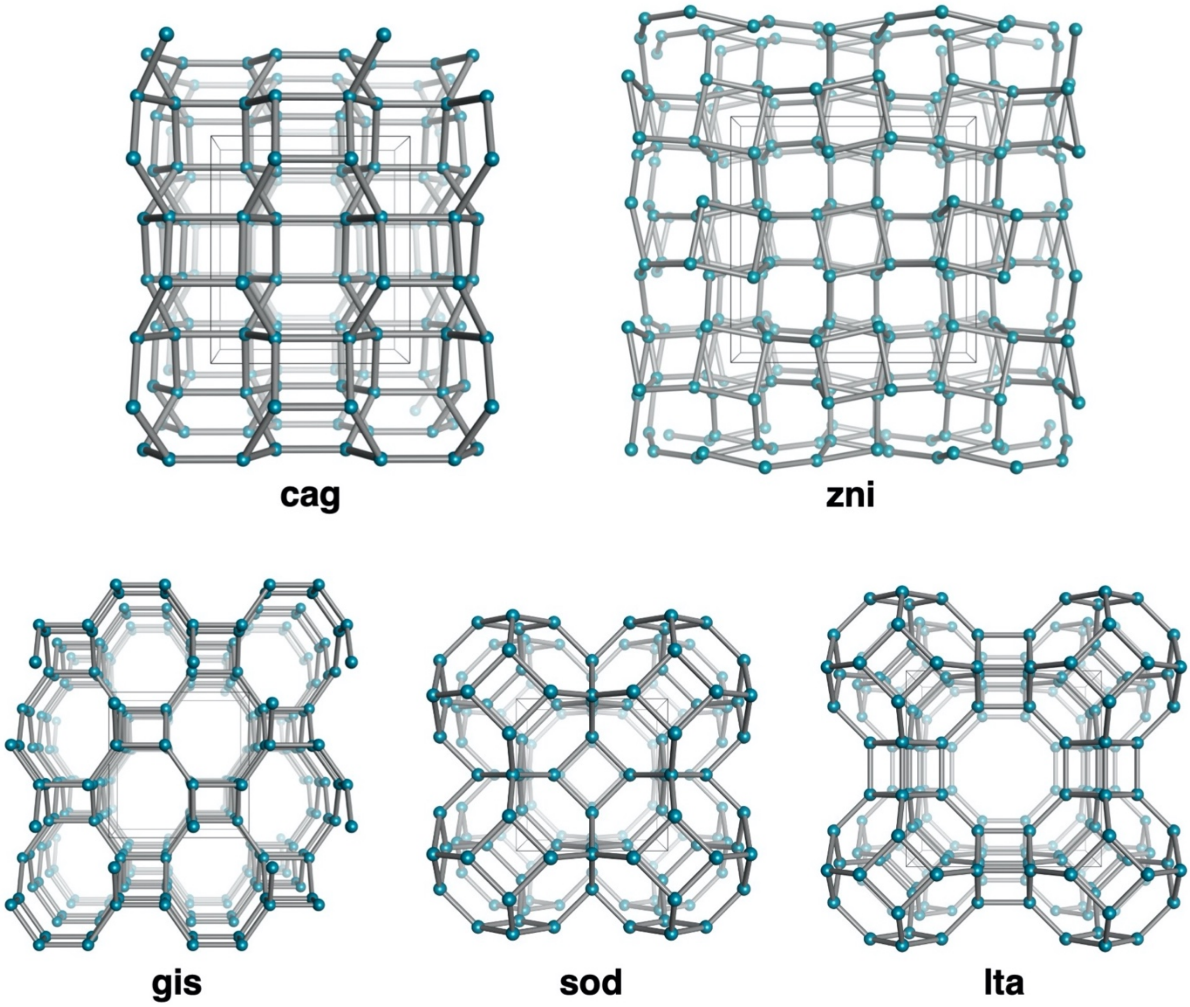

Supplementary Figure 1. Graphical representations of the relevant network topologies under discussion here. The images were created based on the topological data from the *Reticular Chemistry Structure Resource Database* (http://rcsr.anu.edu.au). The cyan spheres represent the positions of the Zn^{2+} ions (i.e. the tetrahedral vertices of the network and the grey sticks represent the positions of the imidazolate-type linkers. Unit cells are shown with black lines. The three letter codes originate from the names of typical framework materials possessing these topologies: **cag**, $CaGa_2O_4$; **zni**, zinc imidazolate; **gis**, gismondine; **sod**, sodalite; **lta**, Linde type A zeolite.

Supplementary Methods 2 - X-ray powder diffraction data

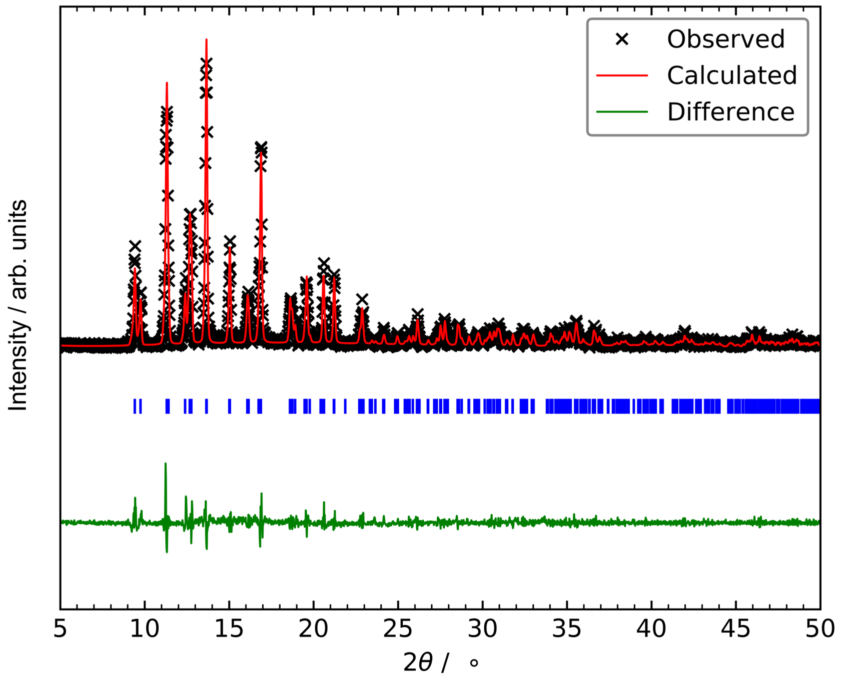

Supplementary Figure 2. Profile fit (Pawley method) to the XRPD pattern of ZIF-4. The blue tick marks indicate the positions of allowed Bragg peaks.

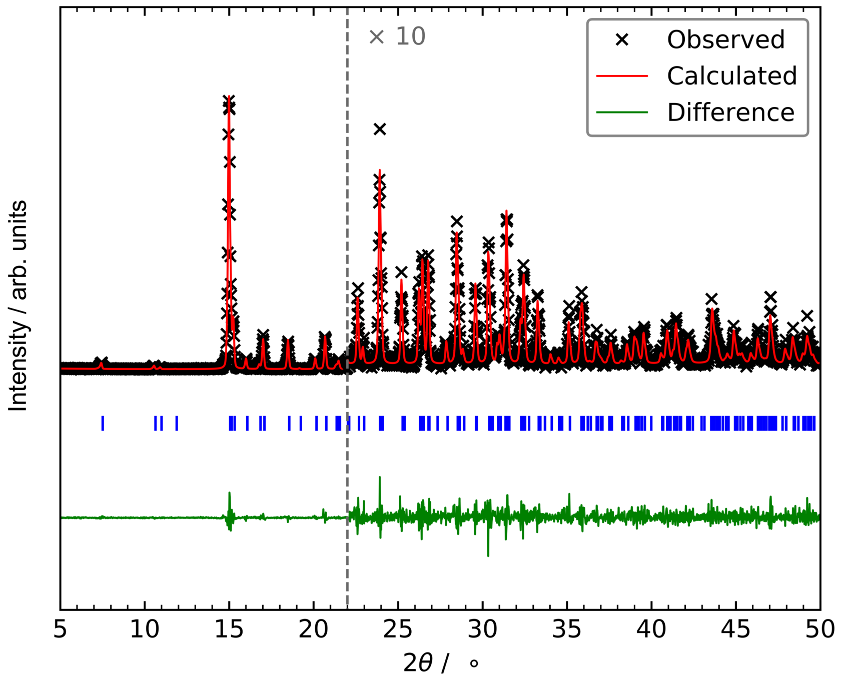

Supplementary Figure 3. Profile fit (Pawley method) to the XRPD pattern of ZIF-zni. The blue tick marks indicate the positions of allowed Bragg peaks.

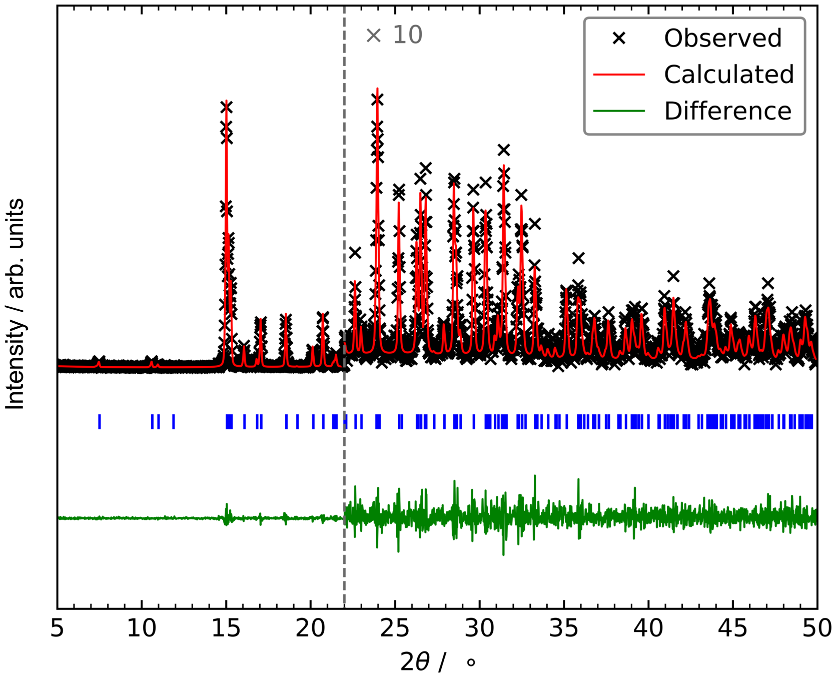

Supplementary Figure 4. Profile fit (Pawley method) to the XRPD pattern of zni$_{\mathrm{T}}$ZIF-4. The blue tick marks indicate the positions of allowed Bragg peaks.

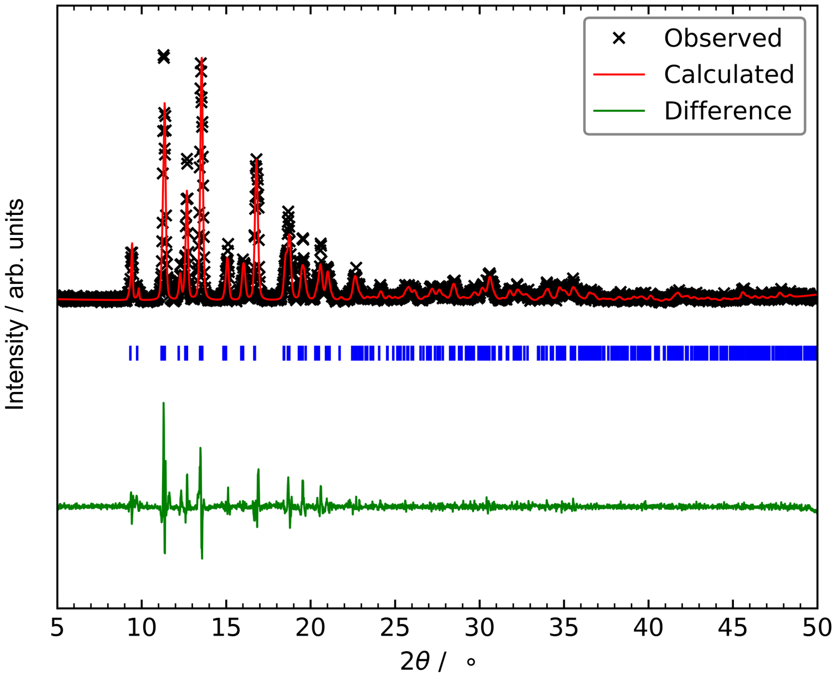

Supplementary Figure 5. Profile fit (Pawley method) to the XRPD pattern of ZIF-62. The blue tick marks indicate the positions of allowed Bragg peaks.

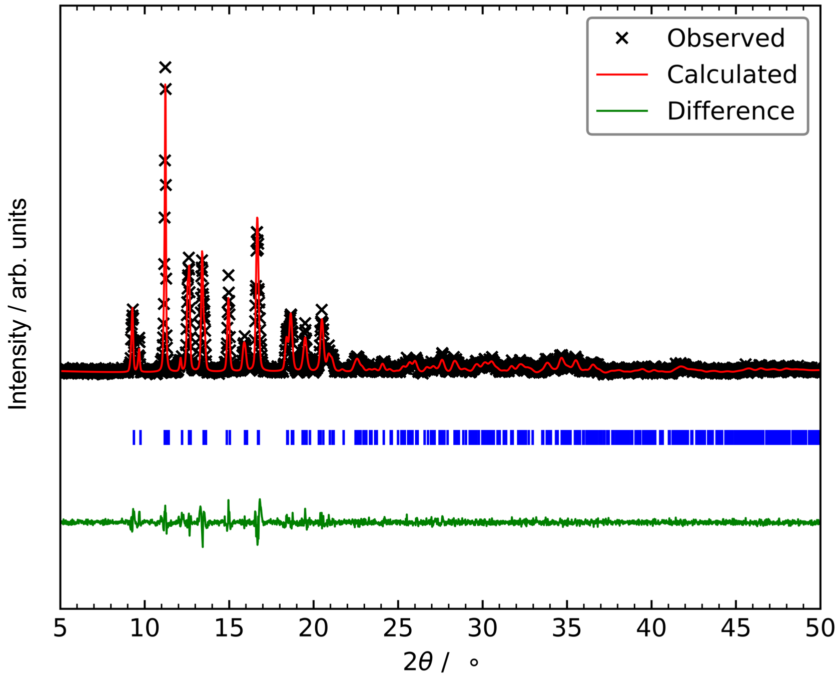

Supplementary Figure 6. Profile fit (Pawley method) to the XRPD pattern of TIF-4. The blue tick marks indicate the positions of allowed Bragg peaks.

Supplementary Table 1. Unit cell parameters and corresponding R_{wp}, R_{exp} and χ values determined by the above displayed structureless profile fits (Pawley method).

compound	ZIF-4	zni_TZIF-4	ZIF-zni	ZIF-62	TIF-4
crystal system	orthorhombic	tetragonal	tetragonal	orthorhombic	orthorhombic
space group	$Pbca$	$I4_1cd$	$I4_1cd$	$Pbca$	$Pbca$
a / Å	15.488(3)	23.522(2)	23.499(2)	15.548(10)	15.483(7)
b / Å	15.528(3)	23.522(2)	23.499(2)	15.819(11)	15.789(8)
c /Å	18.113(4)	12.4512(11)	12.4691(12)	18.164(16)	18.113(10)
α / °	90	90	90	90	90
β / °	90	90	90	90	90
γ / °	90	90	90	90	90
V / Å^3	4356.1(16)	6889.1(14)	6885.6(16)	4468(6)	4431(4)
R_{wp} / %	20.33	21.98	20.09	24.18	21.15
R_{exp} / %	15.72	19.42	16.57	16.92	16.81
χ	1.29	1.13	1.21	1.43	1.26

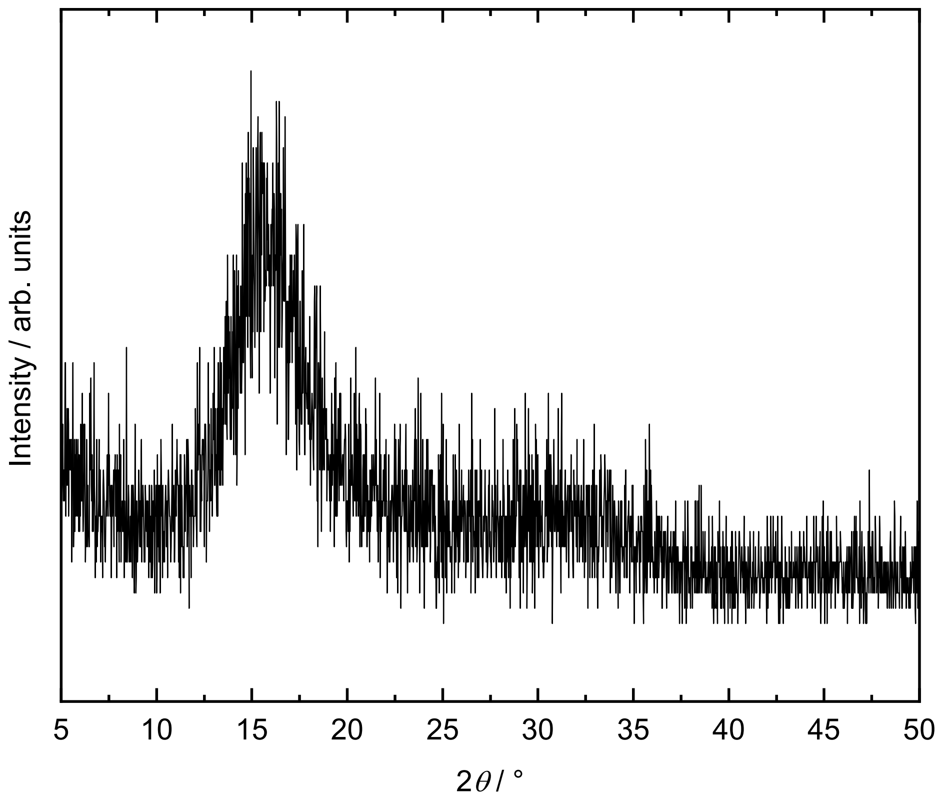

Supplementary Figure 7. XRPD pattern of a_TZIF-4.

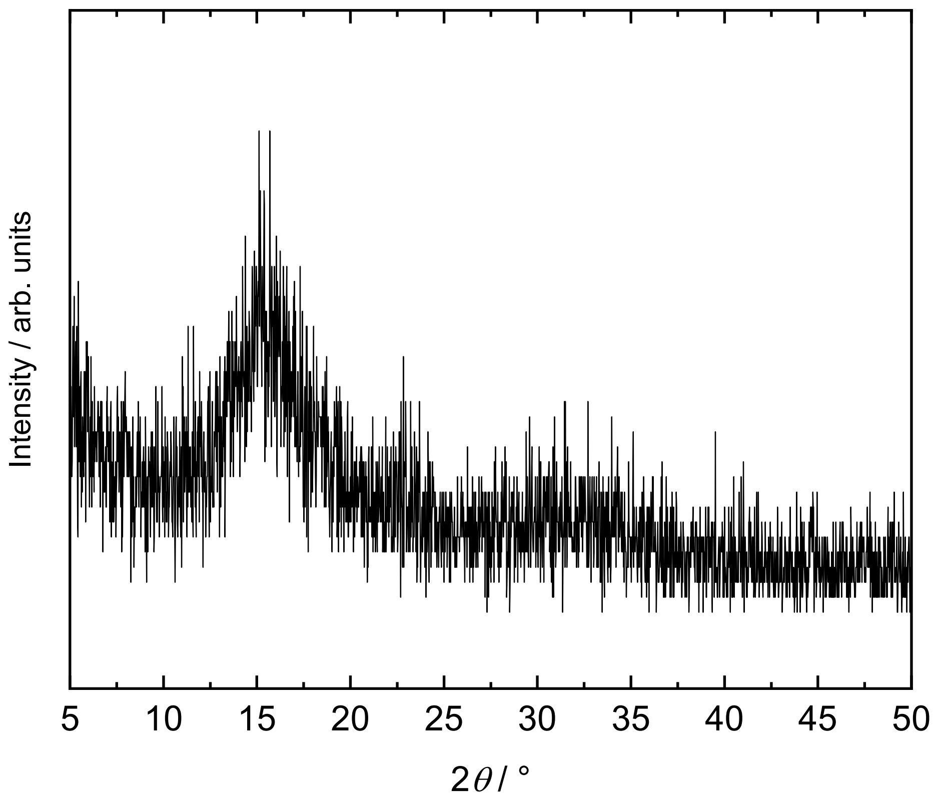

Supplementary Figure 8. XRPD pattern of a_gZIF-4.

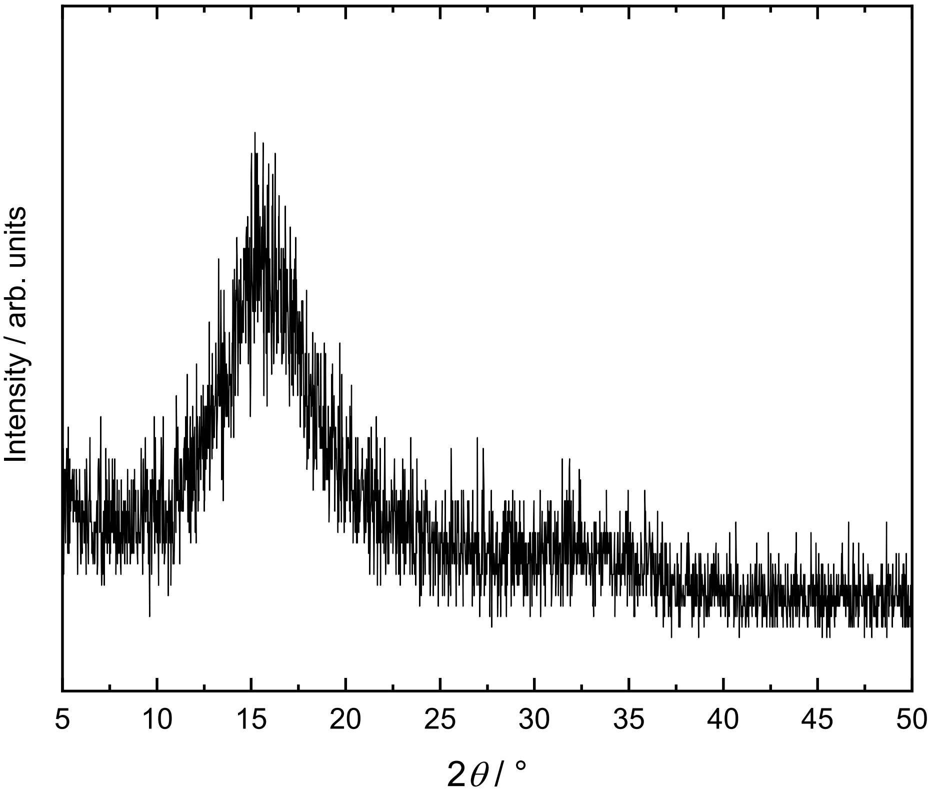

Supplementary Figure 9. XRPD pattern of a_gZIF-zni.

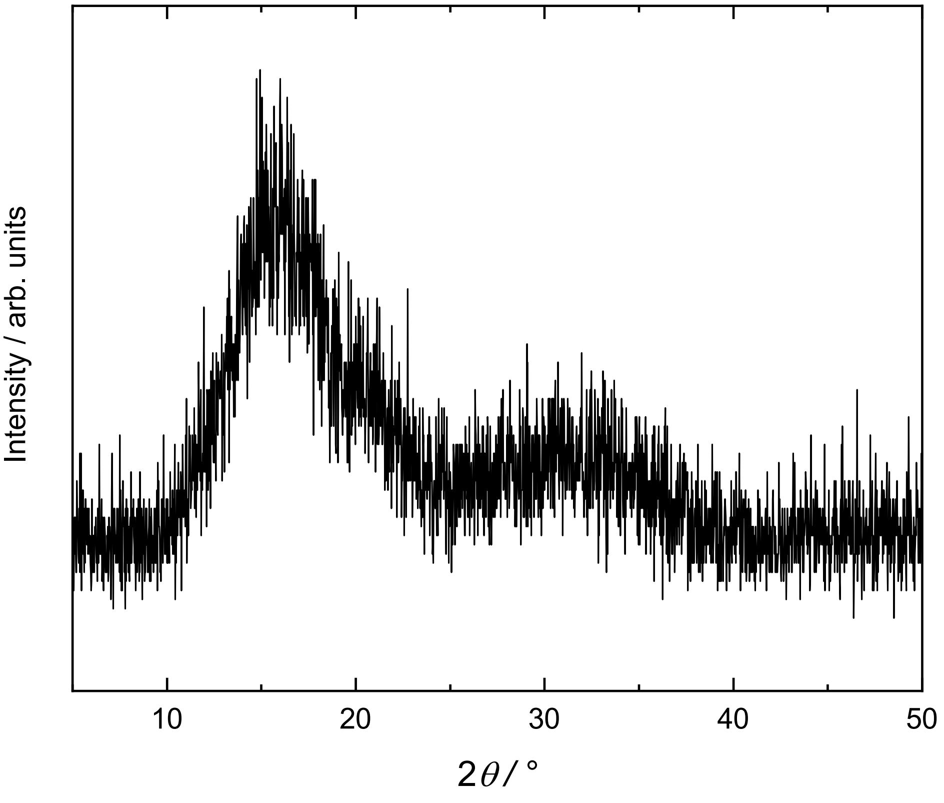

Supplementary Figure 10. XRPD pattern of a_gZIF-62.

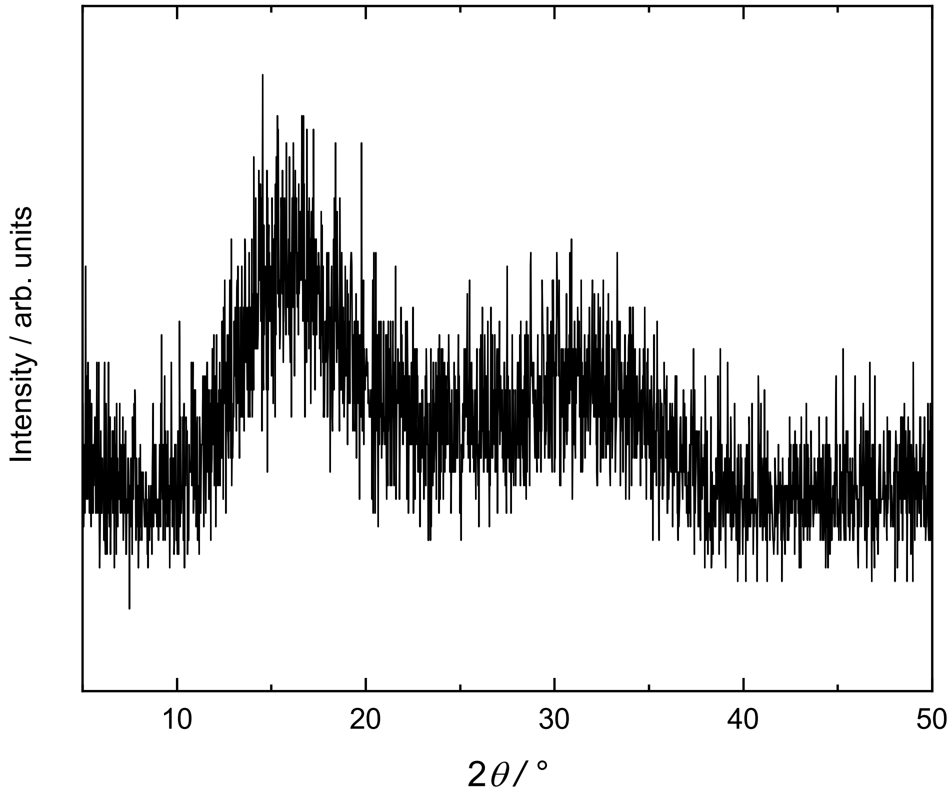

Supplementary Figure 11. XRPD pattern of a_gTIF-4.

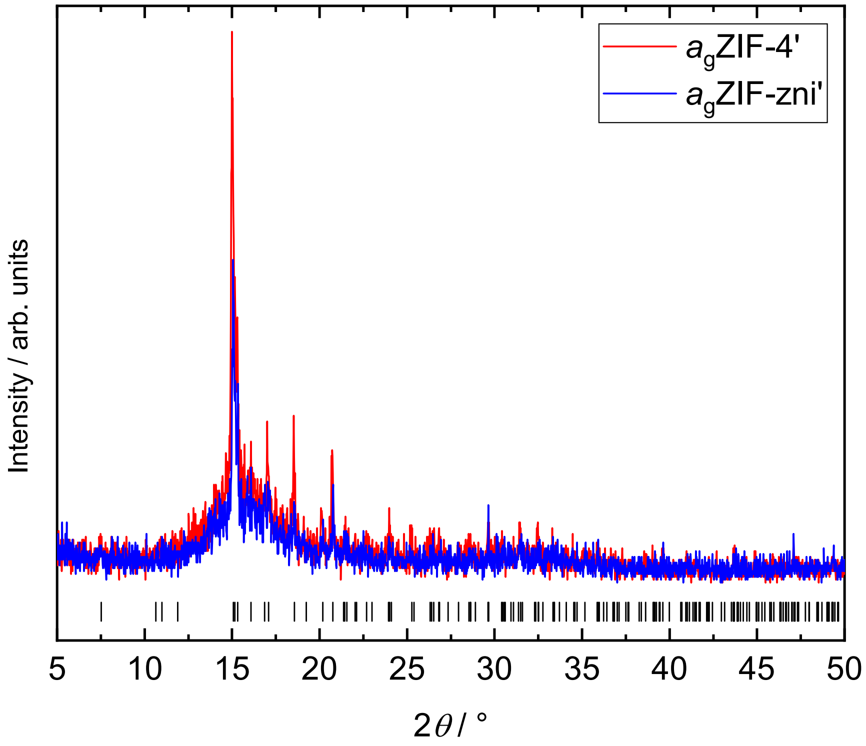

Supplementary Figure 12. Overlay of the XRPD patterns of a_gZIF-zni' and a_gZIF-4' which were obtained by attempting to prepare the desired glasses without an isothermal segment in the TGA/DSC program (see Section S5.2). The black tick marks indicate the allowed Bragg peak positions for ZIF-zni (CCDC code IMIDZB**)**.

Supplementary Methods 3 - Fourier-transform infrared (FTIR) spectroscopy data

The activation of all materials is demonstrated by the absence of the carbonyl stretching band of DMF at 1675 cm^{-1}. DMF is found in the as-synthesized materials as the template for the porous channels (except for ZIF-zni).[1,2]
For ZIF-4 and the amorphous phases derived thereof, a broadening of the vibrational band at 835 cm^{-1} ascribed to the out-of-plane ring deformation of the imidazolate linker[3] is observed (see Supplementary Figure 13). For the **zni** phases (ZIF-zni and zni$_T$ZIF-4) a much sharper band is observed which may be caused by the higher density of the material corresponding to fewer degrees of freedom for this vibration.

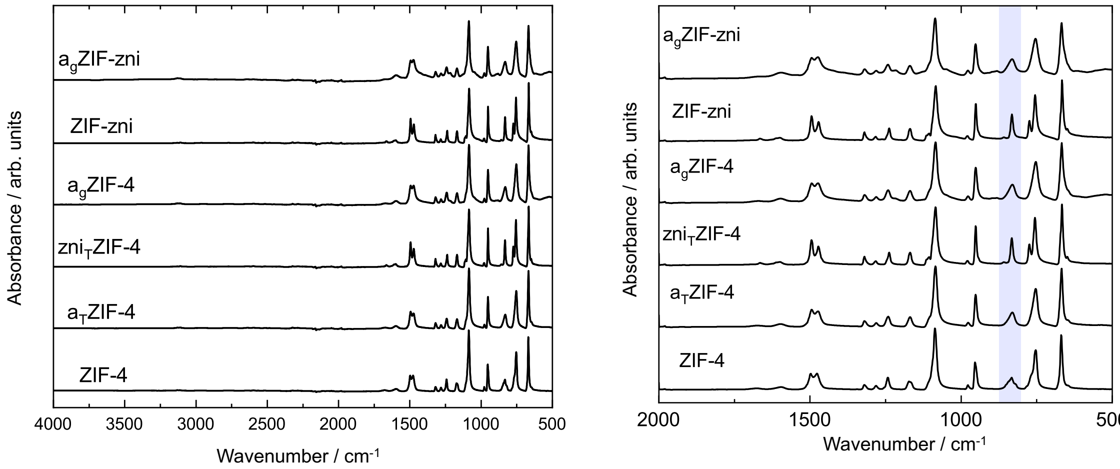

Supplementary Figure 13. Left**:** Stacked plot of the FTIR spectroscopy data of ZIF-4 and ZIF-zni and their thermal products. Right: Zoom in the region from 2000-500 cm^{-1}. The discussed bands at 835 cm^{-1} are highlighted in light blue.

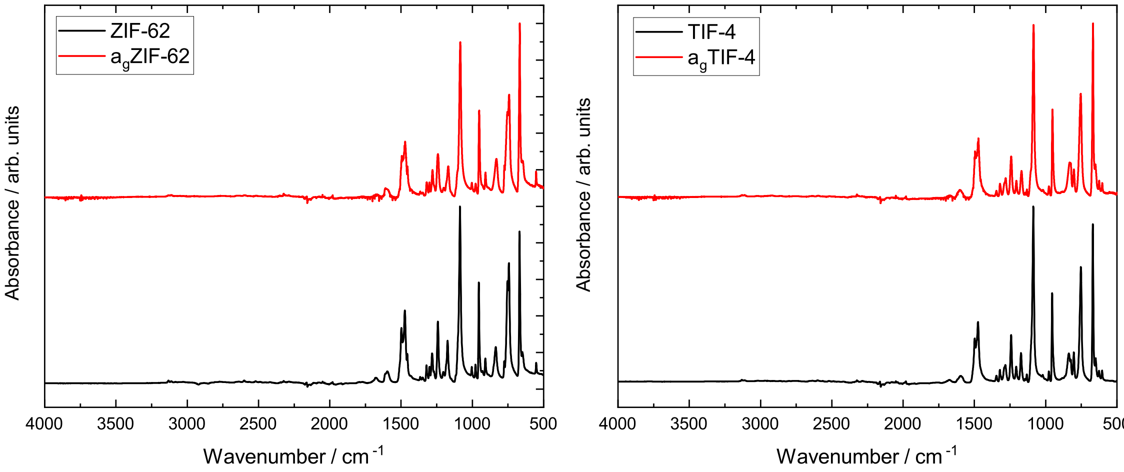

Supplementary Figure 14. Left: FTIR spectroscopy data of ZIF-62 and a$_g$ZIF-62. Right: FTIR spectroscopy data of TIF-4 and a$_g$TIF-4.

Supplementary Methods 4 - ^{1}H NMR spectroscopy data

The activation of all materials is demonstrated by the absence of signals ascribed to DMF.[1,2]

For TIF-4, ZIF-62 and their corresponding glasses the ratio between the implemented linkers has been determined by the integral corresponding to the proton attached to the carbon atom between the two nitrogen atoms in the imidazolate type linkers. The corresponding signals are integrated in the spectra below.

A deeper inspection of the ^{1}H NMR spectroscopy data unveiled additional small signals in the range of 9.0 ppm – 9.5 ppm and 7.5 ppm – 8.5 ppm for a_gZIF-4 and a_gZIF-zni which are ascribed to aromatic or polyaromatic compounds formed as the result of partial framework decomposition during high temperature treatment under inert atmosphere (see Figure 2c for a zoom into the aromatic region). For a_gZIF-4 this has been already observed in the literature.[4] However, as shown by the intensity of the ^{13}C satellite signals assigned to the protons of imidazole, the amount of decomposition is rather low.

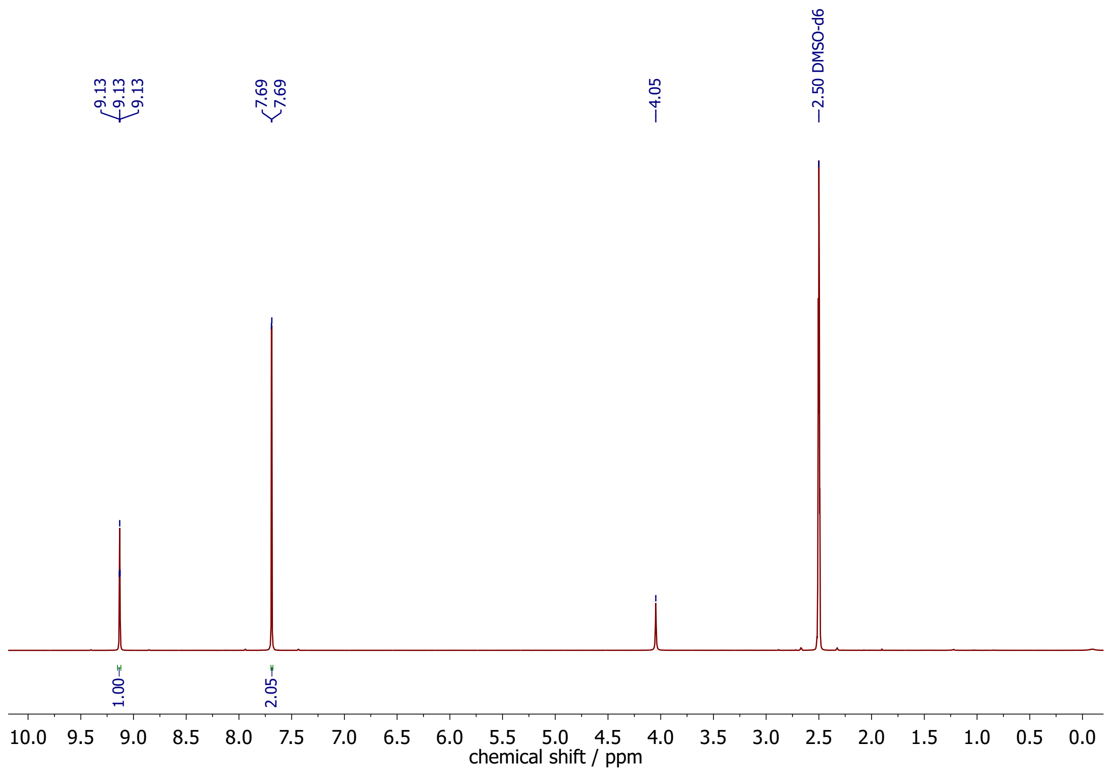

Supplementary Figure 15. ^{1}H NMR spectroscopy data of ZIF-4 recorded in a solvent mixture of DMSO-d_6 and DCl/D_2O at room temperature.

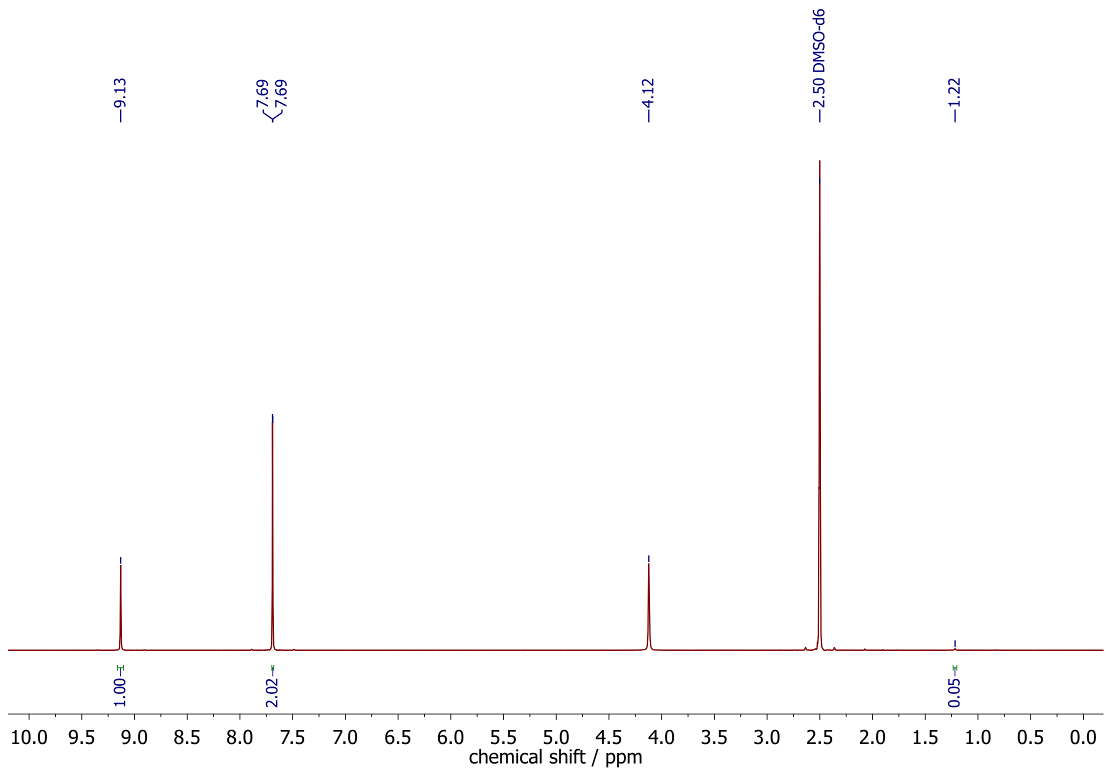

Supplementary Figure 16. ^{1}H NMR spectroscopy data of a$_T$ZIF-4 recorded in a solvent mixture of DMSO-d_6 and DCl/D_2O at room temperature.

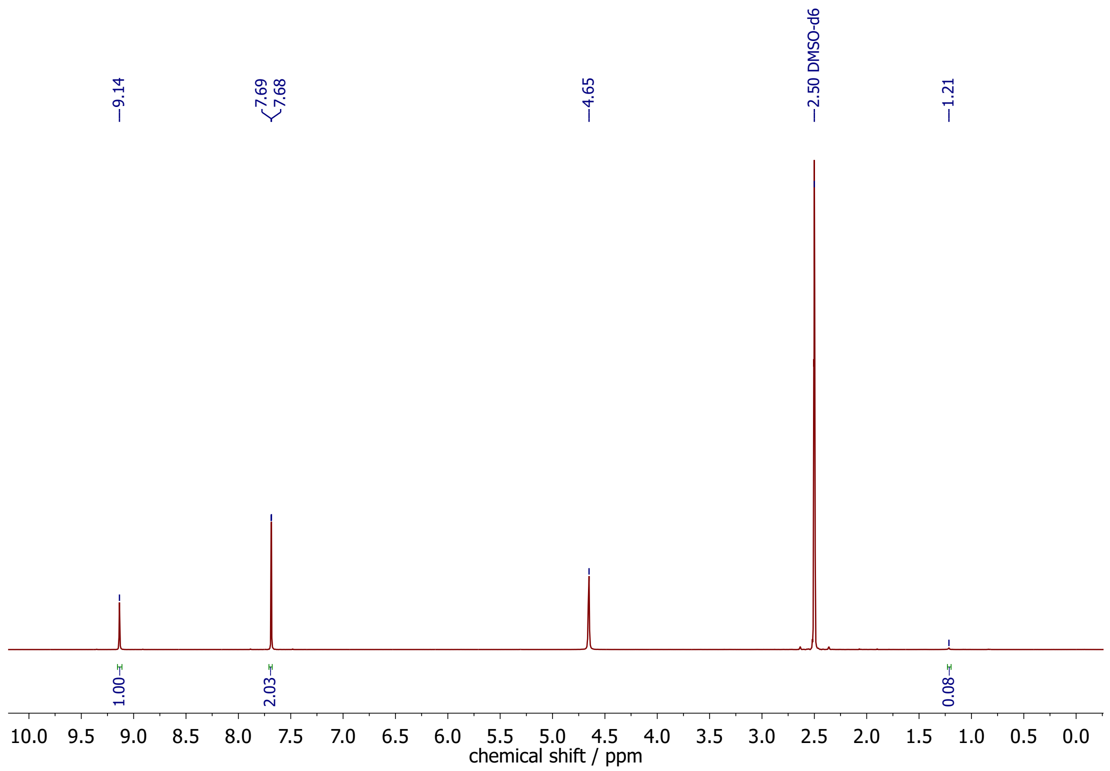

Supplementary Figure 17. ^{1}H NMR spectroscopy data of zni$_T$ZIF-4 recorded in a solvent mixture of DMSO-d_6 and DCl/D_2O at room temperature.

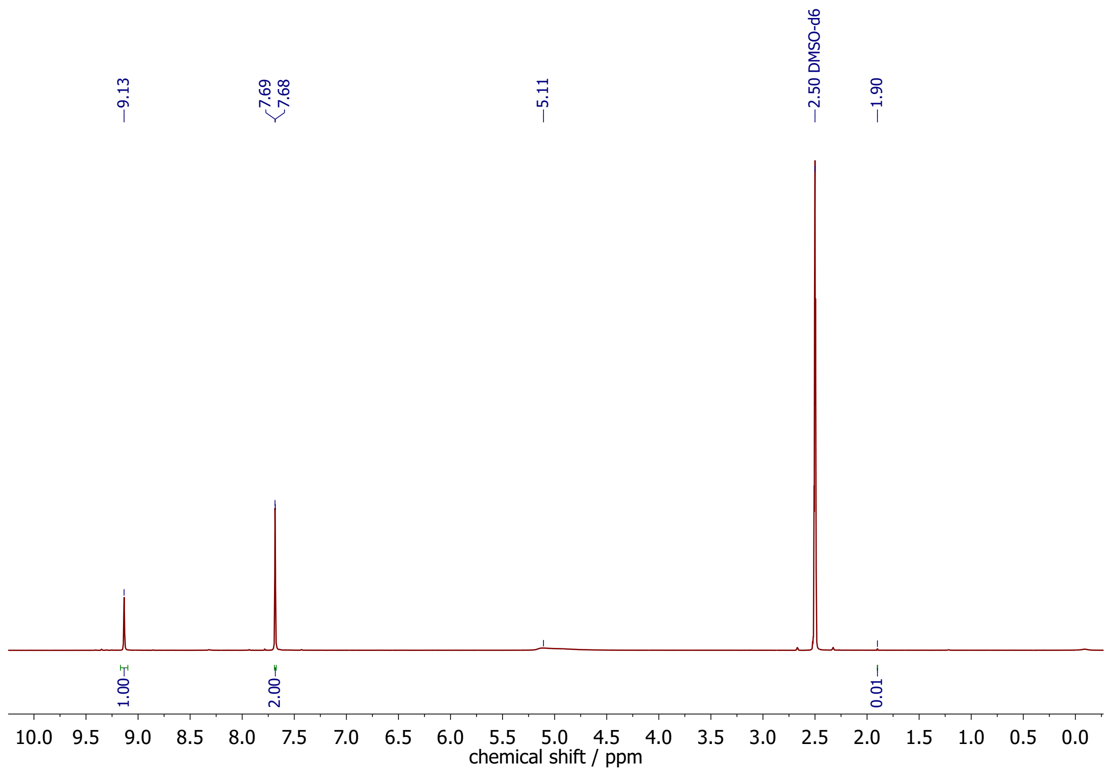

Supplementary Figure 18. ^{1}H NMR spectroscopy data of a_gZIF-4 recorded in a solvent mixture of DMSO-d_6 and DCl/D_2O at room temperature.

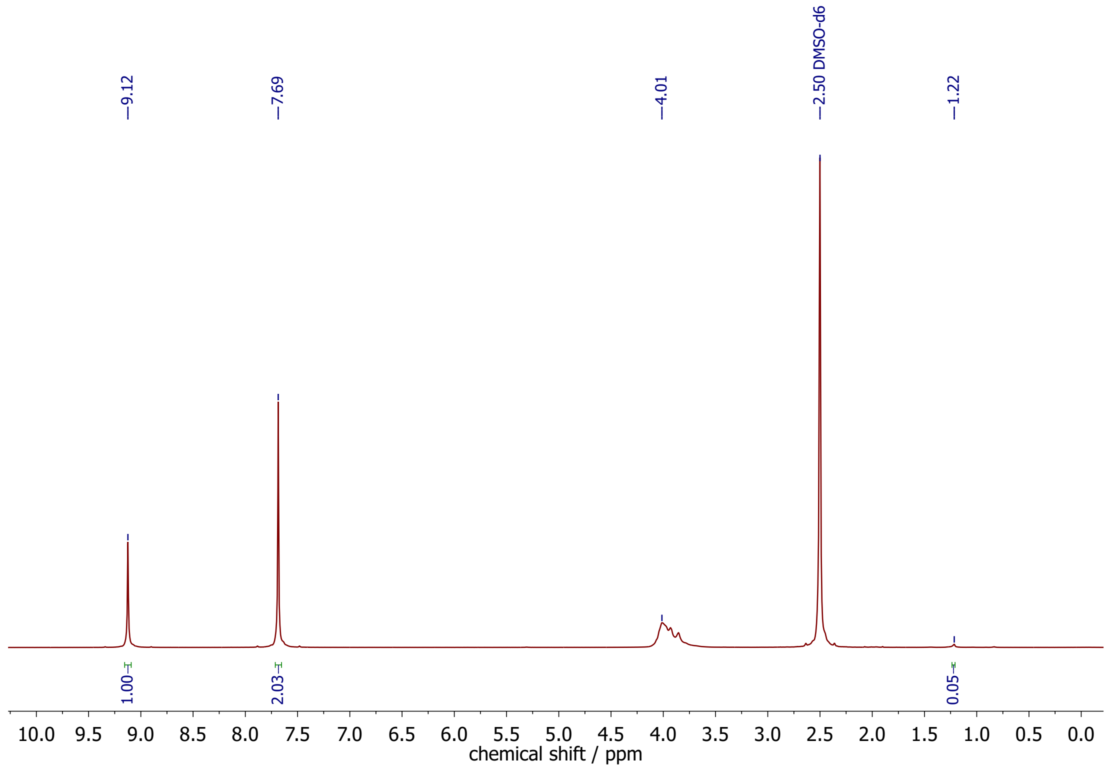

Supplementary Figure 19. ^{1}H NMR spectroscopy data of ZIF-zni recorded in a solvent mixture of DMSO-d_6 and DCl/D_2O at room temperature.

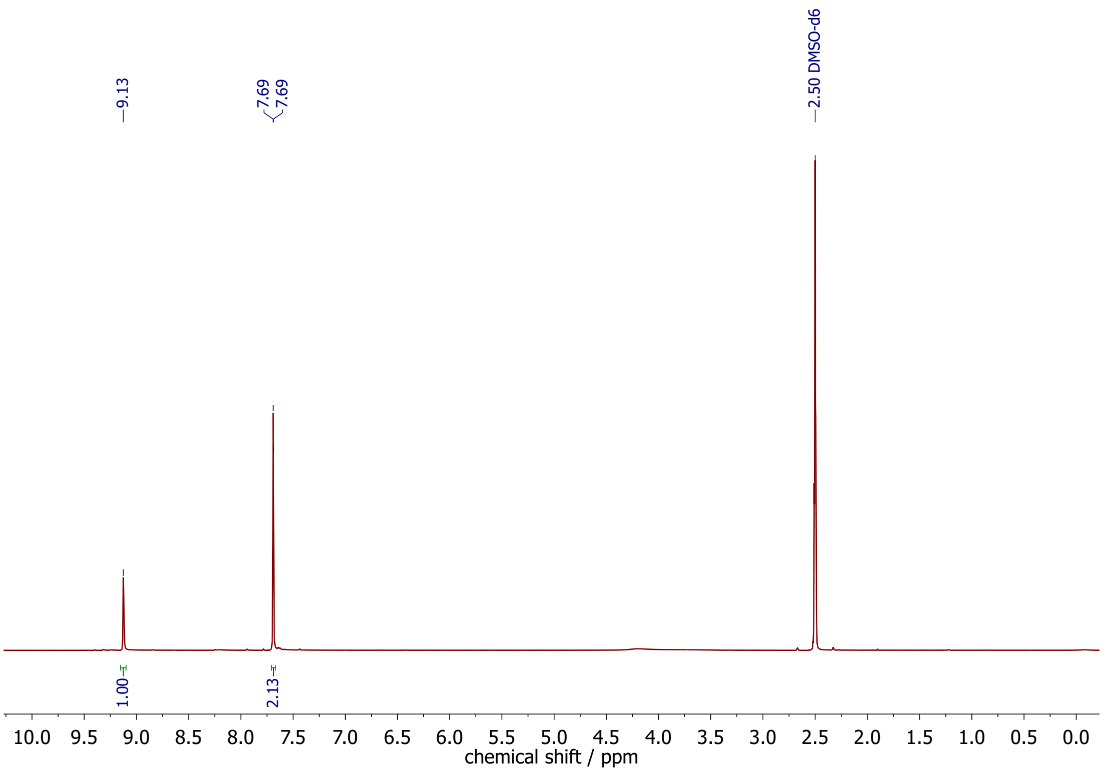

Supplementary Figure 20. ^{1}H NMR spectroscopy data of a_gZIF-zni recorded in a solvent mixture of DMSO-d_6 and DCl/D_2O at room temperature.

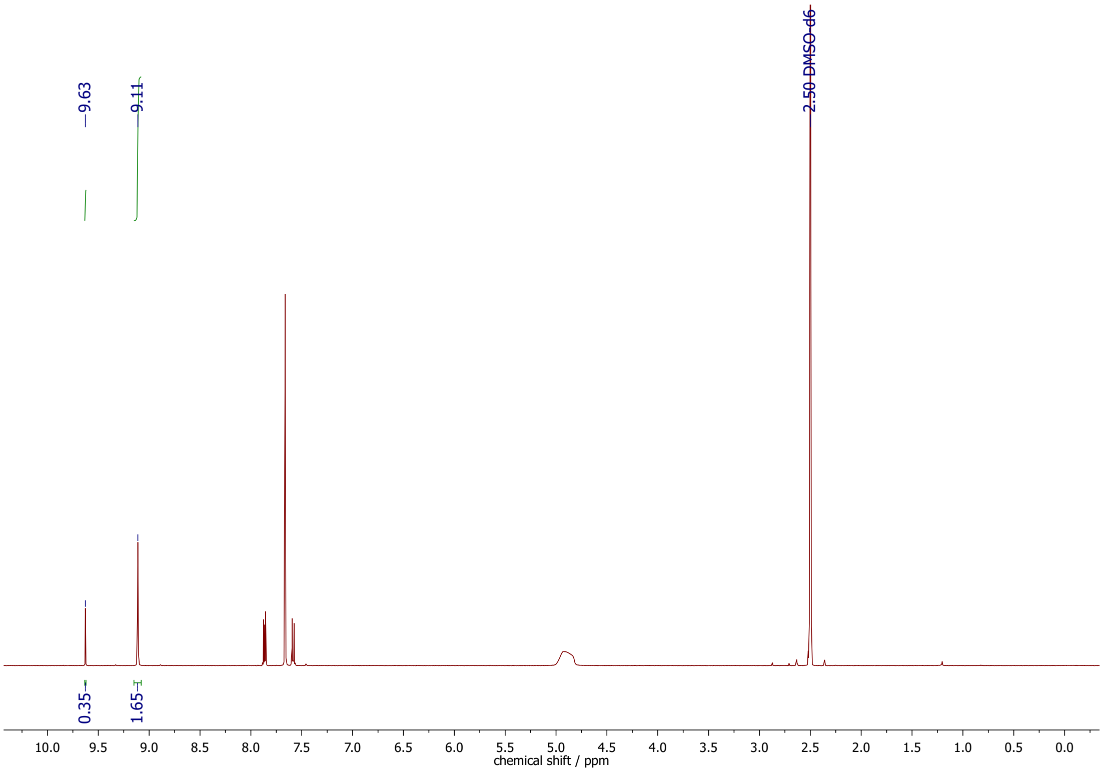

Supplementary Figure 21. ^{1}H NMR spectroscopy data of ZIF-62 recorded in a solvent mixture of DMSO-d_6 and DCl/D_2O at room temperature.

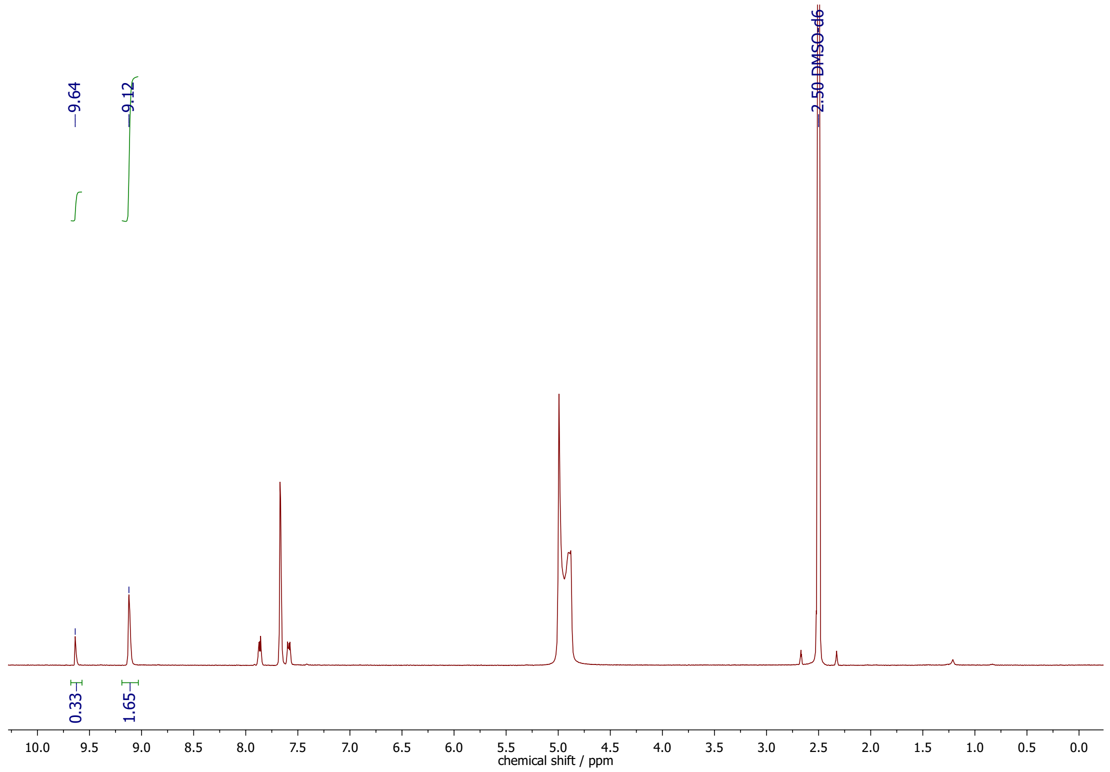

Supplementary Figure 22. ^{1}H NMR spectroscopy data of a$_g$ZIF-62 recorded in a solvent mixture of DMSO-d_6 and DCl/D_2O at room temperature.

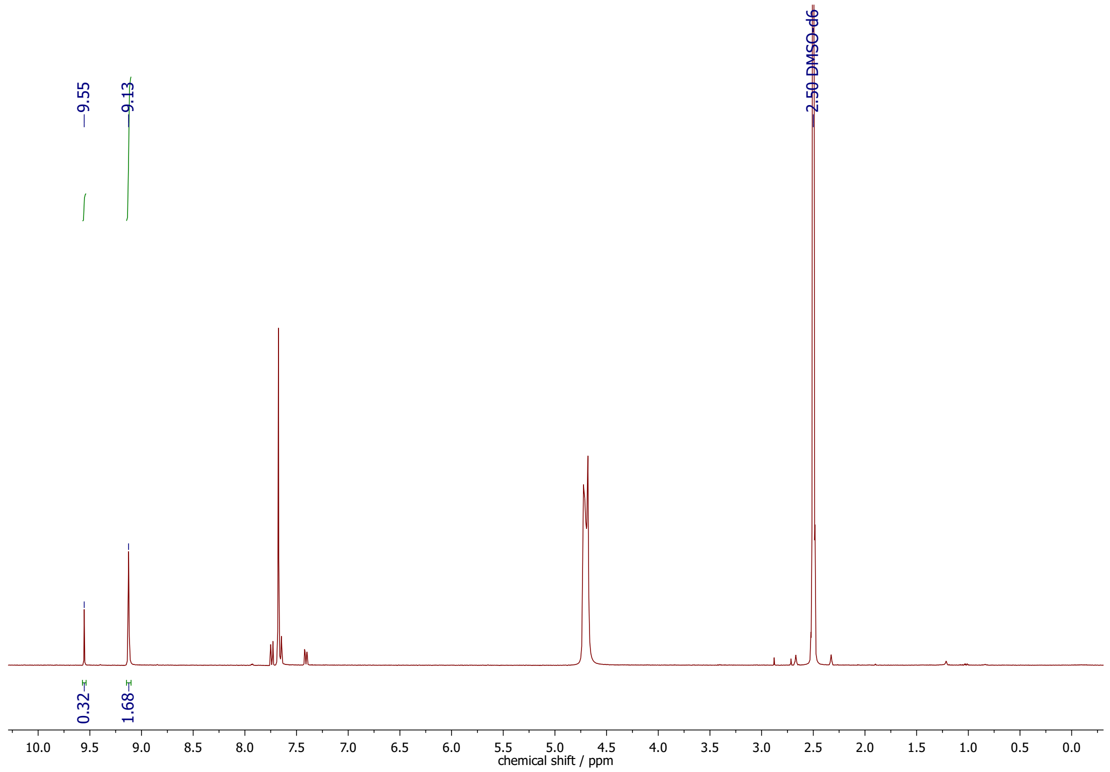

Supplementary Figure 23. ^{1}H NMR spectroscopy data of TIF-4 recorded in a solvent mixture of DMSO-d_6 and DCl/D_2O at room temperature.

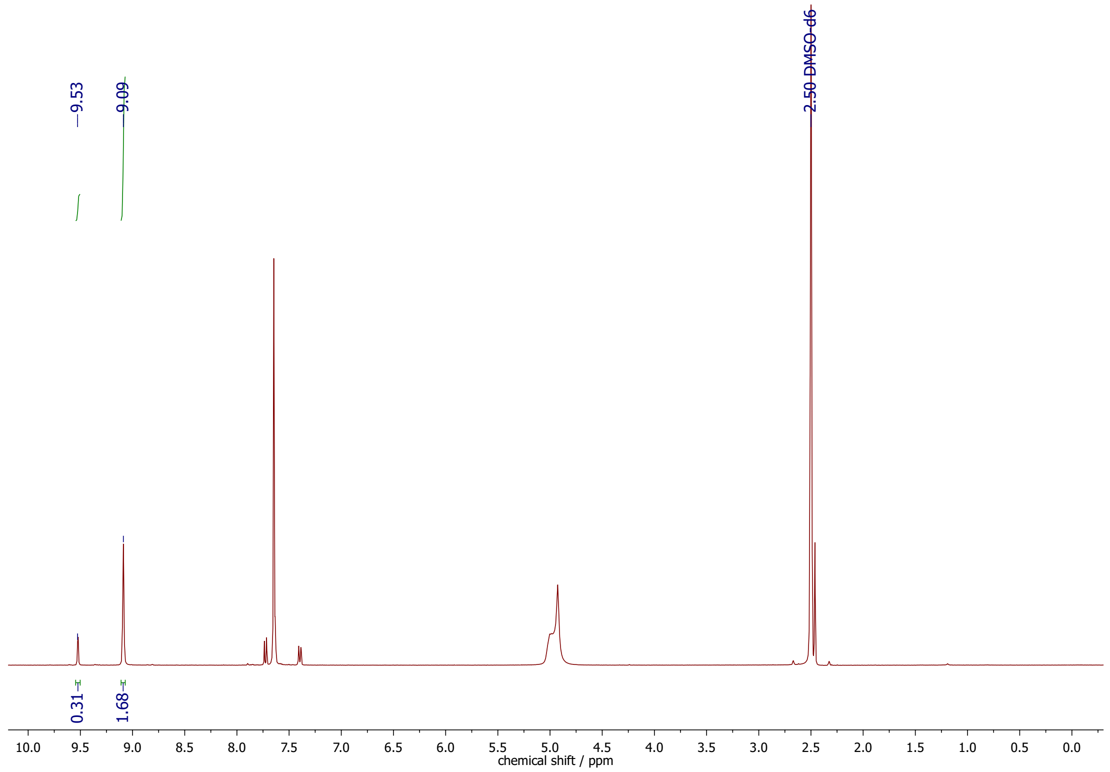

Supplementary Figure 24. ^{1}H NMR spectroscopy data of a$_g$TIF-4 recorded in a solvent mixture of DMSO-d_6 and DCl/D_2O at room temperature.

Supplementary Methods 5 - Thermal analysis

Supplementary Methods 5.1 - Differential scanning calorimetry (DSC)

For all DSC measurements a heating rate of +10 °C min^{-1} was applied. Samples of ZIF-4 and ZIF-zni were heated to a maximum temperature of 600 °C. Samples of ZIF-62, TIF-4 and their corresponding glasses were heated to a maximum temperature of 485 °C.
Data analysis was performed with the TRIOS (v5.1.0.46403) software from TA Instruments. The melting temperatures (T_m) are determined as the peak offset, the glass transition temperatures (T_g) as the peak onset, whereas all other derived temperatures are defined as the peak temperature. The enthalpies are determined from the integral of the corresponding signal.

Supplementary Table 2. Summary of the phase transition temperature determined from the DSC data.

Material	T_{am}	T_{rc}	T_m	T_g
ZIF-4	336 °C	463 °C	579 °C	-
ZIF-zni	-	-	585 °C	-
ZIF-62	-	-	438 °C	331 °C
TIF-4	-	-	436 °C	338 °C

Supplementary Table 3. Summary of the phase transition enthalpies of ZIF-4 and ZIF-zni determined from the DSC data.

Material	ΔH_{am}	ΔH_{rc}	ΔH_{melt}
ZIF-4	–0.28 kJ mol^{-1}	–10.60 kJ mol^{-1}	11.46 kJ mol^{-1}
ZIF-zni	-	-	11.88 kJ mol^{-1}
ZIF-62			5.08 kJ mol^{-1}
TIF-4			2.69 kJ mol^{-1}

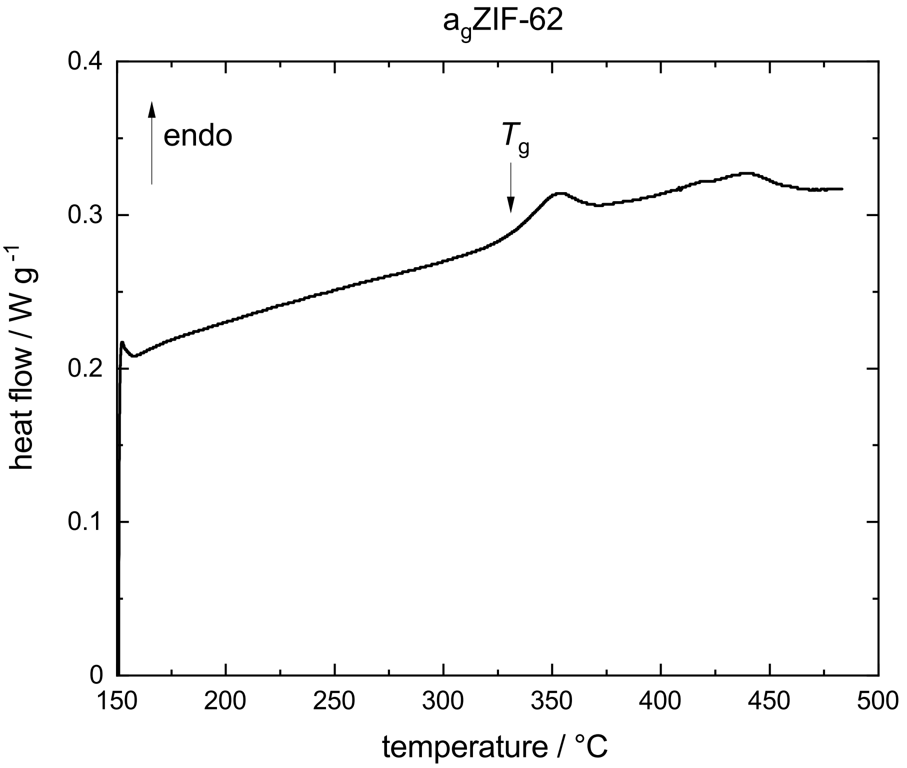

Supplementary Figure 25. DSC data for a_gZIF-62 showing a glass transition.

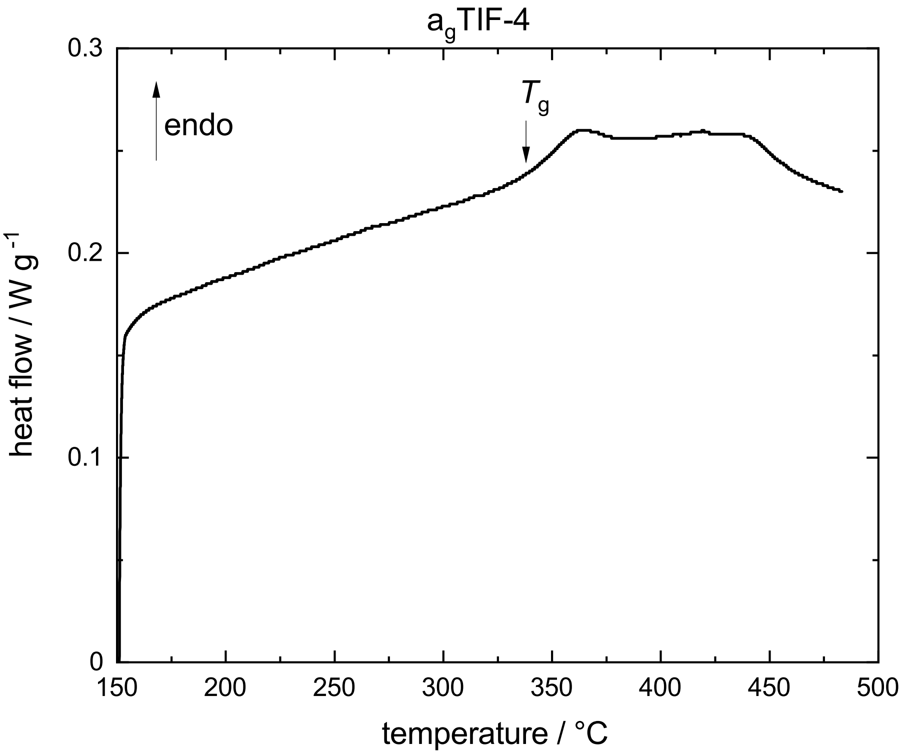

Supplementary Figure 26. DSC data for a_gTIF-4 showing a glass transition.

Supplementary Methods 5.2 - Simultaneous thermogravimetric analysis / differential scanning calorimetry (TGA/DSC)

Several different temperature programs were utilized for the preparation of the thermal products of the investigated ZIFs starting with material of the solvothermally synthesized corresponding crystalline precursor (see Supplementary Table 4).

Supplementary Table 4. Summary of the temperature programs applied for the materials' preparation via TGA/DSC experiments.

material	temperature program
a_TZIF-4	RT (+10 °C min^{-1}) → 379 °C (–10 °C min^{-1}) → RT
zni$_T$ZIF-4	RT (+10 °C min^{-1}) → 546 °C (–10 °C min^{-1}) → RT
a_gZIF-4	RT (+10 °C min^{-1}) → 578 °C –10 min → 578 °C (–10 °C min^{-1}) → 50 °C →RT
a_gZIF-zni	RT (+10 °C min^{-1}) → 578 °C –10 min → 578 °C (–10 °C min^{-1}) → 50 °C →RT
a_gZIF-62	RT (+10 °C min^{-1}) → 485 °C (–10 °C min^{-1}) → RT
a_gTIF-4	RT (+10 °C min^{-1}) → 485 °C (–10 °C min^{-1}) → RT

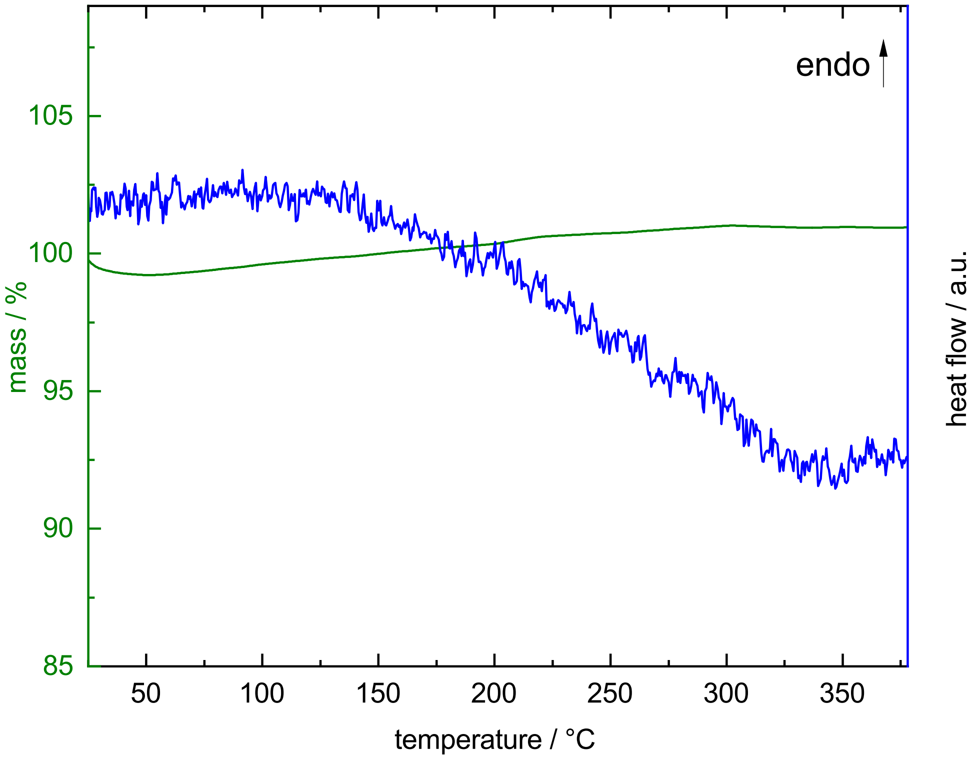

Supplementary Figure 27. TGA/DSC data for the preparation of a_TZIF-4. The cooling process (rate –10 K min^{-1}) was performed without data collection.

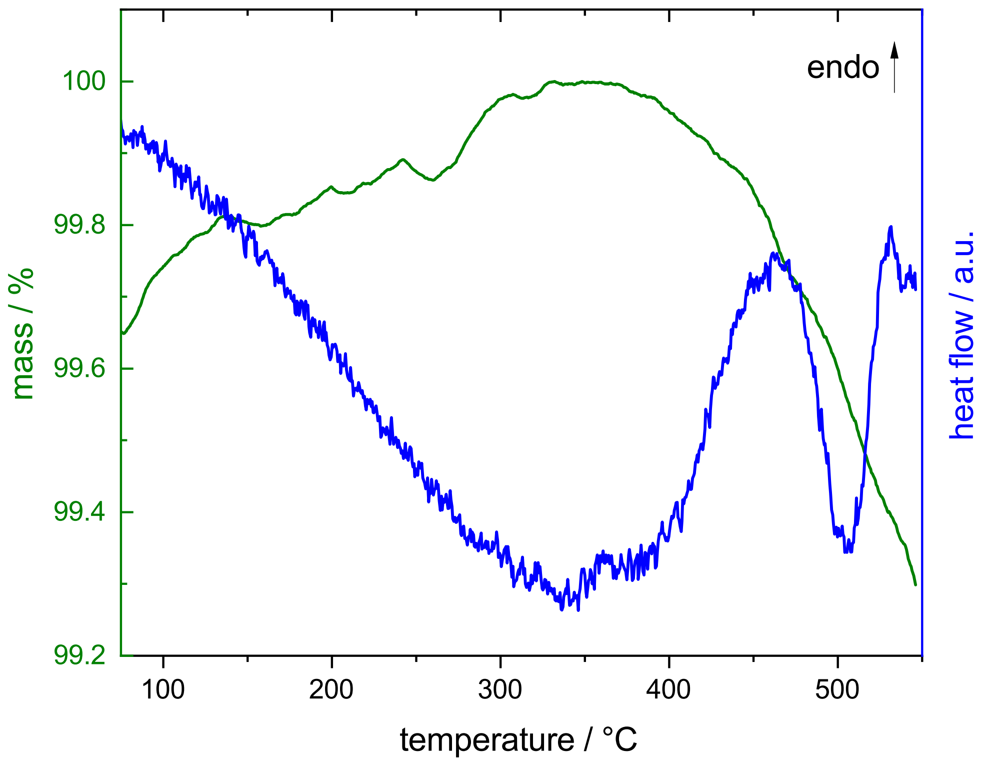

Supplementary Figure 28. TGA/DSC data for the preparation of zni$_T$ZIF-4. The cooling process (rate –10 K min^{-1}) was performed without data collection.

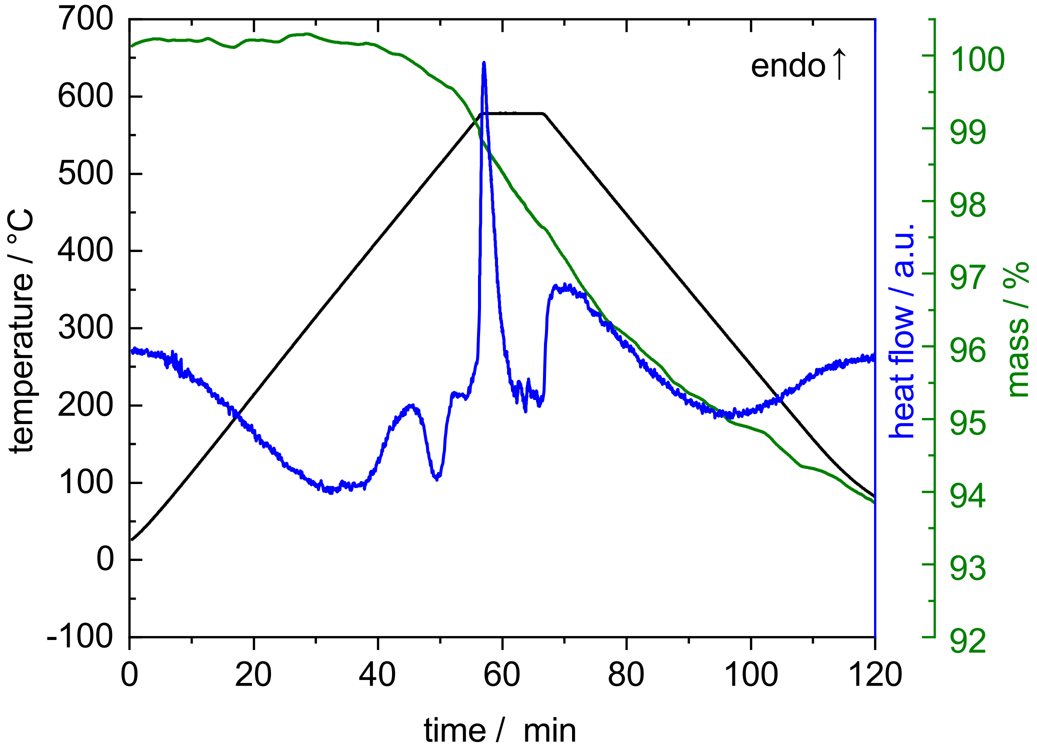

Supplementary Figure 29. TGA/DSC data for the preparation of a$_g$ZIF-4.

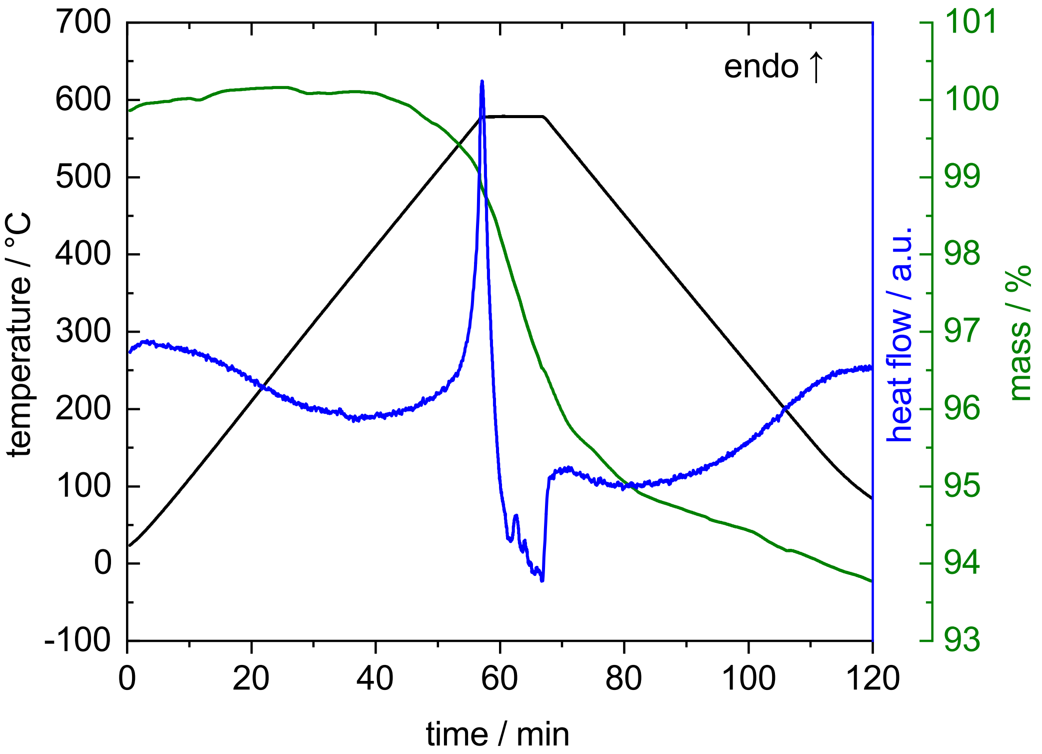

Supplementary Figure 30. TGA/DSC data for the preparation of a_gZIF-zni.

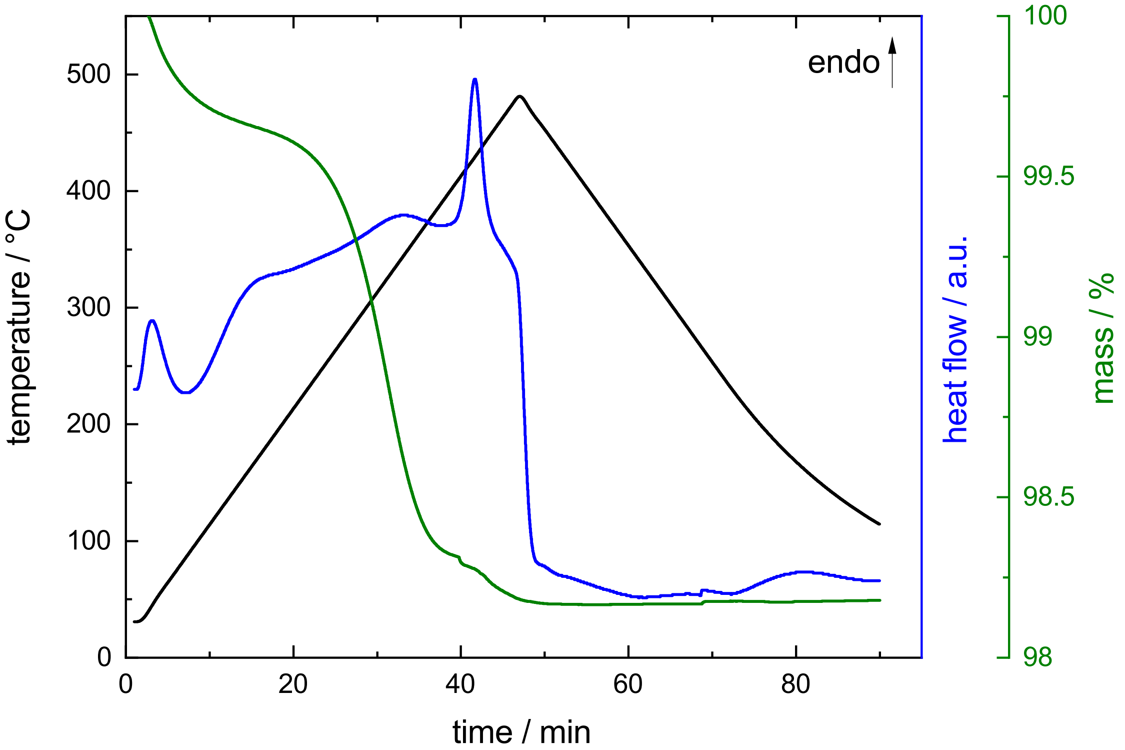

Supplementary Figure 31. TGA/DSC data for the preparation of a_gZIF-62.

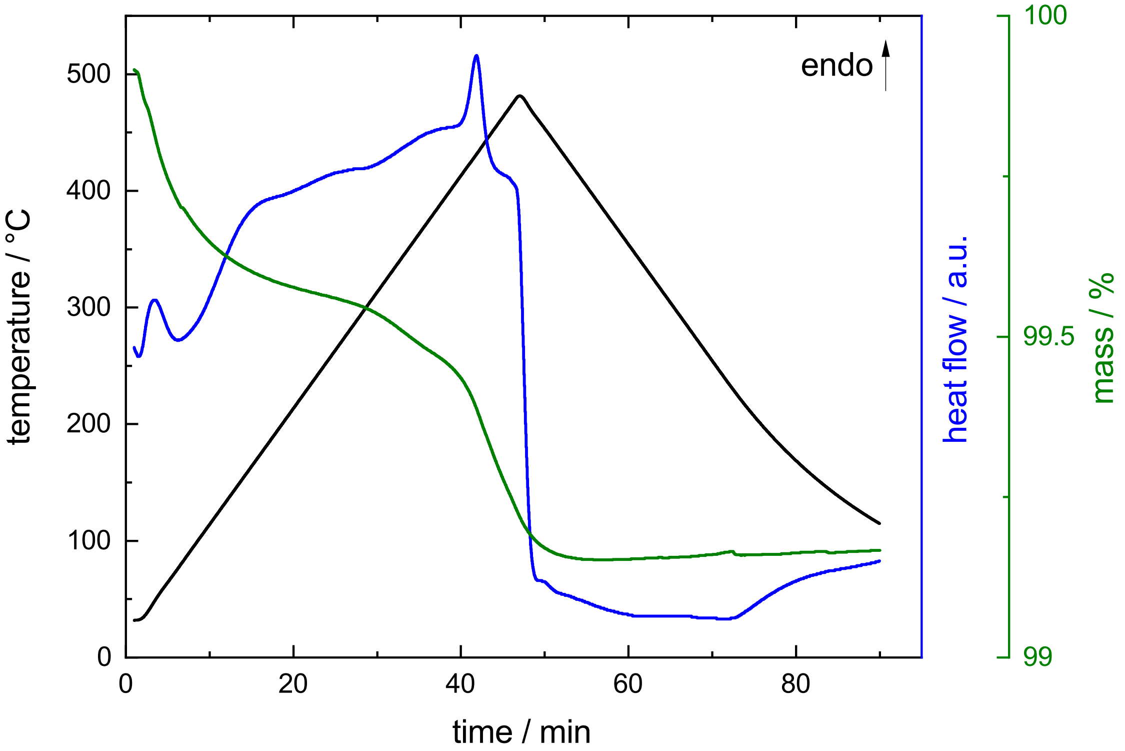

Supplementary Figure 32. TGA/DSC data for the preparation of a_gTIF-4.

Supplementary Methods 6 - Photography

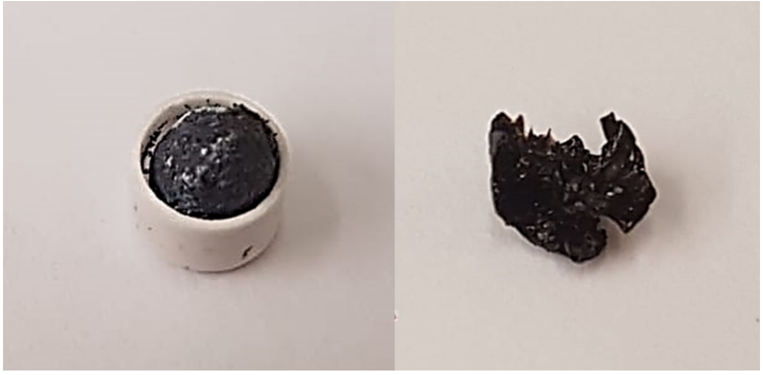

Supplementary Figure 33. Photographs of a_gZIF-4 obtained after the preparative TGS/DSC experiment.

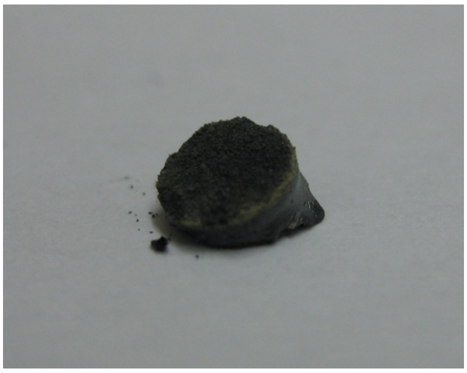

Supplementary Figure 34. Photograph of $a_gZIF-zni$ obtained after the preparative TGS/DSC experiment.

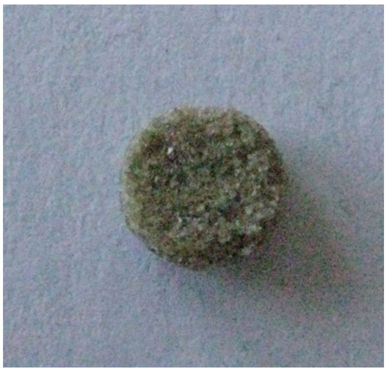

Supplementary Figure 35. Photograph of $a_gZIF-62$ obtained after the preparative TGS/DSC experiment.

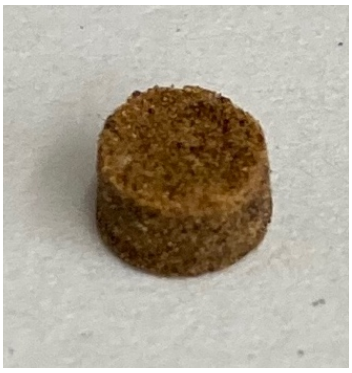

Supplementary Figure 36. Photograph of a_gTIF-4 obtained after the preparative TGA/DSC experiment.

Supplementary Methods 7 - Scanning electron microscopy

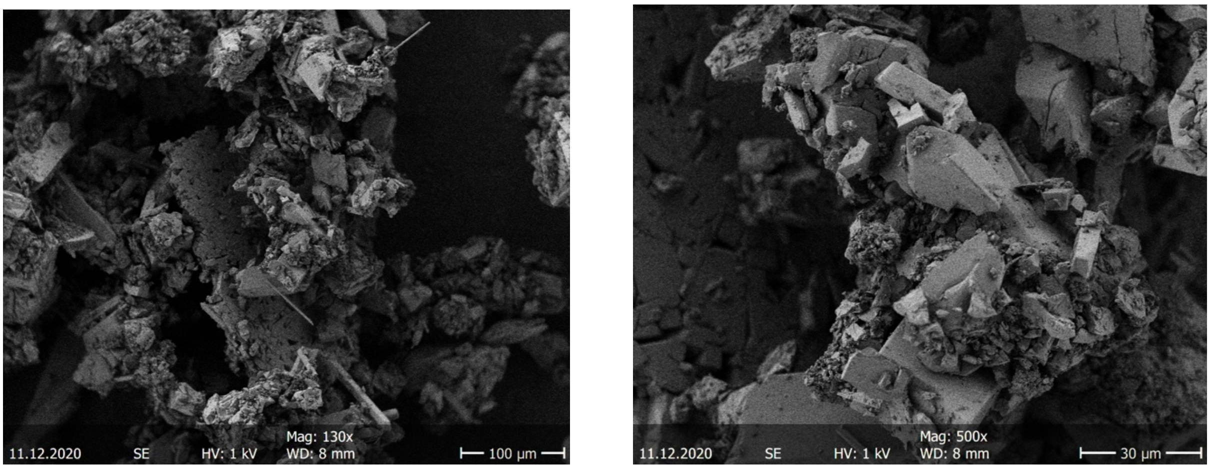

Supplementary Figure 37. SEM images of ZIF-zni (ground sample).

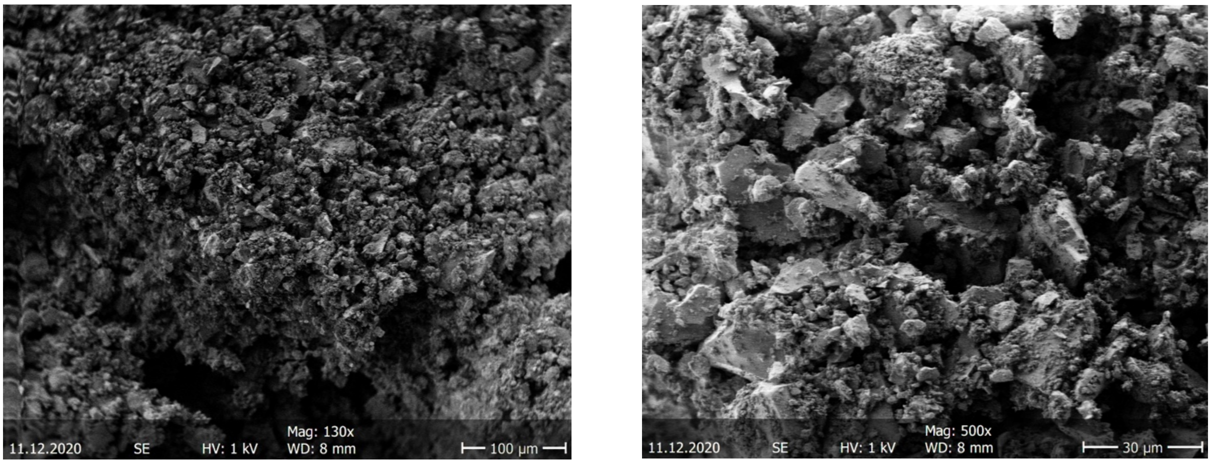

Supplementary Figure 38. SEM images of zni_TZIF-4 (ground sample).

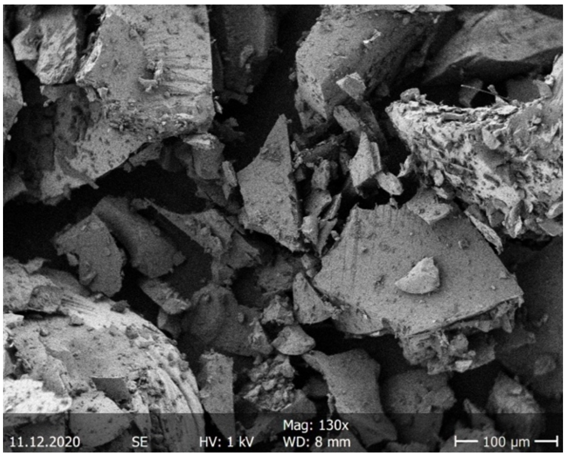

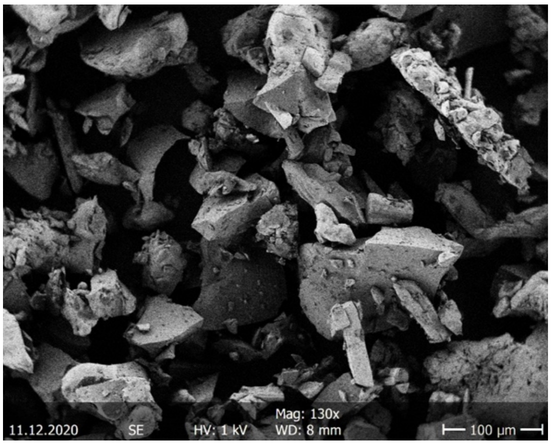

Supplementary Figure 39. SEM images of a_gZIF-4 (ground sample). **Supplementary Figure 40.** SEM images of a_gZIF-zni (ground sample).

Supplementary Methods 8 - X-ray total scattering data

X-ray total scattering data have been collected for all investigated materials. From these data, pair distribution functions in the form *D*(*r*) have been calculated showing long range order correlations (up to at least 50 Å) for all crystalline materials (ZIF-4, ZIF-zni, zni_TZIF-4, ZIF-62 and TIF-4) whereas the last intense peak is found at approx. 5.9 Å for all amorphous materials (a_TZIF-4, a_gZIF-4, a_gZIF-zni, a_gZIF-62, a_gTIF-4; see Figure 2e and Supplementary Figure 42). This peak equals the Zn-Zn distance in the materials. The data are in accordance with previously reported total scattering data.[4–8]

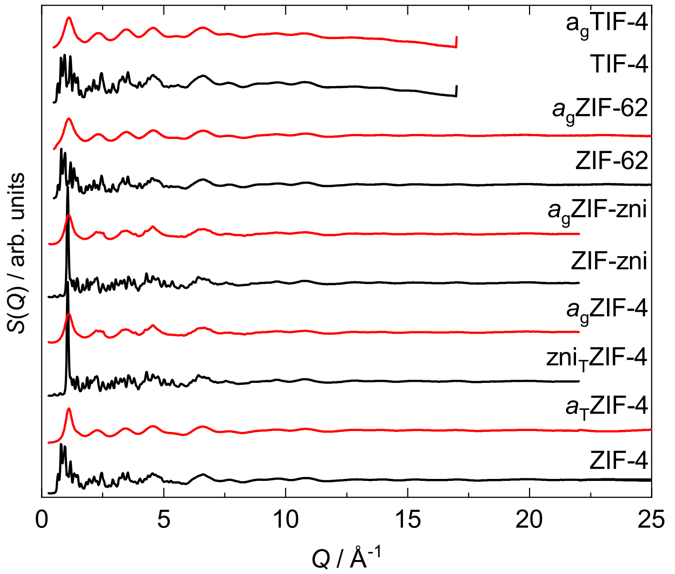

Supplementary Figure 41. X-ray total scattering data in the form *S*(*Q*) for all investigated materials. Crystalline and amorphous phases are displayed in black or red, respectively. Data for ZIF-62 and a_gZIF-62 are taken from ref. 1.

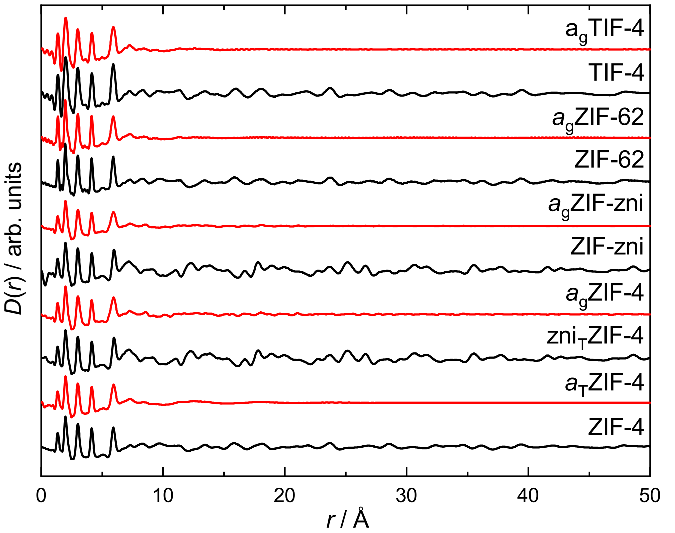

Supplementary Figure 42. Pair distribution functions in the form *D*(*r*) for all investigated materials. Crystalline and amorphous phases are displayed in black or red, respectively. Data for ZIF-62 and a_gZIF-62 are taken from ref. 1.

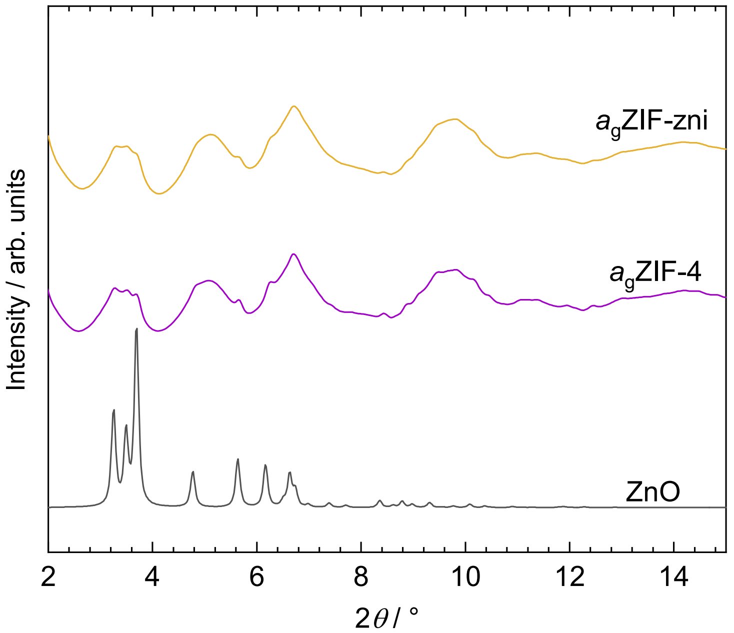

Supplementary Figure 43. Zoom into the background subtracted X-ray total scattering data of a_gZIF-4 and $a_gZIF-zni$ compared to a simulated XRPD pattern of ZnO (crystallographic data taken from ref. 9). It becomes evident that the small but sharp peaks found in a_gZIF-4 and $a_gZIF-zni$ can be ascribed to ZnO.

Supplementary Methods 8.1 - Fitting of the first sharp diffraction peak (FSDP)

The FSDP of the total scattering data in the form *S*(*Q*) (see Supplementary Figure 44) has been fitted to a pseudo-Voigt function for all investigated amorphous materials. From these fits we obtained the position of the FSDP (Q_{FSDP}) and the peak width at half maximum (ΔQ_{FSDP}) (see Supplementary Table 5). The calculations have been performed with the Fityk software package.[10]

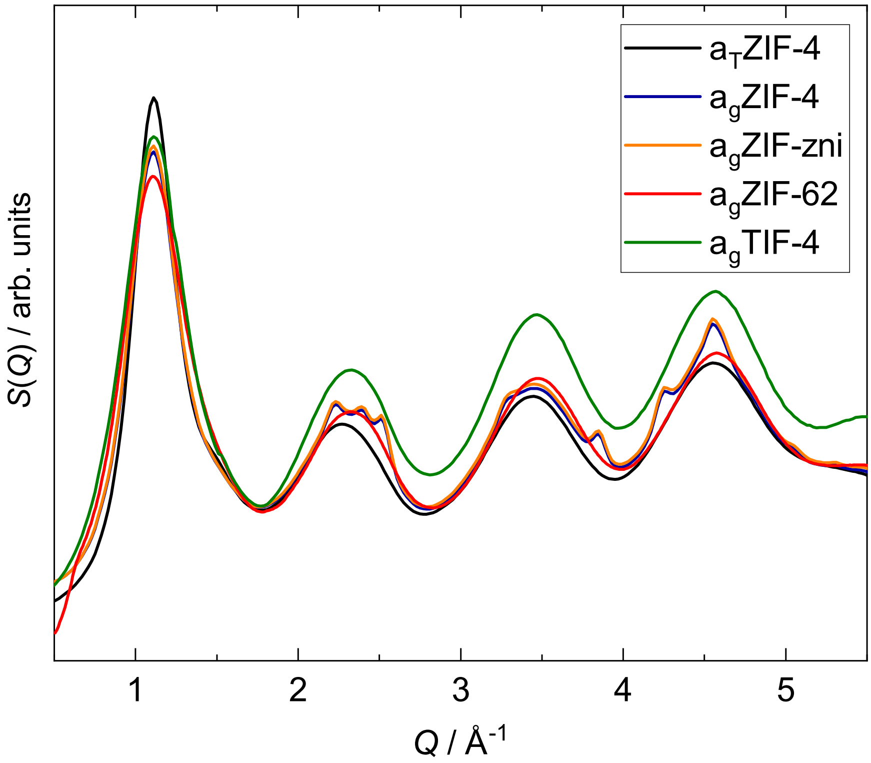

Supplementary Figure 44. Overlay of X-ray total scattering data in the form *S*(*Q*) for all investigated amorphous materials. Zoom in the region from 0.5-5.5 Å^{-1}. Data for a_gZIF-62 are taken from ref. 1**.**

Supplementary Table 5. Summary of the parameter obtained after fitting of the first sharp diffraction peak of the total scattering data for all investigated amorphous phases.

	Q_{FSDP} / Å^{-1}	ΔQ_{FSDP} / Å^{-1}
a_TZIF-4	1.108	0.244
a_gZIF-4	1.097	0.286
a_gZIF-zni	1.105	0.287
a_gZIF-62	1.098	0.350
a_gTIF-4	1.109	0.348

Supplementary Methods 9 - Isothermal gas physisorption data

N_2 physisorption studies

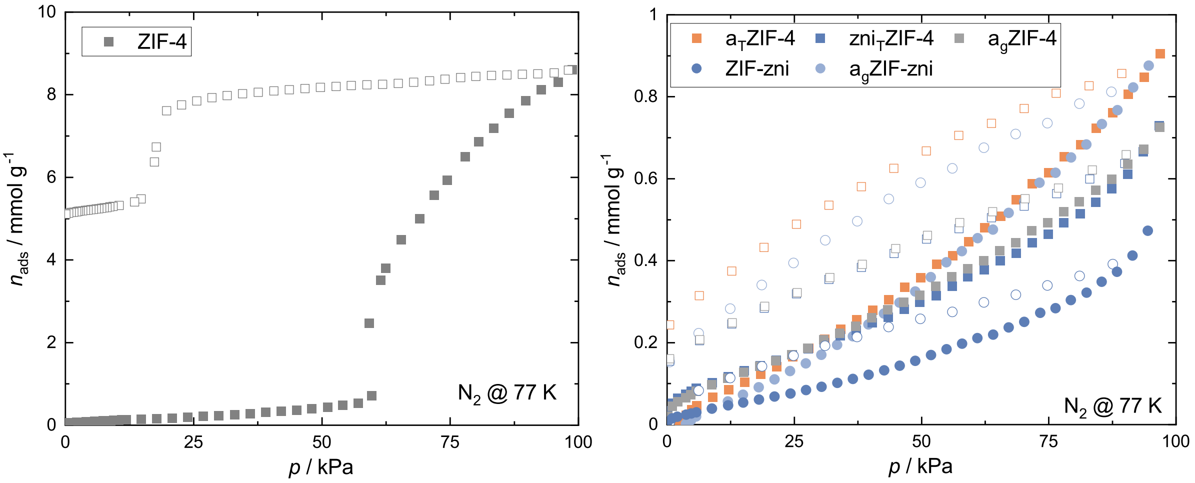

Supplementary Figure 45. N_2 sorption isotherms recorded at 77 K for ZIF-4 (left) and the corresponding thermal products as well as ZIF-zni and its corresponding glass (right). Adsorption and desorption branches are shown as close and open symbols, respectively. ZIF-4 shows a previously reported breathing behaviour upon adsorption of N_2.[11–13]

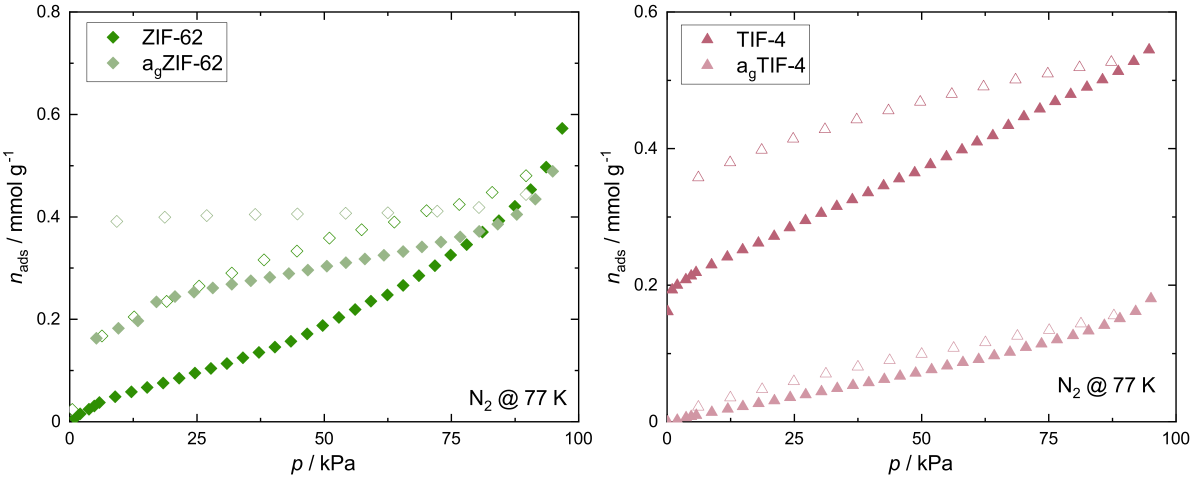

Supplementary Figure 46. N_2 sorption isotherms recorded at 77 K for ZIF-62 and a_gZIF-62 (left) and TIF-4 and a_gTIF-4 (right). Adsorption and desorption branches are shown as close and open symbols, respectively.

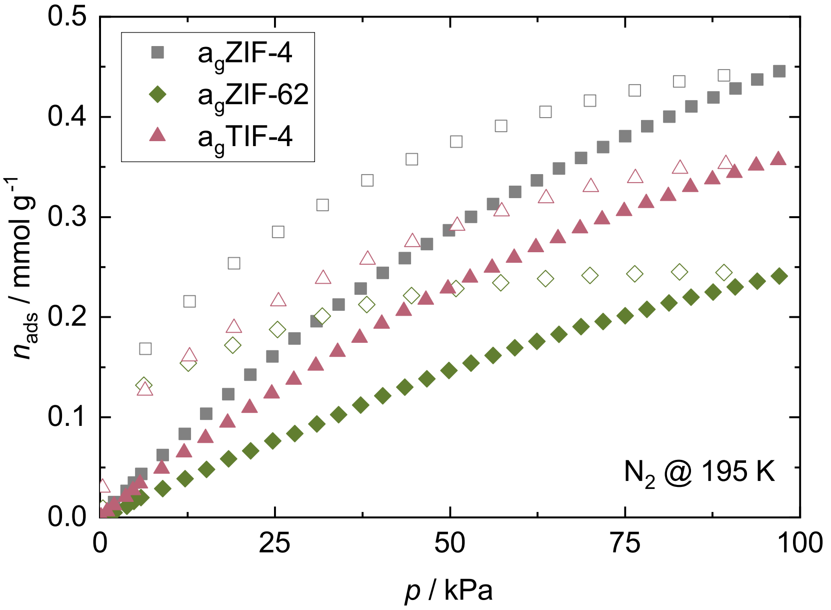

Supplementary Figure 47. N_2 sorption isotherms recorded at 195 K for a_gZIF-4, a_gZIF-62 and a_gTIF-4. Adsorption and desorption branches are shown as close and open symbols, respectively. Notice that N_2 is far above its critical temperature (T_c = 126.192 K) at 195 K.

Ar physisorption studies

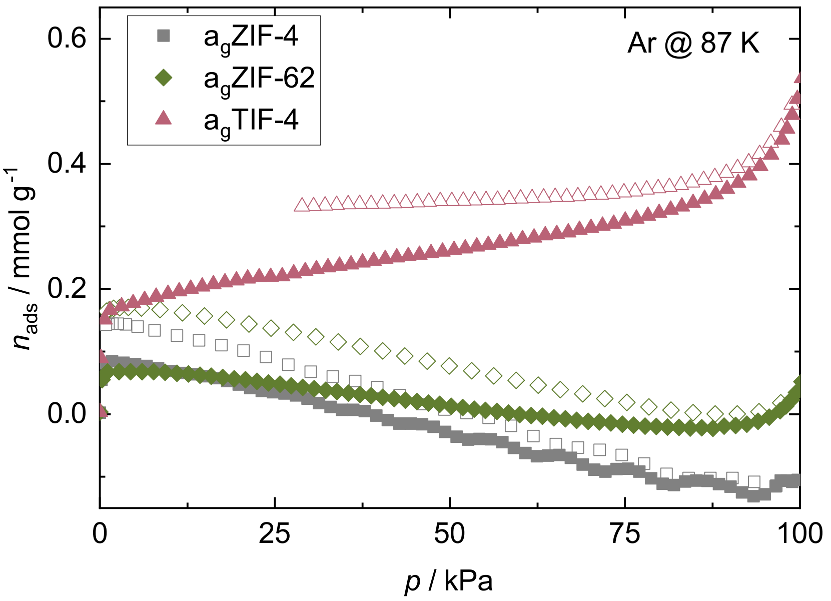

Supplementary Figure 48. Ar sorption isotherms recorded at 87 K for a_gZIF-4, a_gZIF-62 and a_gTIF-4. Adsorption and desorption branches are shown as close and open symbols, respectively. The negative uptake of a_gZIF-62 and a_gZIF-4 is a measurement error due to the inaccessibility of the materials pores for Ar gas.

CO_2 physisorption studies

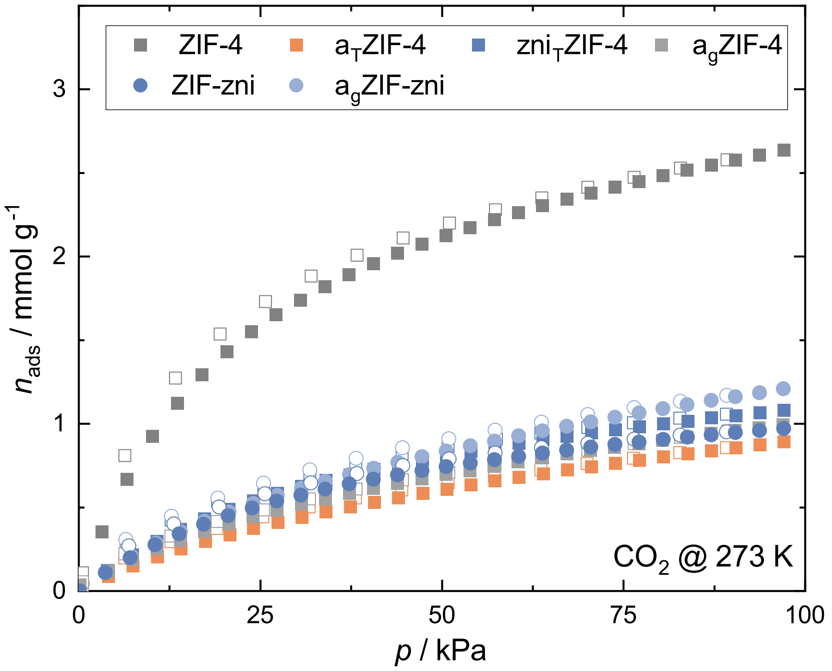

Supplementary Figure 49. CO_2 sorption isotherms recorded at 273 K for ZIF-4 and ZIF-zni and their corresponding thermal products. Adsorption and desorption branches are shown as close and open symbols, respectively.

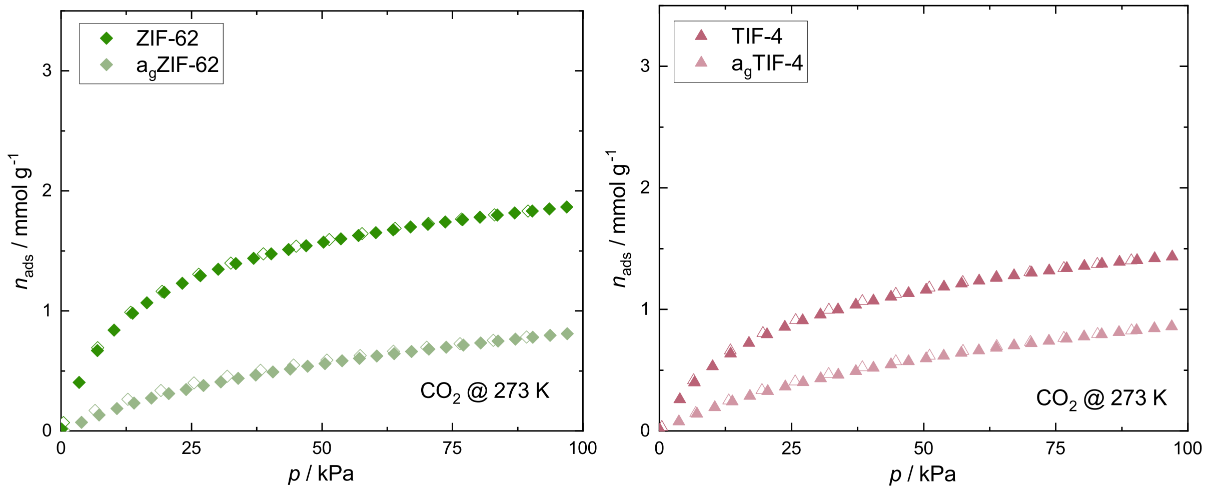

Supplementary Figure 50. CO_2 sorption isotherms recorded at 273 K for ZIF-62 (data are taken from ref. 1) and a$_g$ZIF-62 (left) as well as TIF-4 and a$_g$TIF-4 (right). Adsorption and desorption branches are shown as close and open symbols, respectively.

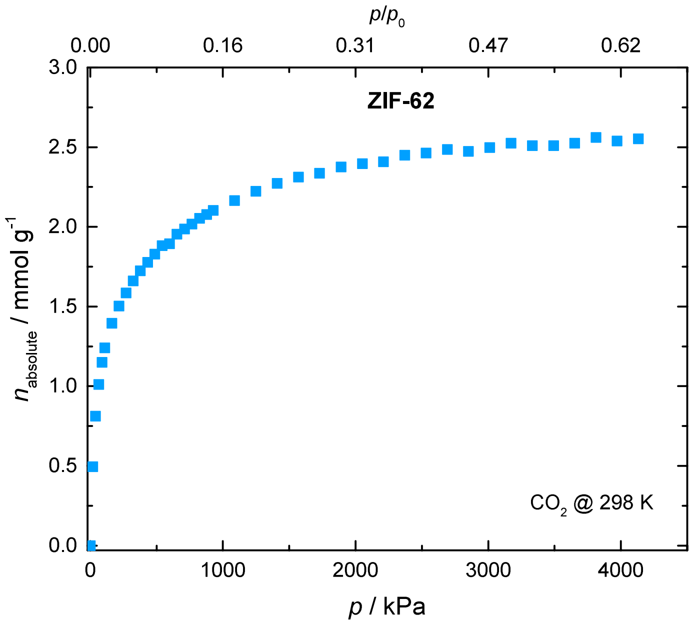

Supplementary Figure 51. High-pressure CO_2 adsorption isotherm of ZIF-62 recorded at 298 K with a maximum pressure of about 4130 kPa (corresponding to p/p_0 = 0.64). The absolute uptake ($n_{absolute}$) calculated from the measured excess uptake (n_{excess}) is shown: $\boldsymbol{n_{absolute}} = \boldsymbol{n_{excess}} + \boldsymbol{V_{pore}} \cdot \boldsymbol{\rho_{mol}}$; with V_{pore} = pore volume of the adsorbent and ρ_{mol} = molar gas phase density of the adsorptive. The pressure dependent molar density of CO_2 were obtained from National Institute of Standards and Technology (NIST, https://webbook.nist.gov/chemistry/fluid/) and is based on an equation of state for CO_2 developed by Span and Wagner.[14] The density data were fit to a fifth order polynomial (**Supplementary Figure** 52) in order to be utilized for the calculation of $n_{absolute}$ from n_{excess}.

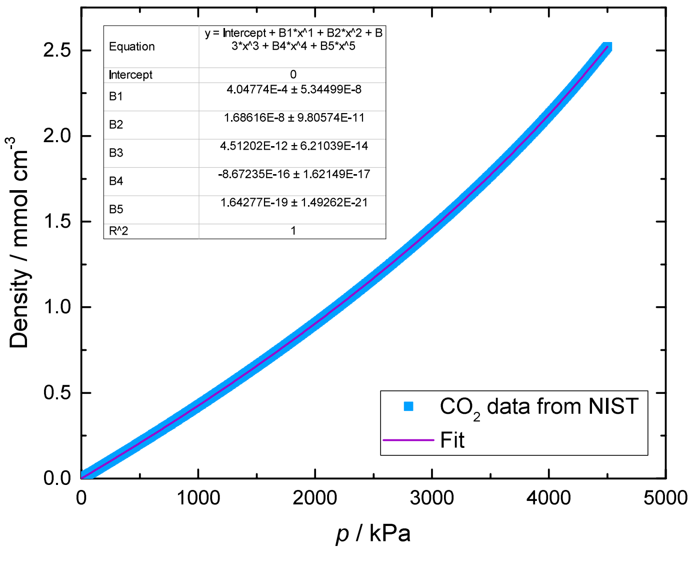

Supplementary Figure 52. Fifth order polynomial fit to the pressure-dependent molar gas phase density of CO_2 derived from NIST Chemistry Webbook (see caption of **Supplementary Figure** 51 for details).

The absolute CO_2 uptake recorded at 298 K and 4130 kPa amounts to 2.55 mmol g^{-1}. By considering the molar mass of CO_2 (44.009 g mol^{-1}) and the saturated liquid phase density of CO_2 at 298 K (0.7128 g cm^{-3}), we determine a pore volume of ZIF-62 of 0.16 cm^3 g^{-1}. This is the same value as the one determined from the CO_2 sorption isotherms recorded at 195 K (applying the density of the supercooled liquid of CO_2 extrapolated to 195 K, see Supplementary Figure 62). Hence, the high-pressure CO_2 sorption data verify the robustness of our data analysis of the CO_2 sorption data recorded at 195 K.

n-Butane physisorption studies

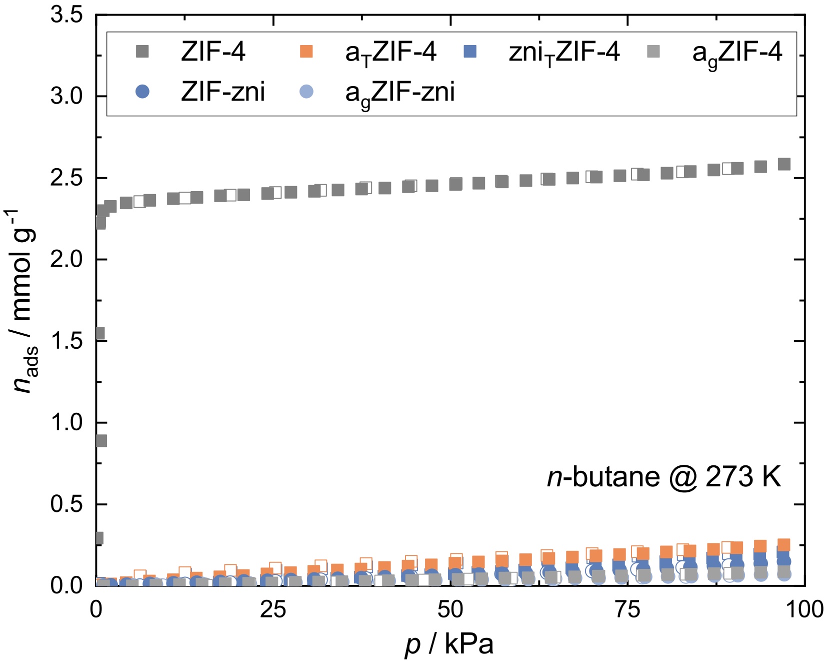

Supplementary Figure 53. *n*-Butane sorption isotherms recorded at 273 K for ZIF-4 and ZIF-zni and their corresponding thermal products. Adsorption and desorption branches are shown as close and open symbols, respectively.

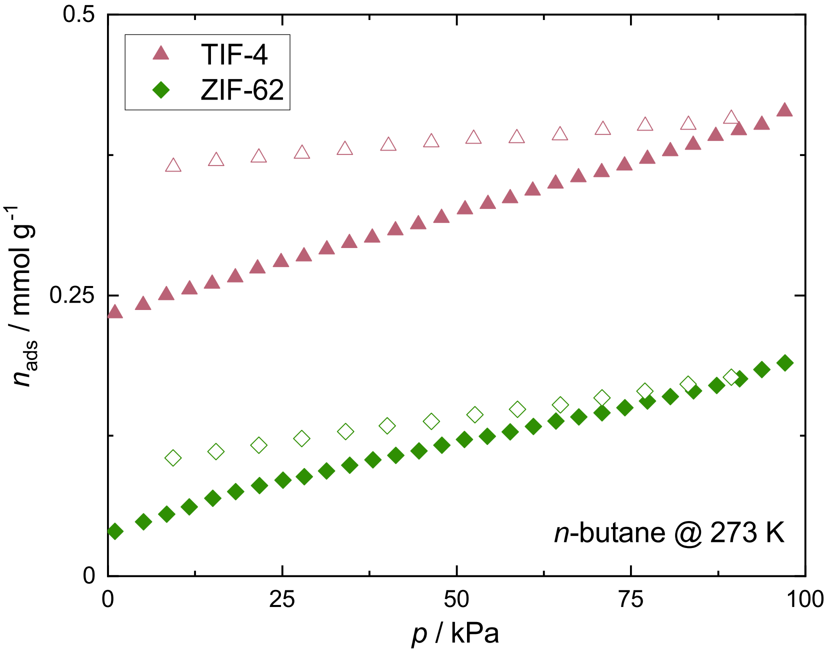

Supplementary Figure 54. *n*-Butane sorption isotherms recorded at 273 K for ZIF-62 and TIF-4. Adsorption and desorption branches are shown as close and open symbols, respectively.

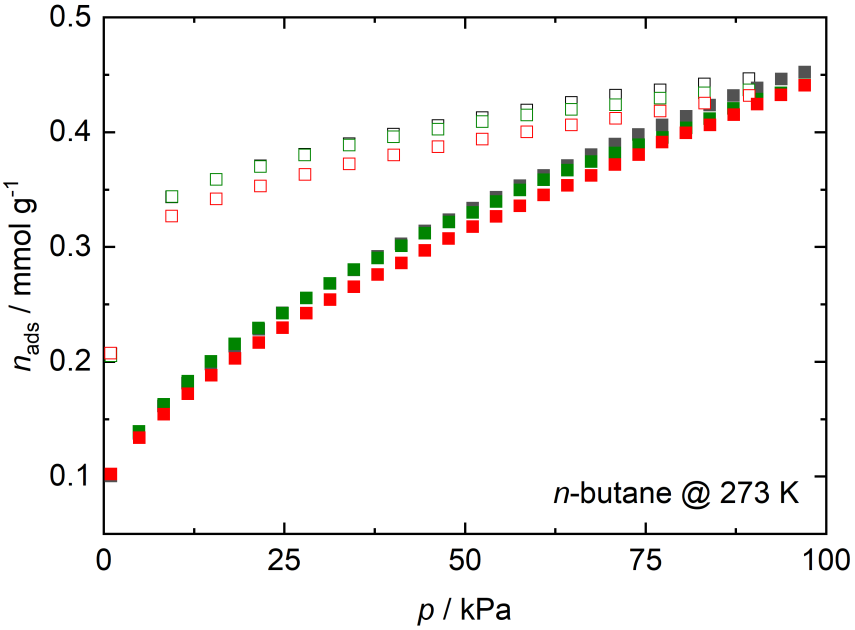

Supplementary Figure 55. *n*-Butane sorption isotherms recorded at 273 K for a_gZIF-62. The same material was repeatedly measured three times (colour code: 1st cycle black squares; 2nd cycle green squares; 3rd cycle red squares). Between the measurement, the materials were degassed for 30 min at 200 °C to desorb the residual *n*-butane from the previous measurement. Adsorption and desorption branches are shown as close and open symbols, respectively. The repeatability of the experiment signifies that the observed hystereses are not associated with structural changes of the glass during gas sorption (i.e. a swelling or breathing behaviour).

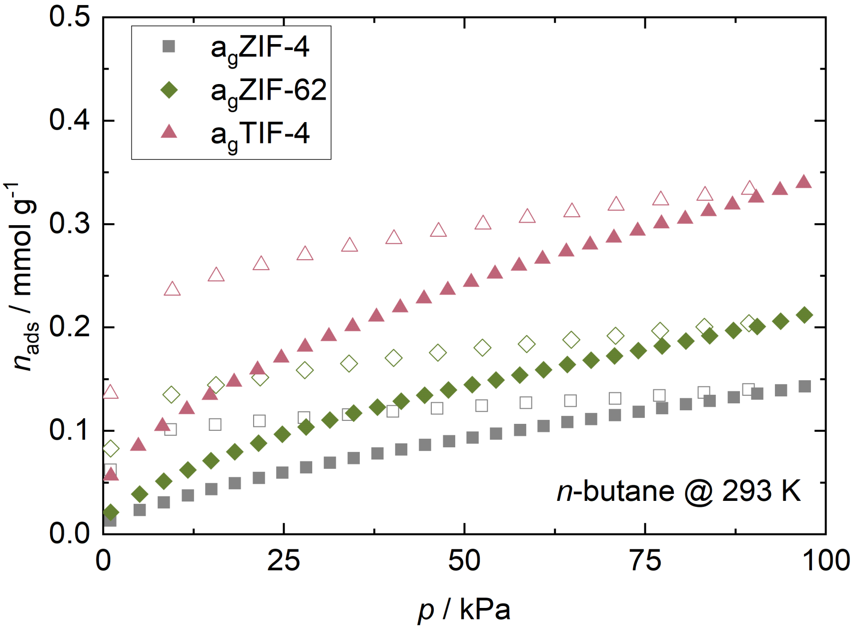

Supplementary Figure 56. *n*-Butane sorption isotherms recorded at 293 K for a_gZIF-4, a_gZIF-62 and a_gTIF-4. Adsorption and desorption branches are shown as close and open symbols, respectively.

Propane and propylene physisorption studies

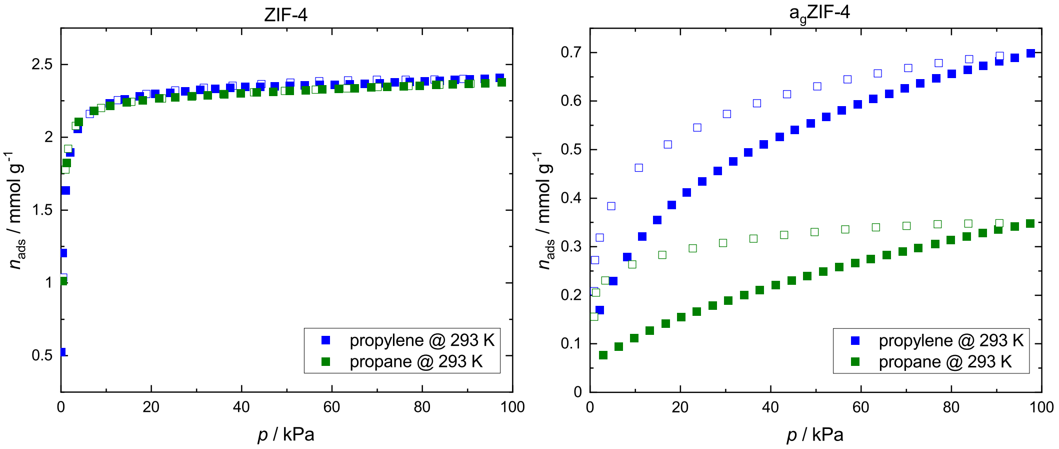

Supplementary Figure 57. Propane and propylene isotherms recorded at 293 K for ZIF-4 (left) and a_gZIF-4 (right). Adsorption and desorption branches are shown as close and open symbols, respectively. ZIF-4 shows similar total uptakes for both C3 hydrocarbons with a slightly higher affinity for propane at lower pressures which has been reported in the literature before.[15]

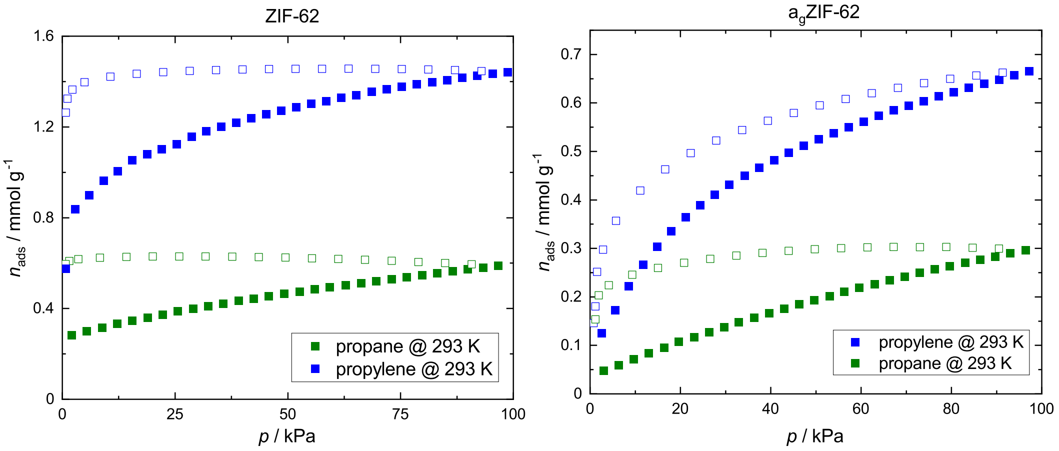

Supplementary Figure 58. Propane and propylene isotherms recorded at 293 K for ZIF-62 (left) and a_gZIF-62 (right). Adsorption and desorption branches are shown as close and open symbols, respectively.

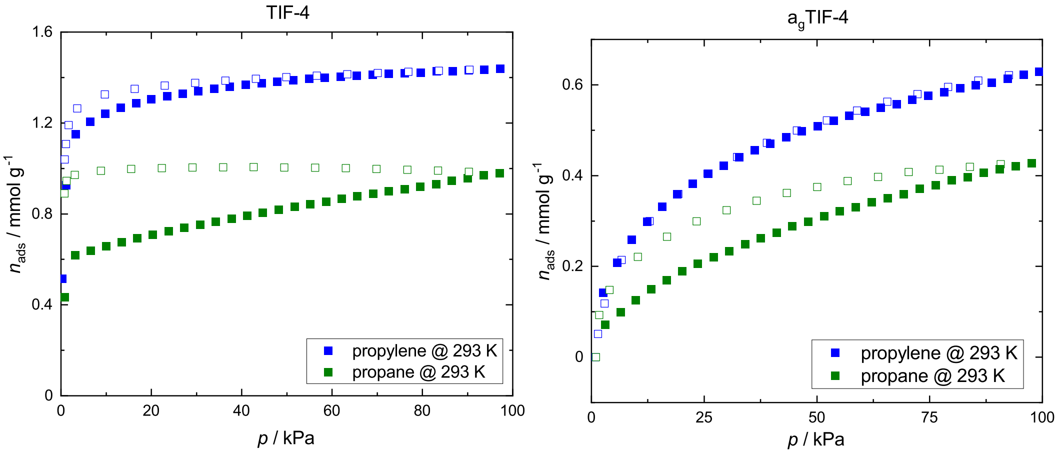

Supplementary Figure 59. Propane and propylene isotherms recorded at 293 K for TIF-4 (left) and a_gTIF-4 (right). Adsorption and desorption branches are shown as close and open symbols, respectively.

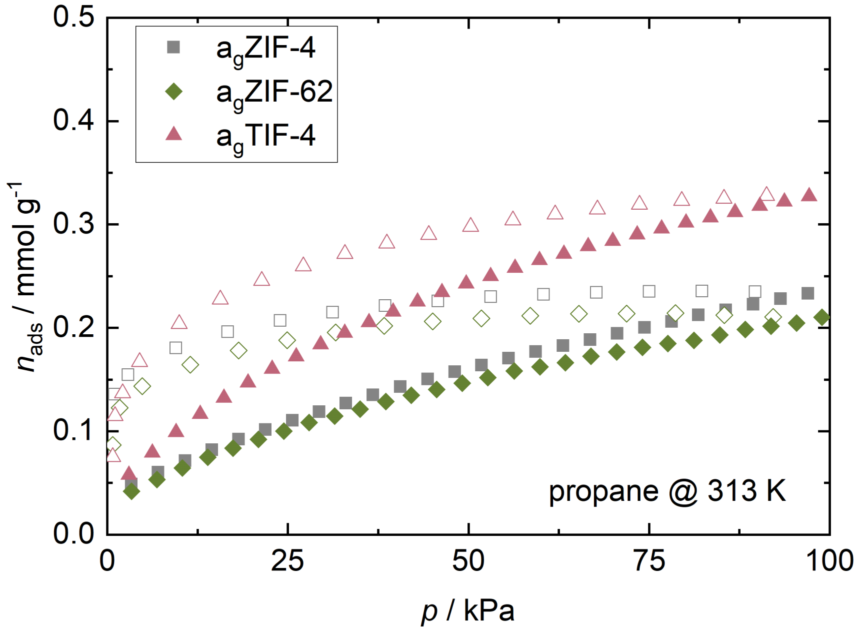

Supplementary Figure 60. Propane isotherms recorded at 313 K for a_gZIF-4, a_gZIF-62 and a_gTIF-4. Adsorption and desorption branches are shown as close and open symbols, respectively.

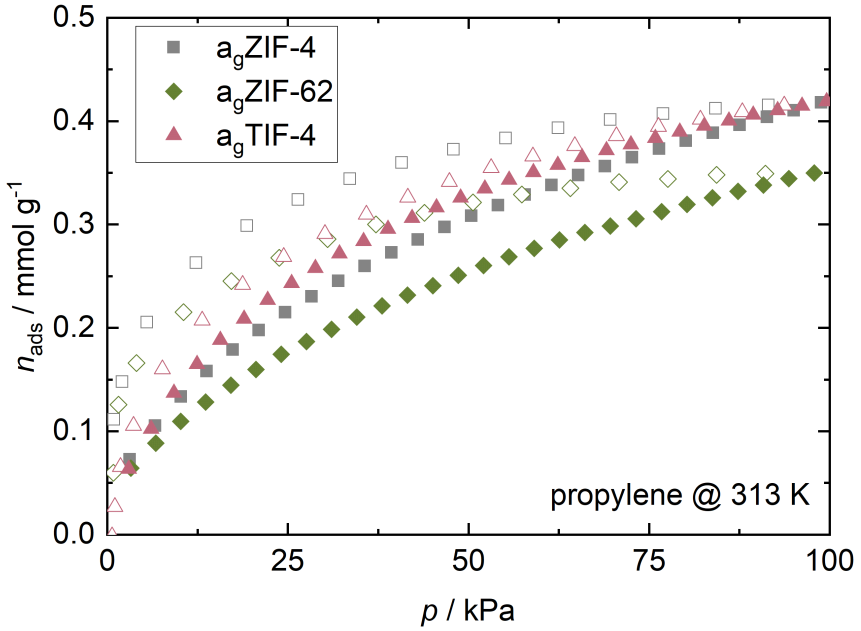

Supplementary Figure 61. Propylene isotherms recorded at 313 K for a_gZIF-4, a_gZIF-62 and a_gTIF-4. Adsorption and desorption branches are shown as close and open symbols, respectively.

Supplementary Methods 9.1 - Summary of adsorption capacities of MOF glasses

Supplementary Table 6. Summary of maximum adsorption capacities of CO_2 at approx. 95 kPa at different temperatures of this study and the so far published data for other ZIF glasses. Capacities are given in mmol g^{-1}.

compound	chemical composition	CO_2 @ 195 K	CO_2 @ 273 K	CO_2 @ 298 K	reference
ZIF-4	Zn(im)$_2$	7.22	2.64		this work
a_TZIF-4	Zn(im)$_2$	2.49	0.89		this work
zni$_T$-ZIF-4	Zn(im)$_2$	1.23	1.08		this work
a_gZIF-4	Zn(im)$_2$	2.84	1.00		this work
ZIF-zni	Zn(im)$_2$	1.21	0.97		this work
a_gZIF-zni	Zn(im)$_2$	3.12	1.21		this work
ZIF-62	Zn(im)$_{1.65}$(bim)$_{0.35}$	4.70	1.87[g]		this work
a_gZIF-62	Zn(im)$_{1.65}$(bim)$_{0.35}$	3.34	0.81		this work
TIF-4	Zn(im)$_{1.68}$(mbim)$_{0.32}$	3.76	1.43		this work
a_gTIF-4	Zn(im)$_{1.68}$(mbim)$_{0.32}$	3.40	0.86		this work
a_gZIF-62/ a_gZIF-62(Zn)-bim$_{0.35}$ [a, b]	Zn(im)$_{1.65}$(bim)$_{0.35}$	-	1.02		1
a_gZIF-62(Zn)-bim$_{0.17}$ [a]	Zn(im)$_{1.83}$(bim)$_{0.17}$	-	1.15		1
a_gZIF-62(Zn)-bim$_{0.05}$ [a]	Zn(im)$_{1.95}$(bim)$_{0.05}$	-	1.06		1
a_gZIF-UC-2	Zn(im)$_{1.90}$(6-Cl-5-Fbim)$_{0.10}$	-	≈0.48 [c]		16
a_gZIF-UC-3	Zn(im)$_{1.75}$(5-Cl-2-mbim)$_{0.25}$	-	≈0.86 [c]		16
a_gZIF-UC-4	Zn(im)$_{1.63}$(5-Fbim)$_{0.37}$	-	≈1.12 [c]		16
a_gZIF-UC-5	Zn(im)$_{1.69}$(5-Clbim)$_{0.31}$	-	≈0.95 [c]		16
a_g[(ZIF-8)$_{0.2}$(ZIF-62)$_{0.8}$]	-[d]	-	0.83		17
a_g(ZIF-62)	Zn(im)$_{1.76}$(bim)$_{0.24}$	-	0.90		18
a_gZIF-76	Zn(im)$_{1.0}$(5-Clbim)$_{1.0}$[e]	-	≈1.06[f]		19
a_gZIF-76-mbim	Zn(im)$_{0.93}$(mbim)$_{1.07}$[e]	-	≈1.63[f]		19
a_gZIF-UC-1a	Zn(im)$_{1.74}$(bim)$_{0.17}$(mbim)$_{0.09}$	-	-	≈0.78[f]	20
a_gZIF-UC1e	Zn(im)$_{1.61}$(bim)$_{0.18}$(mbim)$_{0.21}$	-	-	≈0.72[f]	20

Abbreviations: im$^-$ = imidazolate, bim$^-$ = benzimidazolate, 6-Cl-5-Fbim$^-$ = 6-chloro-5-fluorobenzimidazolate, 5-Cl-2-mbim$^-$ = 5-chloro-2-methylbenzimidazolate, 5-Fbim$^-$ = 5-fluorobenzimidazolate, 5-Clbim$^-$ = 5-chlorobenzimidazolate, mbim$^-$ = 2-methylbenzimidazolate.

---

[a] a_gZIF-62(M)-bim$_x$ refers to the glass derived from the former crystalline ZIF-62(M)-bim$_x$ material with the generalized chemical composition M(im)$_{2-x}$(bim)$_x$. The nomenclature is adopted from the corresponding publication.

[b] The abbreviation a_gZIF-62(Zn)-bim$_{0.35}$ equals a_gZIF-62 in this publication which was here shortened for clarity.

[c] The molar capacities are approximated from figures in the literature. Therefore, the capacities in cm^3(STP) cm^{-3} were read out from the figure in the publication, divided by the provided materials' pychnometric densities (g cm^{-3}, also read out from the corresponding figure) and then divided by the molar gas volume (STP).

[d] The chemical composition for ZIF-8 and ZIF-62 is here reported as Zn(mim)$_2$ and Zn(im)$_{1.75}$(bim)$_{0.25}$, respectively.

[e] Chemical composition for the crystalline precursor. Chemical composition of the glass is not reported.

[f] The molar capacities are approximated from figures in the literatures. The capacities in cm^3(STP) g^{-1} were read out from the figure in the publication and then divided by the molar gas volume (STP).

[g] Data are taken from ref. 1.

Supplementary Table 7. Summary of adsorption capacities of N_2 and investigated hydrocarbons at approx. 95 kPa at different temperatures. Capacities are given in mmol g^{-1}.

compound	N_2		*n*-butane		propane		propylene		Ar
	@ 77 K	@ 195 K	@ 273 K	@ 293 K	@ 293 K	@ 313 K	@ 293 K	@ 313 K	@ 87 K
ZIF-4	8.57	-	2.59	-	2.38	-	2.41	-	-
a_TZIF-4	0.91	-	0.25	-	-	-	-	-	-
zni_T-ZIF-4	0.73	-	0.21	-	-	-	-	-	-
a_gZIF-4	0.72	0.45	0.09	0.14	0.35	0.23	0.70	0.42	≈ 0[a]
ZIF-zni	0.47	-	0.15	-	-	-	-	-	-
a_gZIF-zni	0.88	-	0.07	-	-	-	-	-	-
ZIF-62	0.49	-	0.19	-	0.59	-	1.44	-	-
a_gZIF-62	0.57	0.24	0.44	0.21	0.30	0.21	0.66	0.35	≈ 0[a]
TIF-4	0.54	-	0.41	-	0.98	-	1.44	-	-
a_gTIF-4	0.18	0.36	0.52	0.34	0.43	0.33	0.63	0.42	0.40

[a] The isotherms display a negative gas uptake for these materials. The pores are inaccessible for Ar at 87 K.

Supplementary Methods 9.2 - Surface area and pore volume analysis

BET[21] surface areas were determined with the Quantachrome ASIQwin version 5.2 software. The applied relative pressure ranges and quality factors are given in Supplementary Table 8. We again note that the BET model is not applicable to microporous materials. Hence, the BET areas must be taken with great care and cannot be considered as absolute physical values. We provide the values of the BET areas for comparison purposes only.

The specific micropore volumes (V_{pore}) were calculated according to:

$$V_{\text{pore}} = \frac{n_{\text{ads}}^{\text{max}} \cdot M_{\text{CO2}}}{\rho_{\text{sl}}} \text{ (see ref. 22)} \qquad (1)$$

with $n_{\text{ads}}^{\text{max}}$ the specific molar amount of gas adsorbed (mmol of gas/g material) at 195 K and 95 kPa, M_{CO2} the molar mass of CO_2, and ρ_{sl} the density of the supercooled liquid at 195 K (that is 1.258 g cm^{-3}). In analogy to previous reports[23], ρ_{liq} is obtained from the linear extrapolation of the tabulated liquid phase density of CO_2 from its triple point temperature (216.592 K) to 195 K (Supplementary Figure 62). Reference data for the liquid phase density of CO_2 as a function of temperature are taken from the NIST Chemistry Webbook (https://webbook.nist.gov/cgi/cbook.cgi?ID=C124389). The obtained values are summarized in Supplementary Table 8 and S9.

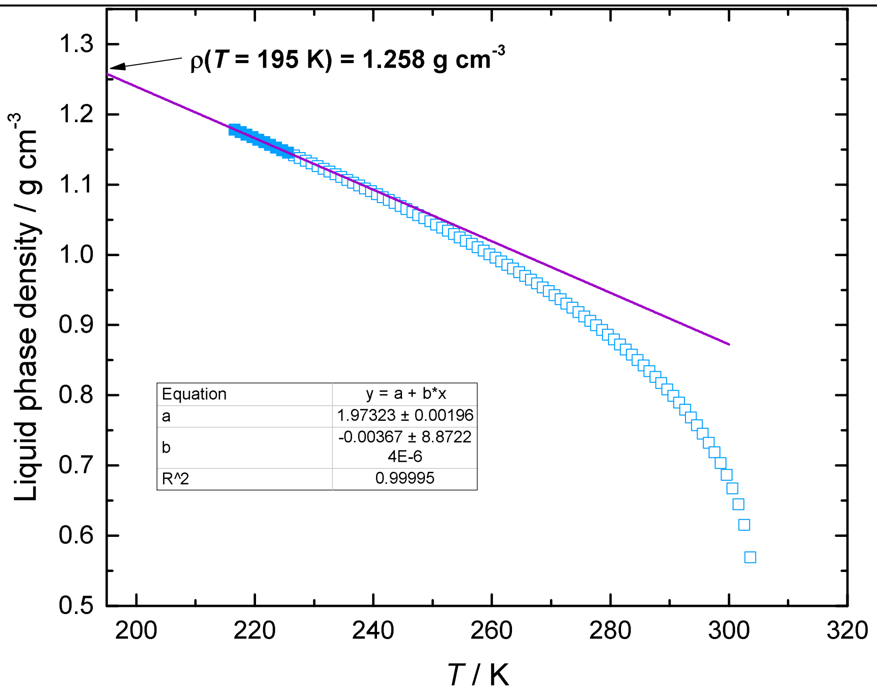

Supplementary Figure 62. Temperature dependent liquid phase density of CO_2 taken from NIST Chemistry Webbook (cyan squares) and linear fit to the data from 216.592 K to 225.592 K (purple line). Data points included in the fit are drawn as filled squares, data not included as open squares.

The experimental void fractions (eVF) were calculated according to:

$$\mathrm{eVF} = V_{\mathrm{pore}} \cdot \rho \qquad (2)$$

with the density ρ of the solid, either obtained from the crystallographic data (crystalline ZIFs) or obtained from the apparent density approximation (glassy ZIFs, see Section 9.4).

Supplementary Table 8. Pore analysis data for the CO_2 gas physisorption studies.

compound	gas	temperature	BET analysis				Specific micropore volume (V_{pore})[a]
			p/p_0 range	C constant	correlation coefficient	BET surface area[b]	
ZIF-4	CO_2	273 K[c]	0.008-0.027	126.2	0.999	410 $m^2\ g^{-1}$	-
	CO_2	195 K[d]	0.0006-0.029	169.6	0.995	490 $m^2\ g^{-1}$	0.25 $cm^3\ g^{-1}$
a_TZIF-4	CO_2	273 K[c]	0.011-0.027	41.96	0.999	199 $m^2\ g^{-1}$	-
	CO_2	195 K[d]	0.0006-0.036	258.5	0.994	145 $m^2\ g^{-1}$	0.09 $cm^3\ g^{-1}$
zni_TZIF-4	CO_2	273 K[c]	0.009-0.027	75.60	0.999	193 $m^2\ g^{-1}$	-
	CO_2	195 K[d]	0.0006-0.039	1657	0.999	92 $m^2\ g^{-1}$	0.04 $cm^3\ g^{-1}$
a_gZIF-4	CO_2	273 K[c]	0.009-0.027	51.68	0.999	203 $m^2\ g^{-1}$	-
	CO_2	195 K[d]	0.0006-0.026	323.7	0.999	187 $m^2\ g^{-1}$	0.10 $cm^3\ g^{-1}$
ZIF-zni	CO_2	273 K[c]	0.006-0.027	84.40	0.999	168 $m^2\ g^{-1}$	-
	CO_2	195 K[d]	0.0005-0.025	1154	0.999	94 $m^2\ g^{-1}$	0.04 $cm^3\ g^{-1}$
a_gZIF-zni	CO_2	273 K[c]	0.005-0.027	52.63	0.998	243 $m^2\ g^{-1}$	-
	CO_2	195 K[d]	0.0006-0.030	350.9	0.998	189 $m^2\ g^{-1}$	0.11 $cm^3\ g^{-1}$
ZIF-62	CO_2	273 K[c, e]	0.006-0.027	193.2	0.999	268 $m^2\ g^{-1}$	-
	CO_2	195 K[d]	0.0006-0.027	490.3	0.998	264 $m^2\ g^{-1}$	0.16 $cm^3\ g^{-1}$
a_gZIF-62	CO_2	273 K[c]	0.008-0.027	47.35	0.999	171 $m^2\ g^{-1}$	-
	CO_2	195 K[d]	0.0006-0.027	321.8	0.997	200 $m^2\ g^{-1}$	0.12 $cm^3\ g^{-1}$
TIF-4	CO_2	273 K[c]	0.009-0.027	125.7	0.999	224 $m^2\ g^{-1}$	-
	CO_2	195 K[d]	0.0005-0.027	392.7	0.997	185 $m^2\ g^{-1}$	0.13 $cm^3\ g^{-1}$
a_gTIF-4	CO_2	273 K[c]	0.002-0.027	53.70	0.998	171 $m^2\ g^{-1}$	-
	CO_2	195 K[d]	0.0006-0.026	333.5	0.997	204 $m^2\ g^{-1}$	0.12 $cm^3\ g^{-1}$

[a] $p \approx 95$ kPa, applied value for the density of adsorbate in its supercooled liquid state $\rho_{liq}(CO_2\ @195K) = 1.258\ g\ cm^{-3}$ (see Section S9.2 for further information)

[b] Applied value for cross sectional areas: $\sigma(CO_2\ @273\ K) = 21$ Å^2/molecule and $\sigma(CO_2\ @195\ K) = 17$ Å^2/molecule[24].

[c] $p_0(CO_2, 273K) = 3485$ kPa[25]

[d] $p_0(CO_2, 195K) = 191$ kPa[26]

[e] Raw data have been already included in ref. 1.

Supplementary Table 9. Comparison of the specific micropore volumes (V_{pore}) of a_gZIF-62 and a_gTIF-4 obtained from gas sorption isotherms of *n*-butane (@273 K) and CO_2 (@195 K).

	V_{pore} (*n*-butane)[a]	V_{pore} (CO_2)[b]	ratio
a_gZIF-62	0.043 $cm^3 g^{-1}$	0.12 $cm^3 g^{-1}$	35%
a_gTIF-4	0.050 $cm^3 g^{-1}$	0.12 $cm^3 g^{-1}$	42%

[a] $p \approx 95$ kPa, applied value for the density of pure liquid adsorbate ρ_{liq}(*n*-butane @273K) = 0.601 g cm^{-3}.[27]

[b] $p \approx 95$ kPa, applied value for the density of liquid adsorbate in its supercooled liquid state ρ_{liq}(CO_2 @195K) = 1.258 g cm^{-3}.

Supplementary Methods 9.3 - Pore size distribution analysis

In analogy to previous studies[1,19], experimental pore size distributions (PSDs) were derived from CO_2 isotherms at 273 K with the nonlocal density functional theory (NLDFT[28], carbon equilibrium transition kernel at 273 K based on a slit pore model[29]) using the Quantachrome ASIQwin version 5.2 software. This is the only NLDFT kernel implemented in the software packages of commercial gas physisorption analysers and thus often used in the MOF literature to derive PSDs.
Theoretical PSDs were calculated with the Zeo++[30] software package using the default CCDC radii (-ha 'high accuracy' flag[31]) and the implemented routine for pore size distributions. The probe size was 0.1 Å and 5000 Monte Carlo samples per unit cell were averaged. The structures for the calculations were taken from the CSD database (ZIF-4: CCDC code IMIDZB11; ZIF-zni: CCDC code IMIDZB). In both structures, missing hydrogen atoms have been added geometrically with Olex2 [32].

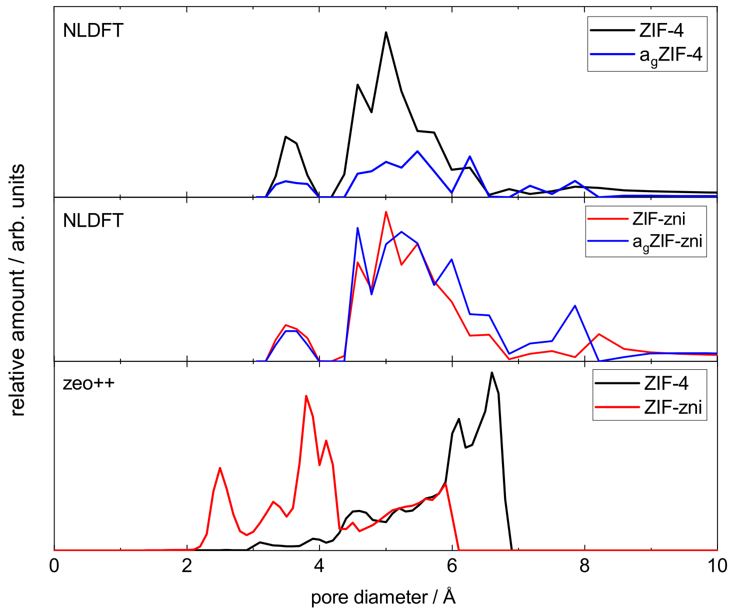

Supplementary Figure 63. Theoretical pore size distribution calculated with Zeo++ for crystalline ZIF-4 and ZIF-zni in comparison to experimental pore size distributions calculated from the CO_2 isotherms recorded at 273 K using the NLDFT method for the crystalline and glassy phases of these materials.

Supplementary Figure 63 demonstrates that the PSDs derived from experimental CO_2 sorption isotherms of crystalline ZIF-4 and ZIF-zni recorded at 273 K by the NLDFT model (carbon, slit pore) are inconsistent with the theoretical PSDs derived from their crystal structures. It is evident that the NLDFT model for carbon materials with slit pore geometry is inappropriate for the calculation of PSDs of ZIF materials. This must be ascribed to the very different surface electrostatics (non-polar carbon vs. appreciably polar ZIFs) and the different pore geometries of the ZIFs. Since the PSDs of the crystalline ZIFs is not described accurately by the utilized NLDFT model, we conclude that also the PSDs previously derived for ZIF glasses via the same method[1,19] do not represent a meaningful description of their pore structure.

Supplementary Methods 9.4 - Density approximation

The apparent densities of the glasses were determined from the correlation of the density to the specific pore volumes determined by CO_2 adsorption isotherms at 195 K.

Feasibility test

First, the feasibility of the correlation was proven with theoretical and experimental considerations for ZIF-4 and ZIF-zni. Therefore, the theoretical and experimental void fractions were calculated.

The theoretical void fractions (tVFs) – based on the crystal structures for both materials – were calculated with the implemented routine in Olex2[32] applying a probe radius of 1.6 Å and grid spacing of 0.2 Å. The structures were taken from the CSD database. In both structures, missing hydrogen atoms have been added geometrically with Olex2. The theoretical accessible pore space for ZIF-4 (CCDC code IMIDZB11) amounts to 28.7% and for ZIF-zni (CCDC code IMIDZB) to 7.5%.[a]

The experimental void fractions (eVFs) were calculated from the specific micropore volumes (V_{pore}) derived from the CO_2 adsorption isotherms at 195 K given in $cm^3\ g^{-1}$ (see Supplementary Table 8 and Supporting Information Section S9.2) multiplied by the crystallographic densities[b] (ρ_{cryst}) of the materials (eVF = $V_{pore} \cdot \rho_{cryst}$, see Supporting Information Section S9.2 for further details). The densities are 1.22 g cm^{-3} (ZIF-4) and 1.56 g cm^{-3} (ZIF-zni). The corresponding eVFs amount to 30.6% and 6.2% for ZIF-4 and ZIF-zni, respectively, which are in very good agreement with the tVFs, demonstrating the feasibility of the methodology.

Exponential fit

The V_{pore} vs. ρ_{cryst} data for the crystalline compounds ZIF-4, ZIF-zni were completed with the corresponding values for ZIF-62 and TIF-4 and fitted with an exponential fitting function (see Figure 3; R^2-value = 0.998).

$$V_{\text{pore}}(\rho) = a \cdot e^{-\frac{\rho}{b}} \qquad (3)$$

$V_{\text{pore}} = \text{specific pore volume}$ and $\rho = \text{density}$

with $a = 377.6 \pm 120.1$ $cm^3\ g^{-1}$ and $b = 0.167 \pm 0.007$ g cm^{-3}

Based on the fitting function the density of the ZIF glasses can be calculated from their experimental pore volumes.

---

[a] The same calculation has also been performed for ZIF-62 (CCDC code SIWJAM) and TIF-4 (CCDC code QOSYAZ). Before the calculation, some disordered groups were resolved and solvent molecules were removed where present. tVF values are given in Figure 1. We note that the comparison of these values to the corresponding eVFs is not applicable, because of some unresolvable residual disorder leading to partially occupied secondary linkers (bim^- or $mbim^-$).

[b] All densities for crystalline materials have been calculated from the mass of atoms in one unit cell and the unit cell volume determined via profile fits of XRPD data (see Supplementary Table 1).

Supplementary References

1. Frentzel-Beyme, L., Kloss, M., Kolodzeiski, P., Pallach, R. & Henke, S. Meltable Mixed-Linker Zeolitic Imidazolate Frameworks and Their Microporous Glasses - From Melting Point Engineering to Selective Hydrocarbon Sorption. *J. Am. Chem. Soc.* **141**, 12362–12371 (2019).
2. Bumstead, A. M. *et al.* Investigating the melting behaviour of polymorphic zeolitic imidazolate frameworks. *CrystEngComm* **22**, 3627–3637 (2020).
3. Ryder, M. R. *et al.* Identifying the role of terahertz vibrations in metal-organic frameworks: From gate-opening phenomenon to shear-driven structural destabilization. *Phys. Rev. Lett.* **113**, 215502 (2014).
4. Bennett, T. D. *et al.* Hybrid glasses from strong and fragile metal-organic framework liquids. *Nat. Commun.* **6**, 8079 (2015).
5. Zhang, J. *et al.* Structural evolution in a melt-quenched zeolitic imidazolate framework glass during heat-treatment. *Chem. Commun.* **55**, 2521–2524 (2019).
6. Bennett, T. D. & Cheetham, A. K. Amorphous metal-organic frameworks. *Acc. Chem. Res.* **47**, 1555–1562 (2014).
7. Bennett, T. D. *et al.* Melt-Quenched Glasses of Metal-Organic Frameworks. *J. Am. Chem. Soc.* **138**, 3484–3492 (2016).
8. Bennett, T. D. *et al.* Structure and properties of an amorphous metal-organic framework. *Phys. Rev. Lett.* **104**, 2–5 (2010).
9. Kihara, K. & Donnay, G. Anharmonic Thermal Vibrations in ZnO*. *Can. Mineral.* **23**, 647–654 (1985).
10. Wojdyr, M. Fityk : a general-purpose peak fitting program. *J. Appl. Crystallogr.* **43**, 1126–1128 (2010).
11. Bennett, T. D. *et al.* Thermal amorphization of zeolitic imidazolate frameworks. *Angew. Chem. Int. Ed.* **50**, 3067–3071 (2011).
12. Gandara-Loe, J. *et al.* New insights into the breathing phenomenon in ZIF-4. *J. Mater. Chem. A* **7**, 14552–14558 (2019).
13. Wharmby, M. T. *et al.* Extreme flexibility in a zeolitic imidazolate framework: Porous to dense phase transition in desolvated ZIF-4. *Angew. Chem. Int. Ed.* **54**, 6447–6451 (2015).
14. Span, R. & Wagner, W. A New Equation of State for Carbon Dioxide Covering the Fluid Region from the Triple-Point Temperature to 1100 K at Pressures up to 800 MPa. *J. Phys. Chem. Ref. Data* **25**, 1509–1596 (1996).
15. Hartmann, M., Böhme, U., Hovestadt, M. & Paula, C. Adsorptive Separation of Olefin/Paraffin Mixtures with ZIF-4. *Langmuir* **31**, 12382–12389 (2015).
16. Hou, J. *et al.* Halogenated Metal–Organic Framework Glasses and Liquids. *J. Am. Chem. Soc.* **142**, 3880–3890 (2020).
17. Longley, L. *et al.* Flux melting of metal–organic frameworks. *Chem. Sci.* **10**, 3592–3601 (2019).
18. Widmer, R. N. *et al.* Pressure promoted low-temperature melting of metal–organic frameworks. *Nat. Mater.* **18**, 370–376 (2019).
19. Zhou, C. *et al.* Metal-organic framework glasses with permanent accessible porosity. *Nat. Commun.* **9**, 5042 (2018).
20. Ríos Gómez, M. L., Lampronti, G. I., Yang, Y., Mauro, J. C. & Bennett, T. D. Relating structural disorder and melting in complex mixed ligand zeolitic imidazolate framework glasses. *Dalton Trans.* **49**, 850–857 (2020).

21. Brunauer, S., Emmett, P. H. & Teller, E. Adsorption of Gases in Multimolecular Layers. *J. Am. Chem. Soc.* **60**, 309–319 (1938).
22. Ongari, D. *et al.* Accurate characterization of the pore volume in microporous crystalline materials. *Langmuir* **33**, 14529–14538 (2017).
23. Nikolaev, K. M. & Dubinin, M. M. Adsorption properties of carbonaceous adsorbents. III. Isotherms for the adsorption of gases and vapors on active charcoals over a broad temperature range, including the critical region. *Izv. Akad. Nauk. SSSR, Seriya Khimicheskaya*, 1165 (1958).
24. Yang, W. *et al.* Selective CO2 uptake and inverse CO2/C2H2 selectivity in a dynamic bifunctional metal-organic framework. *Chem. Sci.* **3**, 2993–2999 (2012).
25. Stephan, P., Schaber, K., Stephan, K. & Mayinger, F. *Thermodynamik*. (Springer Berlin Heidelberg, 2013). doi:10.1007/978-3-642-30098-1
26. Branton, P. J., Hall, P. G., Treguer, M. & Sing, K. S. W. Adsorption of carbon dioxide, sulfur dioxide and water vapour by MCM-41, a model mesoporous adsorbent. *J. Chem. Soc. Faraday Trans.* **91**, 2041 (1995).
27. GESTIS-Stoffdatenbank - *n*-butane. Available at: https://gestis.dguv.de/data?name=010030.
28. Lastoskie, C., Gubbins, K. E. & Quirke, N. Pore size distribution analysis of microporous carbons: a density functional theory approach. *J. Phys. Chem.* **97**, 4786–4796 (1993).
29. Landers, J., Gor, G. Y. & Neimark, A. V. Density functional theory methods for characterization of porous materials. *Colloids Surfaces A Physicochem. Eng. Asp.* **437**, 3–32 (2013).
30. Willems, T. F., Rycroft, C. H., Kazi, M., Meza, J. C. & Haranczyk, M. Algorithms and tools for high-throughput geometry-based analysis of crystalline porous materials. *Microporous Mesoporous Mater.* **149**, 134–141 (2012).
31. Pinheiro, M., Martin, R. L., Rycroft, C. H. & Haranczyk, M. High accuracy geometric analysis of crystalline porous materials. *CrystEngComm* **15**, 7531–7538 (2013).
32. Dolomanov, O. V., Bourhis, L. J., Gildea, R. J., Howard, J. A. K. & Puschmann, H. OLEX2: A complete structure solution, refinement and analysis program. *J. Appl. Crystallogr.* **42**, 339–341 (2009).
